# Logica: A likelihood framework for cross-ancestry local genetic correlation estimation using summary statistics

**DOI:** 10.1016/j.ajhg.2025.10.001

**Published:** 2025-10-23

**Authors:** Boran Gao, Zheng Li, Xiang Zhou

**Affiliations:** 1 Department of Statistics, Purdue University, West Lafayette, IN 47907, USA; 2 Department of Biological Sciences, Purdue University, West Lafayette, IN 47907, USA; 3 Department of Biostatistics, University of Michigan, Ann Arbor, MI 48109, USA; 4 Center for Statistical Genetics, University of Michigan, Ann Arbor, MI 48109, USA; 5 Department of Statistics and Data Science, Yale University, New Haven, CT 06511, USA

**Keywords:** local genetic correlation, complex traits and diseases

## Abstract

Understanding genetic architecture across ancestries through genetic correlation analysis is critical for determining the degree to which genetic factors underlying diseases or complex traits are shared or differ among populations. Current methods for genetic correlation analysis primarily rely on method of moments approaches and focus on estimating the global genetic correlation across the entire genome. However, these methods often overlook important local genomic complexities and inadequately model the intricate linkage disequilibrium (LD) structures that vary substantially across ancestries. Here, we present Logica (local genetic correlation across ancestries), a method specifically designed to estimate local genetic correlations across ancestries and in admixed populations. Logica employs a bivariate linear mixed model that explicitly accounts for diverse LD patterns across ancestries, operates on genome-wide association study summary statistics, and utilizes a maximum-likelihood framework for robust inference. An important by-product of Logica is a joint heritability test across ancestries that yields well-calibrated *p* values—an aspect that existing approaches often struggle with. We conducted comprehensive evaluations of Logica through realistic simulations and analyses of 13 complex traits from multiple biobanks. Simulations showed that Logica achieves improved accuracy in local genetic correlation estimation (with mean squared errors 2.23–4.13 times lower) and enhanced power for detecting genetically correlated regions (8%–40% increase with controlled false discovery rate [FDR] at 5%). In real data, Logica produced valid genetic correlation estimates across all genomic regions, whereas existing methods failed in 23%–39% of regions. Additionally, Logica exhibited better FDR control (14%–58% improvement), identifying genetically correlated regions with greater functional relevance.

## Teaser

Logica estimates local cross-ancestry genetic correlation from genome-wide association study summary statistics via a bivariate linear mixed model that accounts for ancestry-specific linkage-disequilibrium structure. Across simulations and 13 traits from multi-ancestry biobanks, Logica delivers greater accuracy, power, and false-discovery-rate control and yields valid genome-wide estimates in regions where existing methods fail.

## Introduction

In recent years, there has been a growing number of genome-wide association studies (GWASs) conducted across multiple ancestries. This expansion provides a valuable opportunity to investigate the genetic architecture underlying complex traits in diverse ancestries.^1^^,^^2^^,^^3^^,^^4^^,^^5^ Understanding the genetic architecture across ancestries is crucial for determining the extent to which the genetic basis of a disease or a disease-relevant complex trait is shared or differs between ancestries.^6^^,^^7^^,^^8^ Such insights are essential not only for advancing our biological understanding of complex traits but also for improving the generalizability and equity of genetic research. In particular, knowledge of cross-ancestry genetic architecture informs the transferability of genetic findings—such as effect sizes, polygenic scores, or risk loci—across ancestries, which is particularly important in guiding the translation of clinical findings from one ancestry to another.^7^^,^^9^

An essential analysis for characterizing cross-ancestry genetic architecture is genetic correlation analysis, which assesses the correlation in genetic effect sizes between different ancestries, providing insights into the similarity in genetic architecture across ancestries. Genetic correlation analysis serves as a valuable complement to multi-ancestry fine-mapping approaches, which aim to directly identify causal single-nucleotide polymorphisms (SNPs) within local genomic regions that may be either shared across ancestries or specific to a particular ancestry.^10^^,^^11^^,^^12^^,^^13^ Example methods for genetic correlation analysis include Popcorn and XPASS.^6^^,^^7^ These methods estimate the overall genetic correlation across the entire genome and utilize GWAS summary statistics through the method of moments (MoM). However, estimating the global genetic correlation may overlook important complexities at the level of specific genomic regions, which may harbor distinct sets of genes with varying degrees of genetic sharing across ancestries. As a result, global genetic correlation methods tend to oversimplify the heterogeneous genetic architecture across the genome by collapsing it into a single, aggregate metric, limiting their ability in providing detailed mechanistic insights.^14^^,^^15^^,^^16^ Unfortunately, directly applying existing global genetic correlation methods to individual genomic regions remains challenging for two reasons. First, many of these methods rely on MoM, which considers only the diagonal elements of the squared linkage disequilibrium (LD) matrix, neglecting the complexity of LD structures that vary substantially across ancestries. Second, the standard error estimates produced by these methods, which often rely on resampling approaches, are ineffective, leading to underestimations of the standard error and inflated type I error rates.^14^^,^^15^^,^^16^

**Figure 1:**
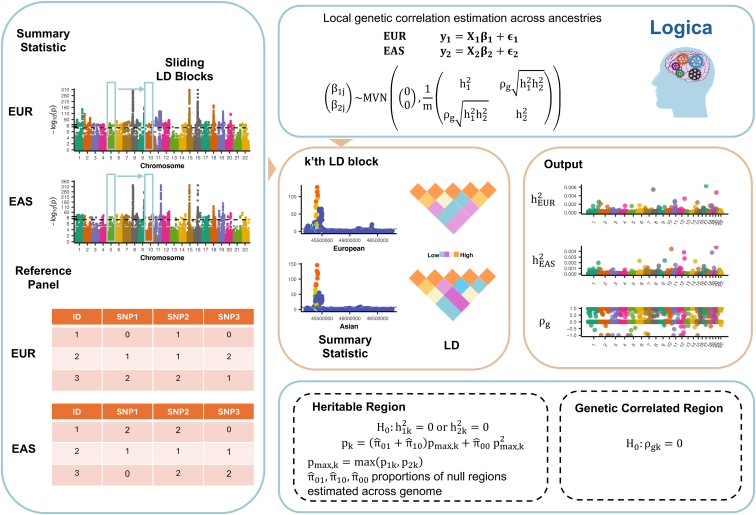
Schematic overview of Logica Logica is a statistical method designed to estimate local genetic correlations across ancestries and detect genetically correlated genomic regions. Logica operates on GWAS summary statistics and scans through LD-independent regions one at a time across the entire genome. For each genomic region, Logica takes as input the *Z* scores from GWAS and ancestry-specific LD matrices calculated from a reference panel. It employs a bivariate linear mixed model to explicitly account for differences in local LD structure across ancestries and performs a likelihood ratio test to detect regions exhibiting significant genetic correlation. As an important by-product, Logica also provides a calibrated hypothesis testing framework for identifying regions with non-zero heritability in both ancestries, which explicitly accounts for the composite nature of the null hypothesis. The outputs of Logica include ancestry-specific local heritability estimates, cross-ancestry local genetic correlation estimates, and their corresponding *p* values.

Here, we introduce a method for estimating local genetic correlation across ancestries, which we have named “Local Genetic Correlation across Ancestries” (Logica). Logica employs a bivariate linear mixed model, explicitly accounts for diverse LD across ancestries, operates on GWAS summary statistics, and relies on the maximum-likelihood framework for inference (Figure 1). As a result, Logica enhances the accuracy of MoM estimates, produces well-controlled false discovery rates (FDRs), and demonstrates greater power in detecting local genetically correlated regions. An important by-product of Logica is its reformulation of a joint heritability test across ancestries, which explicitly accounts for the composite nature of the null hypothesis, resulting in well-calibrated *p* values—an aspect that existing approaches often struggle with. We illustrate the benefits of Logica through comprehensive simulations and real application to 13 traits from the UK Biobank (UKB), Biobank Japan Project (BJP), Korean Biobank Project (KBP), and Taiwan Biobank (TWB).

## Material and methods

### Logica model

We consider a GWAS for a complex trait of interest in the setting where there are two separate GWAS datasets with distinct ancestries. We assume that there are *n*_1_ and *n*_2_ individuals in the two ancestry-specific GWAS datasets, respectively. We denote ***y***_1_ as an *n*_1_ vector of phenotypes measured on *n*_1_ individuals in the first ancestry and ***y***_2_ as an *n*_2_ vector of phenotypes measured on *n*_2_ individuals in the second ancestry. We partition the genome into independent LD blocks following standard procedures.^17^^,^^18^ SNPs within each LD block are correlated with each other in at least one ancestry while SNPs between blocks are approximately independent from each other in both ancestries. We examine one LD block at a time, and we aim to estimate the local heritability of the LD block in each ancestry as well as the genetic correlation of the LD block between two ancestries. For the LD block of focus, we assume that there is a common set of *m* SNPs measured in both ancestries. We denote ***X***_1_ as the *n*_1_-by-*m* genotype matrix for *n*_1_ individuals in the first ancestry and denote ***X***_2_ as the *n*_2_-by-*m* genotype matrix for *n*_2_ individuals in the second ancestry. To facilitate computation, we center and standardize each phenotype vector as well as each column of the genotype matrices to have a mean of zero and a standard deviation of 1 following Gao and Zhou.^13^ We consider the following multivariate regression model to relate genotypes to phenotypes:

(Equation 1) $$\left(\begin{array}{c} y_{1}\\ y_{2} \end{array}\right)=\left[\begin{array}{cc} X_{1} & 0\\ 0 & X_{2} \end{array}\right]*\left(\begin{array}{c} \beta_{1}\\ \beta_{2} \end{array}\right)+\left(\begin{array}{c} \epsilon_{1}\\ \epsilon_{2} \end{array}\right).$$

Here, ***β***_1_ is an *m* vector of SNP effect sizes in the first ancestry; ***β***_2_ is an *m* vector of SNP effect sizes in the second ancestry; ***ϵ***_1_ is an *n*_1_ vector of residual errors with each element following a normal distribution $N\left(0,\sigma_{e1}^{2}I_{n_{1}}\right)$; and ***ϵ***_2_ is an *n*_2_ vector of residual errors with each element following a normal distribution $N\left(0,\sigma_{e2}^{2}I_{n_{2}}\right)$. For the *j*th SNP, we follow previous work^6^^,^^7^^,^^19^^,^^20^^,^^21^^,^^22^ and assume its effect sizes *β*_1*j*_ and *β*_2*j*_ in the two ancestries follow a bivariate normal (BN) distribution:

(Equation 2) $$\left(\begin{array}{c} \beta_{1j}\\ \beta_{2j} \end{array}\right)∼\text{BN}\left(\left(\begin{array}{c} 0\\ 0 \end{array}\right),\left(\begin{array}{cc} \frac{h_{1}^{2}}{m} & \frac{\rho_{g}}{m}\\ \frac{\rho_{g}}{m} & \frac{h_{2}^{2}}{m} \end{array}\right)\right),$$

where $h_{1}^{2}$ and $h_{2}^{2}$ represent the local heritability of trait in the two ancestries, and *ρ*_*g*_ represents the local genetic covariance, which characterizes the phenotypic covariance explained by genetic effects. The genetic correlation is defined as $\gamma_{g}=\rho_{g}/\sqrt{h_{1}^{2}h_{2}^{2}}$.

We propose a parameter expansion expectation maximization (PX-EM) algorithm for the model in Equation 1 to mitigate the slow convergence inherent in the standard EM approach.^23^ Since the convergence rate of the PX-EM algorithm can be sensitive to the choice of initial values, we developed an efficient procedure for parameter initialization by separately estimating $\left(h_{1}^{2},\sigma_{e1}^{2}\right)$ and $\left(h_{2}^{2},\sigma_{e2}^{2}\right)$ via univariate log-likelihood maximization for each ancestry (details in Note S1).

### Hypothesis testing

In the model described above, our goal is to test the null hypothesis that the local heritability for region *k* is zero in at least one of the two ancestries. This hypothesis, however, is technically difficult to test because it is compositional in nature and consists of three mutually exclusive sub-null hypotheses:

$$H_{01,k}:h_{1k}^{2}=0\;\text{and}\;h_{2k}^{2}\neq 0\text{,}$$

$$H_{10,k}:h_{1k}^{2}\neq 0\;\text{and}\;h_{2k}^{2}=0\text{,}$$

$$H_{00,k}:h_{1k}^{2}=0\;\text{and}\;h_{2k}^{2}=0\text{.}$$

To enable a calibrated hypothesis test, we develop an algorithm by extending the recent high-dimensional mediation testing (HDMT) framework, a multiple-testing approach originally introduced for high-dimensional mediation analysis,^24^ toward local heritability testing. Specifically, we first perform ancestry-specific statistical tests to separately evaluate the null hypotheses $h_{1k\;}^{2}=0$ and $h_{2k\;}^{2}=0$. We employ the score test statistic for each of these individual hypotheses, which follows a mixture of chi-squared distributions under the null. From these ancestry-specific tests, we obtain two *p* values, denoted as *p*_1*k*_ and *p*_2*k*_, for each genomic region. Next, we denote *π*_01_, *π*_10_, and *π*_00_ as the proportions of genomic regions corresponding to the three sub-null hypotheses. We estimate *π*_01_, *π*_10_, and *π*_00_ using non-parametric methods^25^ and subsequently construct the composite *p* value for region *k* as^24^^,^^26^

$$p_{k}=\left({\hat{\pi }}_{01}+{\hat{\pi }}_{10}\right)p_{\max,k}+{\hat{\pi }}_{00}\;p_{\max,k}^{2}\text{,}$$

where *p*_max,*k*_ = *p*_1*k*_∨*p*_2*k*_, and the operator ∨ denotes taking the maximum of the two ancestry-specific *p* values. The composite *p* value for region *k* is calibrated with well-controlled FDR and approximately follows a uniform distribution under the null when any of π_01_, π_10_, and π_00_ is close to 1,^26^ a scenario that aligns well with genetic correlation analysis.

Besides testing local heritability, we also test the null hypothesis that the local genetic covariance of the region *k* for the trait between the two ancestries is zero, *H*_0_:*ρ*_*gk*_ = 0. To do so, we employ a likelihood ratio test (LRT), where we first fit the model (Equations 1 and 2) both under the null (*ρ*_*gk*_ = 0) and under the alternative and then calculate the difference in their log likelihoods (*l*(*θ*)): $T_{LRT}=-2\left(l\left({\hat{\theta }}_{alt}\right)-l\left({\hat{\theta }}_{null}\right)\right)$. We then compute the *p* value by assessing the test statistic *T*_*LRT*_ under a chi-squared distribution with 1 degree of freedom ($\chi_{1}^{2}$).

### Expanding on summary statistics

While we have presented our model using individual-level genotype and phenotype data, we note that our model and algorithm are adaptable to utilize GWAS summary statistics exclusively. The required GWAS summary statistics input are in the following forms: the marginal *Z* scores in the two GWASs, in the form of $z_{1}=\frac{X_{1}^{T}y_{1}}{\sqrt{n_{1}}}$ and $z_{2}=\frac{X_{2}^{T}y_{2}}{\sqrt{n_{2}}}$; and the SNP-SNP correlation matrices, also known as the LD matrices, in the two GWASs, in the form of $R_{1}=X_{1}^{T}X_{1}/n_{1}$ and $R_{2}=X_{2}^{T}X_{2}/n_{2}$. These SNP-SNP correlation matrices can also be derived from a reference panel consisting of individuals from the corresponding ancestries. The detailed model and algorithm in the form of summary statistics are provided in Note S1.

Because our algorithm relies on sufficient statistics (*Xᵀy* and *XᵀX*) as input, its computational complexity is independent of the sample size. Instead, it scales cubically with the number of SNPs (*m*) in the focal region, due to the need for eigen-decomposition and inversion of a 2*m* × 2*m* covariance matrix for two ancestries. This contrasts with MoM approaches that use *Z* scores and LD scores as inputs and scale linearly with *m*. Consequently, Logica is expected to be more computationally intensive than MoM approaches, with its cost primarily driven by the number of SNPs and, correspondingly, the size of the LD matrices, rather than the sample size.

We refer to our method as Logica, which is implemented as an R package with underlying efficient C/C++ code linked through Rcpp. The software, together with all the analysis code for reproducing the results presented in the present study, are freely available at https://xiangzhou.github.io/software/.

### Adjusting for population stratification and cryptic relatedness

Population stratification and cryptic relatedness are common sources of bias in heritability and genetic correlation estimates. When individual-level genotype data are available, these issues can be effectively mitigated by regressing the phenotype out of principal components derived from the genetic relationship matrix (GRM) or by excluding individuals with GRM values above a certain threshold.^27^ When only summary statistics data are available, we apply LD eigenvalue regression^28^ to obtain its intercept estimates ${\hat{\lambda }}_{1}$ and ${\hat{\lambda }}_{2}$ for the first and second ancestries as substitutes for $\sigma_{e1}^{2}$ and $\sigma_{e2}^{2}$ to control for these confounding effects. Importantly, we estimate the confounding term using genome-wide information rather than blockwise estimates, reflecting the global nature of population stratification and thus providing a more reliable adjustment.

### Simulations

We performed realistic simulations to evaluate the performance of Logica. Specifically, we first obtained genotype data from a randomly selected ${\tilde{n}}_{EUR}=50,000$ individuals of European ancestry (EUR) in the UKB and obtained genotype data from ${\tilde{n}}_{EAS}$ = 504 individuals of East Asian ancestry (EAS) in the 1000 Genomes Project (1000G) (for details, see the next section, “real data applications”).^5^^,^^29^ We used these genotype data to serve as the reference, with which we simulated summary statistics in the two ancestries for GWASs with a targeted sample size of *n*_*EUR*_ and *n*_*EAS*_, respectively. We set both *n*_*EUR*_ and *n*_*EAS*_ to be 300,000 in the main simulations.

Next, we partitioned the whole genome into 1,368 LD-independent blocks across ancestry and obtained SNPs within these regions that are observed in both ancestries following Lu et al.^17^ We first construct the cross-ancestry LD matrix from the East Asian and European ancestry LD matrices by setting each element of the cross-ancestry LD matrix to the maximum absolute value from the corresponding elements of the East Asian and European LD matrices. The resulting cross-ancestry LD matrix exhibits a block diagonal structure due to shared recombination hotspots across populations. We apply this procedure separately to each chromosome and subsequently use LDetect to define LD blocks within the cross-ancestry LD matrix. This process results in approximately independent LD blocks in individuals of East Asian and European ancestry. These regions ranged in size from 94.8 kb to 16.77 Mb, with an average length of 1.89 Mb and a median of 1.76 Mb. The number of SNPs per region ranged from 85 to 6,350, averaging 2,421 SNPs with a median of 2,367 SNPs.

To mimic realistic genetic architectures, we simulated the genetic effects in each LD-independent block by first categorizing regions into three scenarios: 40% null regions with no heritability in either ancestry, 20% regions heritable in only one ancestry, and 40% regions heritable in both ancestries. For each heritable region, SNP effect sizes were drawn from a bivariate normal distribution under a polygenic model, with local heritability set to either 3 × 10^−4^ or 5 × 10^−4^. Genetic correlations for regions heritable in both ancestries were set uniformly at intervals of 0, 0.25, 0.5, 0.75, and 1.

For each LD-independent block, we extracted the genotypes for the *m* SNPs within that region and simulated the phenotype according to the following procedure. First, we simulated the effect sizes of the SNPs on the trait in the two ancestries from a bivariate normal distribution under polygenic assumption. We denote the SNP effect sizes of all SNPs in the genomic region as *β*_*EUR*_ and *β*_*EAS*_ for the two ancestries, respectively. In addition, we centered and standardized the SNP genotypes in the region in the two ancestries separately and used them to compute the SNP-SNP correlation matrices Σ_*EUR*_ and Σ_*EAS*_ for the two ancestries, respectively. We then followed the strategy of Gao and Zhou^13^ and Wang et al.^30^ to simulate the correlated marginal SNP effect-size estimates that would have been observed on a GWAS with much larger sample sizes of *n*_*EUR*_ and *n*_*EAS*_. In particular, we used the following equations to simulate the marginal SNP effect-size estimates:

$${\hat{\beta }}_{EUR}=\Sigma_{EUR}\beta_{EUR}+\epsilon_{EUR}\text{,}$$

$${\hat{\beta }}_{EAS}=\Sigma_{EAS}\beta_{EAS}+\epsilon_{EAS}\text{,}$$

where *ϵ*_*EUR*_ and *ϵ*_*EAS*_ are the two *m* vectors of estimation errors in the European and East Asian ancestries, respectively. The estimation errors *ϵ*_*EUR*_ and *ϵ*_*EAS*_ are simulated in the following way. Specifically, we first simulated a null phenotype, denoted as ${\tilde{y}}_{WB}$ and ${\tilde{y}}_{EAS}$ in the two ancestries, which are drawn independently from $N\left(0,1-h_{EUR}^{2}\right)$ and $N\left(0,1-h_{EAS}^{2}\right)$, respectively. We then regressed the null phenotypes ${\tilde{y}}_{EUR}$ and ${\tilde{y}}_{EAS}$ on the SNP genotypes to obtain the marginal SNP effect-size estimates ${\tilde{\beta }}_{EUR}$ and ${\tilde{\beta }}_{EAS}$ in the two ancestries. The marginal effect-size estimates ${\tilde{\beta }}_{EUR}$ and ${\tilde{\beta }}_{EAS}$ are further rescaled by the sample-size ratios to obtain the estimation errors in the GWAS with potentially much larger samples: $\epsilon_{EUR}=\sqrt{{\tilde{n}}_{EUR}/n_{EUR}}*{\tilde{\beta }}_{EUR}$ and $\epsilon_{EAS}=\sqrt{{\tilde{n}}_{EAS}/n_{EAS}}*{\tilde{\beta }}_{EAS}$. With the marginal SNP effect-size estimates, we further compute the marginal *Z* scores as $z_{EUR}={\hat{\beta }}_{EUR}/se_{EUR}$ and $z_{EAS}={\hat{\beta }}_{EAS}/se_{EAS}$, where $se_{EUR}=1/\sqrt{n_{EUR}}$ and $se_{EAS}=1/\sqrt{n_{EAS}}$ due to centering and standardization of the genotype data. The above simulation strategy allows us to simulate summary statistics that are correlated due to LD from much larger sample sizes given a limited number of available samples.

We treat the above simulation setting as the baseline setting. We further assessed the robustness of our approach by examining different initializations under baseline simulation setting. We considered two alternatives: (1) MoM estimates from XPASS (“Logica MoM”) and (2) random initial estimates (“Logica Random”). For Logica Random, local heritability initial values were generated by first drawing from a uniform distribution over −20 to −4 using the runif function in R, then transforming by 10^*x*^ to yield values between 10^−20^ and 10^−4^. Local genetic correlation initial values were drawn from a uniform distribution over −0.99 to 0.99. We then applied the Logica MoM and Logica Random to the baseline simulation settings to evaluate the consistency in the performance of our method under these different initialization values.

On top of the baseline setting, we varied one parameter at a time to explore five alternative simulation scenarios. Specifically, we examined a sparse genetic architecture scenario where 10% and 50% of SNPs were randomly selected to have non-zero effects in the two ancestries, respectively. Additionally, we investigated the impact of unadjusted population stratification by incorporating the top ten principal components with effect variance set to 0.1. We explored an imbalanced sample size setting where we set *n*_*EAS*_ to be 100,000 instead of 300,000. We also examined the influence of using a much smaller LD reference panel on method performance by randomly sampling 5,000 individuals of European ancestry from *X*_*EUR*_ and 200 individuals of East Asian ancestry from *X*_*EAS*_ to compute the SNP-SNP correlation matrices Σ_*EUR*_ and Σ_*EAS*_ following Zou et al.^31^^,^^32^ and Weissbrod et al.^31^^,^^32^ To further assess robustness with smaller reference panels, we additionally considered 503 European individuals and 504 East Asian individuals from 1000G to compute the SNP-SNP correlation matrices. In total, we examined six simulation scenarios (one baseline and five alternatives), with 1,368 LD-independent regions per scenario. We provide the detailed parameter settings for each simulation scenario in Table S1.

In each simulation replicate, we first computed the marginal *Z* scores and the SNP-SNP correlation matrices in each ancestry. We then fitted Logica and two global genetic correlation estimation methods Popcorn and XPASS.^6^^,^^7^ For Popcorn, we first computed the LD score using the “compute” option, followed by the estimation of genetic correlation with the “fit -v” option. Subsequently, we processed the output from Popcorn to assess the presence of non-zero genetic correlation across ancestries. By default, Popcorn tests the hypothesis that the genetic correlation is 1. To evaluate the hypothesis of non-zero genetic correlation, we utilized both the estimated genetic correlation and its standard error, the latter of which is derived through a bootstrapping approach. For XPASS, we employed the XPASS function, specifying sd_method = “Jackknife” to calculate the standard error of estimated genetic correlation. Similarly, we used both the estimated genetic correlation and its standard error to conduct tests for the null hypothesis that the genetic correlation is zero.

In the simulations, we assessed estimation accuracy of local heritability, genetic covariance, and genetic correlation by calculating the mean squared error (MSE) of estimates derived from various methods. Furthermore, we evaluated Logica’s performance across various simulation scenarios by assessing its statistical power under two testing objectives: (1) identifying genomic regions exhibiting non-zero local heritability in both ancestries and (2) detecting genomic regions that exhibit non-zero genetic correlations across ancestries. Given that hypothesis testing was conducted across 1,368 LD-independent genomic regions, we accounted for multiple-testing issues by applying the Benjamini-Hochberg (BH) procedure to control the FDR at a nominal level of 0.05. Following this adjustment, we evaluated each method’s empirical FDR and power. To make fair comparison, we also compared the power of these methods by setting FDR at 0.05.

### Real data applications

We applied our method to analyze 13 complex traits spanning four categories (Table S2), utilizing summary statistics from individuals of European ancestry in the UKB and individuals of East Asian ancestry from the BJP,^33^ KBP,^34^ and TWB.^35^ These traits included anthropometric traits (height), hematological traits (red blood cell count [RBC], hemoglobin, platelet count [PLT], hematocrit, and white blood cell count), metabolic traits (high-density lipoprotein [HDL], low-density lipoprotein [LDL], triglycerides, and total cholesterol), and liver-related traits (alanine aminotransferase, aspartate aminotransferase, and albumin).

The UKB GWAS summary statistics were sourced from the second round of results released in August 2018 by Neale’s group (https://docs.google.com/spreadsheets/d/1kvPoupSzsSFBNSztMzl04xMoSC3Kcx3CrjVf4yBmESU/edit?gid=227859291#gid=227859291). This dataset includes marginal GWAS *Z* scores for 13,791,467 SNPs derived from 361,194 individuals of European ancestry. We further filtered out SNPs with minor allele frequency (MAF) <0.05, that are strand ambiguous, that are multi-allelic, or in the human leukocyte antigen (HLA) regions (chr6: 24–36 Mb) due to the complexity of the region and kept only SNPs imputed by the Haplotype Reference Consortium reference panel following previous work^13^ to retain a total of 4,995,795–4,995,959 SNPs for analysis.

The BJP GWAS summary statistics were obtained from the BJP (https://pheweb.jp/downloads), covering marginal GWAS *Z* scores for 13,236,464 SNPs derived from 72,866–165,056 individuals. The KBP GWAS summary statistics were sourced from the KBP (https://zenodo.org/records/7042518), including 8,056,212 SNPs from 49,874–72,297 individuals. The TWB GWAS summary statistics were obtained from the TWB (http://ftp.ebi.ac.uk/pub/databases/gwas/summary_statistics/), containing 8,238,970 SNPs from 92,615 individuals. For each dataset, SNPs with MAF <0.05, imputation information <0.8, strand ambiguity, or multi-allelic variants were excluded, resulting in a common subset of 3,829,633 SNPs across the three biobanks. Marginal *Z* scores from each GWAS were used as input, and we conducted a meta-analysis across datasets using METAL^36^ to generate combined GWAS summary statistics.

In addition to the GWAS data, we employed genotype information from the UKB and 1000G to construct reference panels for European and East Asian ancestries, respectively. From the UKB, which includes data on 487,409 individuals, we selected White British individuals for the European ancestry reference panel. We excluded individuals not accounted for in genotype principal component analysis as well as those with sex chromosome aneuploidy or any redacted entries, resulting in 337,198 individuals. Among the remaining individuals, we randomly sampled 50,000 individuals to serve as the genotype reference panel for European ancestry. For these individuals, we performed SNP quality control (QC) by filtering out variants with a MAF <5%, with an imputation information score ≤0.8, with a Hardy-Weinberg equilibrium (HWE) test *p* value <10^−7^, with a genotype call rate <0.95, or that are indels. We also kept only SNPs imputed by the Haplotype Reference Consortium reference panel, retaining a total of 4,362,261 SNPs. For the East Asian reference panel, we utilized data of 504 East Asian individuals from 1000G. The SNP QC for this group involved filtering out variants with a MAF <5%, with an HWE test *p* value <10^−7^, with a genotype call rate <0.95, or indels, retaining in a total of 4,611,974 SNPs. After applying these QC measures, we retained for analysis a total of 2,945,669 SNPs that are common between the GWAS summary statistics and the reference panels for both ancestries.

Following Lu et al.,^17^ we split the whole genome into approximately independent, non-overlapping genomic regions across European and East Asian ancestries (https://github.com/mancusolab/ma-focus/tree/master/pyfocus/data/ld_blocks). In total, we obtained 1,357 approximately independent LD blocks, with the regional length ranging from 107.5 kb to 25.85 Mb (mean = 1.96 Mb; median = 1.80 Mb) and the number of SNPs per region ranging from 82 to 6,045 (mean = 2,168 SNPs; median = 2,115 SNPs). For each trait, we examined one genomic region at a time and used both marginal *Z* scores and SNP-SNP correlation matrices as input for all methods to estimate local genetic correlation. The SNP-SNP correlation matrices for the two ancestries are constructed using the cor() function in R.

In the analysis, we first applied Logica to identify genomic regions showing significant non-zero heritability and genetic correlation (BH corrected *p* value <0.05). We assessed the functional significance of the regions identified by Logica through enrichment analysis on SNPs within the genetically correlated regions. First, we obtained variant functional annotations predicted using Ensembl Variant Effect Predictor (VEP v.85; GRCh37, GENCODE v.19),^37^ categorizing SNPs by their potential impact (high, moderate, low, or modifier). We then tested whether SNPs classified as high or moderate impact were enriched within the regions vs. outside using the chi-squared test. We further compared the enrichment results for regions identified by Logica vs. those identified by the other methods. Second, we examined whether expression quantitative trait loci (eQTLs) were enriched within the regions identified by Logica, leveraging significant independent blood eQTLs from the Genotype-Tissue Expression project.^38^ SNPs genome-wide were annotated as eQTL or non-eQTL, and enrichment analyses were conducted via chi-squared tests to examine whether eQTL SNPs were over-represented within genetically correlated regions.

To further explore the biological significance of genetically correlated regions identified by Logica, we stratified the analyzed genomic regions into three categories: (1) regions with zero heritability in both ancestries; (2) regions with non-zero heritability in only one ancestry, or regions with non-zero heritability in both ancestries but without significant genetic correlation; and (3) regions with non-zero heritability in both ancestries and significant genetic correlation. Regions were stratified using a two-step testing procedure. First, we tested for non-zero heritability separately in each ancestry, declaring a region as heritable if the BH-adjusted *p*_*k*_ was less than the nominal threshold of 0.05. Second, we tested for non-zero local genetic covariance using an LRT for the null hypothesis *ρ*_*gk*_ = 0, obtaining a *p* value *p*_*ρk*_ for *k*th region. Combining these tests, we declare a region as having non-zero heritability in both ancestries but zero genetic correlation if, for region *k*, BH-adjusted *p*_*k*_ < 0.05 and BH-adjusted *ρ*_*gk*_ > 0.05. We compared the functional enrichments of SNPs across these categories to determine whether the genetically correlated regions identified by Logica exhibit stronger evidence of functional relevance compared to either regions with non-zero heritability but zero genetic correlation or non-heritable regions.

## Results

### Simulation studies

We conducted comprehensive simulations under realistic scenarios to evaluate the performance of Logica and compared it with two established global genetic correlation methods, Popcorn and XPASS. The detailed simulation framework and parameter settings are provided in material and methods. In brief, we considered a total of six distinct simulation scenarios, each consisting of 1,368 LD-independent genomic regions characterized by varying degrees of heritability, genetic correlations, causal SNP proportions, presence or absence of confounding factors, sample sizes across ancestries, and use of external reference panels.

For local heritability estimation, in the baseline setting under a fully polygenic genetic architecture, all methods provide unbiased estimates of local heritability across varying genetic correlation levels (Figure S1). Logica consistently yielded the more accurate heritability estimates, achieving the lowest mean MSE of 1.76 × 10^−8^ (range: 2.19 × 10^−27^ to 9.61 × 10^−7^), followed by XPASS with a mean MSE of 2.60 × 10^−8^ (range: 9.57 × 10^−12^ to 7.53 × 10^−7^) and Popcorn with a mean MSE of 8.36 × 10^−8^ (range: 1.58 × 10^−13^ to 2.50 × 10^−6^) (Figure S1). When testing the composite null hypothesis—that at least one region has zero heritability in at least one ancestry—Logica and Popcorn maintain well-controlled FDR at 0.028 and 0.05, respectively, whereas XPASS exhibits an inflated FDR of 0.51 at a BH-adjusted *p* value threshold of 0.05. Logica also achieves the highest detection power (0.996) among methods maintaining FDR control, followed by Popcorn (0.0691). Although XPASS shows comparable power (0.998), its FDR control is not under control (Figure S2).

**Figure 2:**
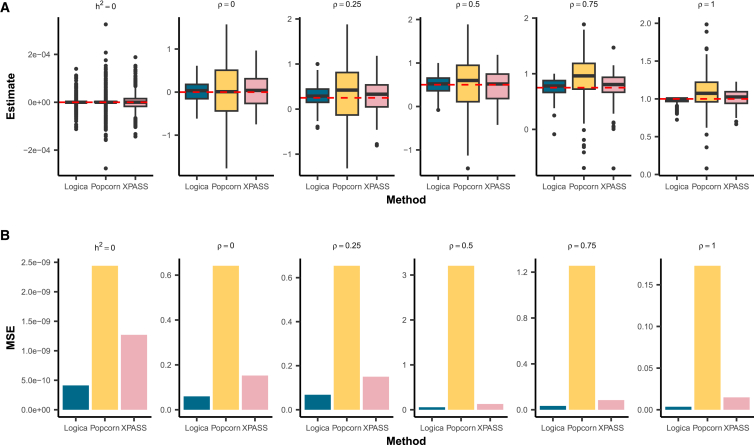
Comparison of methods for local genetic correlation estimation accuracy Results are shown for the baseline simulation setting with *n* = 1,368 independent genomic regions. The simulated sample size for both ancestries is set to 300,000, with in-sample LD matrices used for model fitting. Among these regions, 40% have zero heritability in both ancestries, 20% have non-zero heritability in only one ancestry (with equal probability for each), and 40% have non-zero heritability in both ancestries. The heritability of these regions is set to either 3 × 10^−5^ or 5 × 10^−5^ with equal probability. For regions exhibiting non-zero heritability in both ancestries, the true genetic correlation is set to 0, 0.25, 0.5, 0.75, or 1 with equal probability. From left to right, columns represent scenarios with zero heritability in at least one ancestry (*h*^2^ = 0) and scenarios with non-zero heritability in both ancestries, each varying by genetic correlation (*ρ* = 0, 0.25, 0.5, 0.75, or 1). Because genetic correlation is undefined when *h*^2^ = 0, the left column reports the cross-ancestry covariance. We compare the performance of Logica (blue) against Popcorn (yellow) and XPASS (pink). (A) Boxplot of the estimated local genetic correlations for each method. Red dashed lines indicate the true genetic correlation values. (B) Barplots of the mean squared error (MSE) of the estimates, comparing the performance of each method across different true genetic correlation values. Corresponding numerical summaries are provided in Table S3.

**Figure 3:**
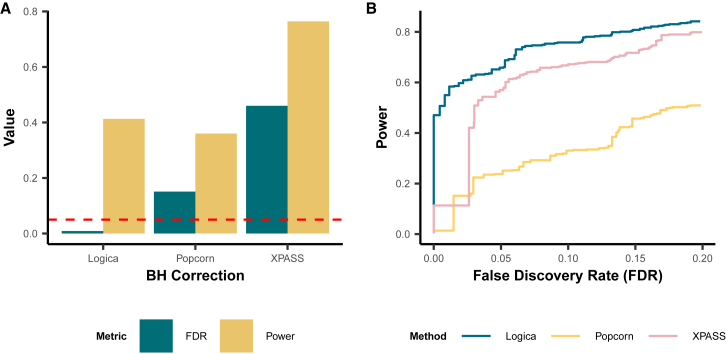
Comparison of methods for false discovery rate control and power in detecting local genetically correlated regions across ancestries Results are shown for the baseline simulation setting. A region is declared as significant if its Benjamini-Hochberg (BH)-adjusted *p* value is below a specified threshold. Power is defined as the proportion of truly genetically correlated regions that are correctly detected. False discovery rate (FDR) is defined as the proportion of falsely detected regions among all detected regions. (A) Comparison of FDR (dark green) and power (yellow) among methods using a BH-adjusted *p* value threshold of 0.05. The dashed red line indicates the nominal FDR level of 0.05. (B) FDR-power trade-off plot comparing the performance of Logica (blue), Popcorn (yellow), and XPASS (pink) across different FDR thresholds, allowing fair comparison of power at comparable FDR levels.

For genetic correlation estimation, in the baseline setting under a fully polygenic genetic architecture, Popcorn and XPASS encountered substantial estimation difficulties, failing to produce valid genetic correlation estimates in 34.7% (474 of 1,368) and 31.6% (432 of 1,368) of the genomic regions, respectively (Figure S3). These estimation failures primarily occurred when the two MoM-based methods yielded negative heritability estimates, resulting in undefined genetic correlation estimates. Despite these failures, in the succeed regions, all methods provided unbiased genetic correlation estimates across different heritability and genetic correlation settings (Figure 2). To systematically evaluate estimation accuracy, we calculated the MSE of genetic correlation estimates for each method. In regions where both ancestries had non-zero heritability, Logica consistently yielded more accurate genetic correlation estimates, achieving the lowest mean MSE of 0.044 (range: 0.0036–0.068), followed by XPASS with a mean MSE of 0.11 (range: 0.0149–0.15) and Popcorn with a mean MSE of 1.19 (range: 0.173–3.21) (Figure 2). In regions where at least one ancestry had zero heritability, genetic correlation is not defined; in these cases, we evaluated genetic covariance. Logica achieved the lowest MSE (4.1 × 10^−10^), followed by XPASS (1.3 × 10^−9^) and Popcorn (2.4 × 10^−9^). Additionally, we observed a decreasing trend in MSE values for all methods as the genetic correlation increased in regions exhibiting non-zero heritability in both ancestries. Logica is the only method that maintains well-controlled FDR (0.008), whereas Popcorn and XPASS exhibit inflated FDRs of 0.15 and 0.46, respectively, at a BH-adjusted *p* value threshold of 0.05. Under the BH-adjusted *p* value threshold of 0.05, Logica achieves a power of 0.413, higher than Popcorn (0.36) but lower than XPASS (0.764) (Figure 3A). To ensure a fair comparison, we further compared the power of different methods at the same controlled FDR threshold of 0.05 and found that Logica achieves the highest power (0.658), followed by XPASS (0.57) and Popcorn (0.251) (Figure 3B). We further evaluated the robustness of Logica to initialization choices. Logica produces consistent local heritability estimates across different initializations and that local genetic correlation estimates are stable when using MoM initialization. While random initialization led to reduced accuracy and higher MSE for local genetic correlation, the MoM and original univariate initializations yielded comparable accuracy (for details, see Note S2).

We carefully examined the influence of various factors on method performance. First, we estimated the impact of the proportion of causal SNPs on the local genetic correlation estimation and testing, focusing on regions with non-zero heritability in both ancestries, where genetic correlation is well defined. We found that Logica’s estimation accuracy remains stable regardless of the causal SNP proportion, exhibiting consistently low mean MSE values (0.044, 0.050, and 0.048 for causal SNP proportions at 100%, 50%, and 10%, respectively), outperforming alternative approaches (Figures S4 and S5). By comparison, Popcorn and XPASS consistently showed higher mean MSEs across different causal SNP proportions (Popcorn: 1.19, 0.47, and 0.66, respectively; XPASS: 0.11, 0.11, and 0.10, respectively). Furthermore, Logica consistently maintained well-controlled FDR and demonstrated higher statistical power even under increasing sparsity. Specifically, at a nominal *p* value cutoff of 0.05, Logica’s FDR remained low, slightly increasing from 0.0128 (at 50% causal SNPs) to 0.0224 (at 10% causal SNPs). In contrast, Popcorn and XPASS exhibited substantial inflation in FDR across these sparsity conditions (Popcorn: 0.142 and 0.172, respectively; XPASS: 0.45 and 0.46, respectively). Logica also achieved consistently high power in detecting genetically correlated regions at BH-adjusted *p* value threshold of 0.05, with power values of 0.523 and 0.493 at 50% and 10% sparsity, respectively. Popcorn had considerably lower power (0.396 at 50% and 0.380 at 10%), whereas XPASS exhibited higher power (0.946 at 50% and 0.957 at 10%). To ensure a fair comparison, we further compared the power of different methods at the same controlled FDR level of 0.05. At this threshold, Logica consistently achieved the highest power (0.658 in the fully polygenic scenario, 0.656 at 50% sparsity, and 0.633 at 10% sparsity). In contrast, both Popcorn and XPASS showed decreased power with increased sparsity (XPASS: 0.57 decreasing to 0.514 and 0.466; Popcorn: 0.268 decreasing to 0.0976 and 0.130; Figures S6 and S7).

Second, we evaluated scenarios with unbalanced ancestry proportions, where the European sample size was three times larger than the East Asian sample size (sample size ratio = 3:1). In this unbalanced setting, although the relative performance ranking among methods remained consistent, we observed a decrease in both estimation accuracy and power across all methods. Specifically, Logica’s mean MSE increased from 0.044 (equal sample sizes) to 0.077, while Popcorn and XPASS exhibited higher mean MSE values of 2.31 and 0.18, respectively (Figure S8). Furthermore, all methods showed elevated FDR and reduced power. At a nominal BH-adjusted *p* value threshold of 0.05, Logica maintained FDR control (FDR = 0) with a power of 0.36, whereas Popcorn and XPASS exhibited FDR inflation (0.161 and 0.461) with power of 0.305 and 0.930, respectively. At a controlled FDR of 0.05 that ensures fair comparison, Logica achieved the highest power (0.559), outperforming XPASS (0.402) and Popcorn (0.160) (Figure S9).

Third, we investigated scenarios with population stratification. Although the relative ranking among methods remained consistent, we observed a general reduction in estimation accuracy. Specifically, Logica’s mean MSE slightly increased from 0.044 (no stratification) to 0.046 under population stratification scenarios, whereas Popcorn and XPASS exhibited higher mean MSE values of 1.18 and 0.11, respectively (Figure S10). Furthermore, competing methods displayed inflation in FDR and reductions in power. At a nominal BH-adjusted *p* value threshold of 0.05, Logica maintained well-controlled FDR (0.0044) and achieved a power of 0.413. In contrast, Popcorn and XPASS exhibited inflated FDRs (0.152 and 0.505, respectively), with corresponding power values of 0.152 and 0.761. At a controlled FDR of 0.05 that ensures fair comparison, Logica demonstrated the highest power (0.652), clearly outperforming XPASS (0.577) and Popcorn (0.251) (Figure S11).

Finally, we explored scenarios involving the use of an external reference panel. Under these conditions, Logica maintained a consistently low mean MSE (0.044), whereas Popcorn and XPASS exhibited higher mean MSE values of 0.92 and 0.11, respectively (Figure S12). When controlling FDR at 0.05, all methods displayed power patterns consistent with previous scenarios: Logica achieved the highest power (0.66), followed by XPASS (0.57) and Popcorn (0.20) (Figure S13).

### Computation time and memory usage

To validate the theoretical computational complexity of Logica, we evaluated the computational cost in baseline simulations across 1,368 genomic regions, with SNP counts per region ranging from 85 to 6,350 (mean: 2,421 SNPs; median: 2,367 SNPs). On average, Logica required 5.95 min per region (range: 0.008–65.20 min), compared to 0.01 min for XPASS (range: 0.001–0.05 min) and 0.08 min for Popcorn (range: 0.029–0.44 min). The runtime of Logica scaled cubically with the number of SNPs (Figure S19), consistent with its reliance on maximum-likelihood-based inference. Although Logica is slower than XPASS and Popcorn, its computational cost remains moderate: with parallelization across 100 cores, Logica analysis on the entire genome can be completed in approximately 81.4 min. To further improve efficiency, we implemented a two-step screening approach during the revision. This two-step strategy first identifies regions with non-zero heritability in both ancestries, limiting the more intensive genetic correlation estimation to only these regions. This two-step strategy reduces the average computing time per region from 5.95 min to 4.60 min. In terms of peak memory usage, Logica averaged 1,338 MB per region, compared to 259 MB for XPASS and 174 MB for Popcorn. The maximum peak memory observed was 6,724 MB for the largest region with 6,350 SNPs, and memory usage similarly scaled cubically with SNP count (Figure S20).

### Real data applications

We applied Logica and other methods to estimate the local genetic correlation of 13 traits between European and East Asian ancestries. For each trait, we analyzed 1,357 LD-independent regions, employing GWAS summary statistics from 361,194 individuals of European ancestry from UKB and a combined total of 237,613–330,318 East Asian individuals from the BJP, KBP, and TWB via meta-analysis. The LD reference panels used were from European individuals in the UKB and East Asian individuals in the 1000G genotype data (for details, see material and methods).

**Figure 4:**
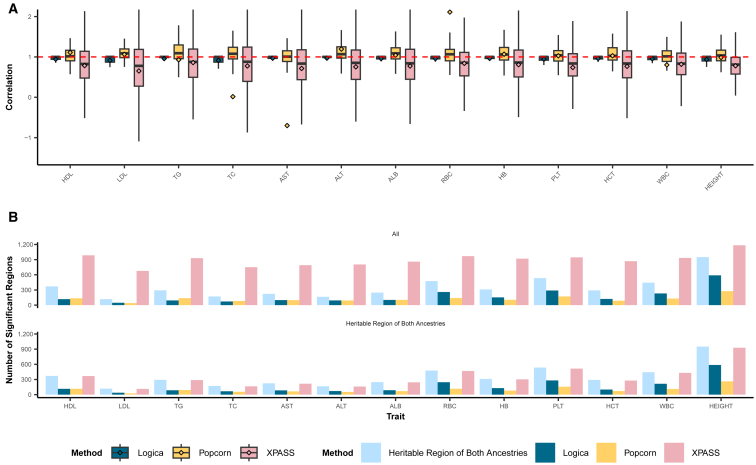
Genetic correlation estimates across ancestries for 13 complex traits in the real data application (A) Boxplots show the estimated local genetic correlations from detected regions across 13 traits using Logica (blue), Popcorn (yellow), and XPASS (pink). Dashed red line represents the local genetic correlation value of 1. (B) Top panel displays the number of genomic regions detected as heritable in both ancestries by Logica (dark blue), and regions detected as genetically correlated by Logica (dark blue), Popcorn (yellow), and XPASS (pink) across multiple traits. Bottom panel displays the number of genomic regions detected as heritable in both ancestries by Logica (dark blue) and the subset of genetically correlated regions that overlap with these heritable regions identified by Logica (dark blue), Popcorn (yellow), and XPASS (pink).

We first evaluated Logica’s performance in genetic correlation estimation and compared it with other methods. Consistent with simulations, unlike Logica, Popcorn and XPASS failed to produce valid genetic correlation estimates in a large fraction of genomic regions, while Logica produced estimates for all regions. Specifically, Popcorn failed to provide estimates for an average of 529 out of 1,357 (39.0%; range: 391–611 across traits) genomic regions, while XPASS failed for an average of 317 regions (23.4%; range: 85–478 across traits) (Figure S14). Overall, Logica identified an average of 175 genetically correlated regions (range: 47–589 across traits), Popcorn identified 123 regions (range: 40–276 across traits), and XPASS identified a substantially larger number of regions, averaging 894 (range: 677–1,184 across traits), consistent with its high false discoveries observed in simulations. The mean genetic correlation across regions identified by Logica was consistently high, ranging from 0.91 to 0.97 across traits, with a mean of 0.96 (Figure 4A). In contrast, Popcorn exhibited substantial variability, with mean genetic correlations ranging widely from −0.70 to 2.11, with an overall mean of 0.89. XPASS produced moderate genetic correlation estimates, with means ranging from 0.65 to 0.86 across traits. While the true genetically correlated regions are unknown, we reasoned that such regions would exhibit non-zero heritability in both genetic ancestries. Therefore, we obtained an average of 353 regions across traits (range: 118–950) identified by Logica to exhibit non-zero heritability in both ancestries and examined whether these regions are. Indeed, among the genetically correlated regions identified by Logica, an average of 93% of them (162/175; range: 47–589) displayed non-zero heritability in both traits. Such percentage is lower for Popcorn (79%; 98/123; range: 40–276) and much lower for XPASS (38%; 344/894; range: 113–927). Notably, XPASS identified considerably more correlated regions than those with non-zero heritability in both ancestries, suggesting a high rate of false discoveries. These results support the superior performance of Logica (Figure 4B).

**Figure 5:**
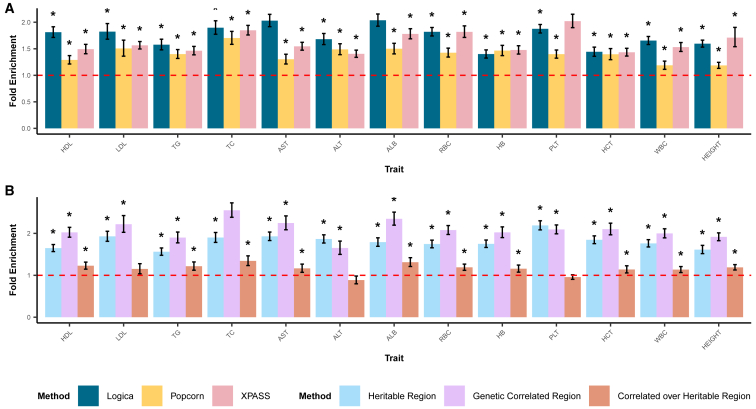
Fold enrichment of SNP impact in detected genetically correlated regions across 13 traits (A) SNP impact fold enrichment is calculated as the ratio of high-/moderate-impact SNPs within the detected genetically correlated regions to those outside such regions. A chi-squared test is used to evaluate statistical significance of enrichment, with asterisks indicating traits with significant SNP impact enrichment. The dashed red line represents fold enrichment value of 1. (B) Regions are grouped based on Logica’s testing results into three categories: (1) regions with zero heritability in both ancestries; (2) regions with non-zero heritability in only one ancestry or in both ancestries but without significant genetic correlation; and (3) regions with non-zero heritability in both ancestries and significant genetic correlation. We performed SNP impact enrichment analysis separately for each category pair: category 2 vs. category 1, category 3 vs. category 2, and category 3 vs. category 1. The dashed red line represents fold enrichment value of 1.

Next, we assessed the functional relevance of the genetically correlated regions through SNP impact enrichment and eQTL enrichment analyses. We found that SNPs within regions identified by Logica were enriched for high or moderate impact variants in the majority of traits (10 out of 13), with average enrichment values ranging from 1.40 to 2.04 (overall mean across 13 traits = 1.74). SNPs within regions identified by XPASS showed high enrichment in three (out of 13) traits, with average enrichment values ranging from 1.40 to 2.02 (overall mean = 1.62). In comparison, SNP impact enrichment for Popcorn ranged from 1.19 to 1.70, with an overall mean of 1.40 across 13 traits (Figure 5A). Additionally, SNPs within the regions identified by Logica were also enriched with eQTLs in 8 out of 13 traits, with average enrichment values ranging from 1.63 to 2.15 (mean = 1.91). The SNPs within the regions identified by XPASS were enriched with eQTLs in five traits, with enrichment values ranging from 1.51 to 2.48 (mean = 1.89), while those for Popcorn ranged from 1.28 to 1.95 (mean = 1.59; Figure S15). These functional enrichment analyses further validate that the regions identified by Logica have greater functional relevance compared to regions identified by the other methods.

We further conducted stratified enrichment analysis to assess the biological relevance of the genetically correlated regions. Specifically, we stratified genomic regions into three categories and performed enrichment analysis in each category separately. The three categories include (1) regions with zero heritability in both ancestries (44.1%; range: 11.2%–70.9%); (2) regions with non-zero heritability in only one ancestry, or regions with non-zero heritability in both ancestries but without significant genetic correlation (39.9%; range: 25.1%–52.5%); and (3) regions with non-zero heritability in both ancestries and significant genetic correlation (16.0%; range: 4.1%–45.1%). We found that SNPs within regions of categories 2 and 3 showed stronger SNP impact enrichment compared to regions in category 1 (mean = 1.44, range 1.33–1.66 for category 2; mean = 2.39, range 1.93–2.90 for category 3, across traits). Furthermore, SNPs within category 3 regions showed an average of 1.65-fold enrichment (range: 1.39–2.17) compared directly to SNPs within category 2 regions (Figure 5B). Similarly, SNPs within regions of categories 2 and 3 showed stronger eQTL enrichment compared to regions with zero heritability in both ancestries (mean = 1.56, range 1.33–1.77 for category 2; mean = 3.05, range 2.47–4.38 for category 3, across traits). SNPs within category 3 regions also showed an average of 1.96-fold eQTL enrichment (range: 1.61–2.52) compared directly to SNPs within category 2 regions, highlighting the enhanced biological significance of genetically correlated regions identified by Logica (Figure S16).

**Figure 6:**
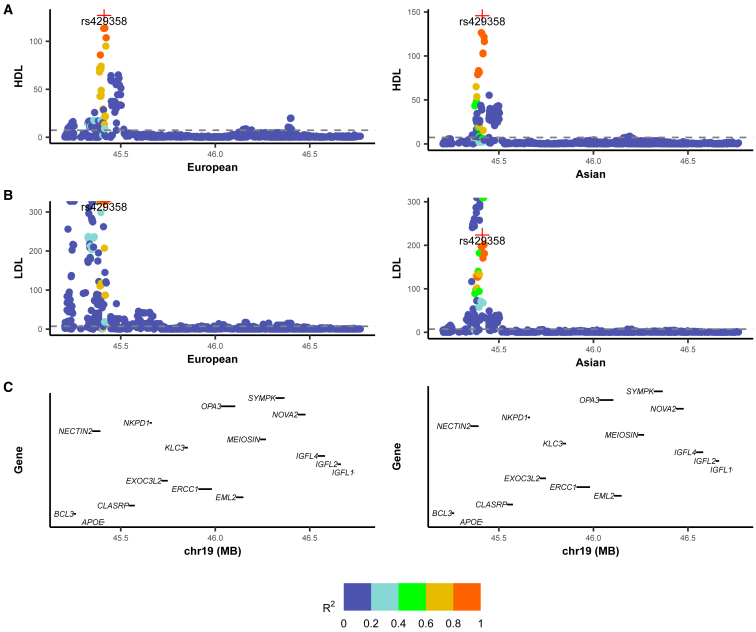
Genetic correlation analysis of HDL and LDL in a genomic region on chromosome 19 (A) LocusZoom plots showing marginal GWAS results (−log_10_*p* value, *y* axis) for HDL (left column) and LDL (right column) across base-pair positions (*x* axis) in European ancestry. SNP colors indicate linkage disequilibrium (LD, measured as *R*^2^) with the lead variant rs429358. (B) LocusZoom plots of marginal GWAS results for HDL (left) and LDL (right) in East Asian ancestry, with the same color scheme and LD reference variant. (C) Gene annotations within the genomic region, highlighting the candidate gene *APOE*.

We highlight several example regions to illustrate the advantages of Logica. The first example involves a genetically correlated region for LDL, located at 43.1–44.6 Mb on chromosome 19. Within this region, SNP rs429358 exhibits strong association signals in both European (*Z* score = 59.72, *p* value ≈ 0) and East Asian (*Z* score = 17.87, *p* value = 1.93 × 10^−71^) ancestries (Figure 6). rs429358 is a missense variant within *APOE*, a critical gene implicated in lipid metabolism.^39^ While the marginal effect-size correlation of SNPs within the region across ancestries is relatively modest at 0.19, Logica estimated a considerably higher conditional effect-size correlation of 0.63, suggesting stronger correlation among potential causal SNPs in the region. To validate this interpretation, we applied MESuSiE, a multi-ancestry fine-mapping method, to this region. MESuSiE identified ten credible sets, among which nine were shared between ancestries, indicating strong causal overlap. Additionally, among the ten SNPs with posterior inclusion probabilities (PIPs) greater than 0.5, eight represented shared causal signals. Taken together, the collective evidence from conditional effect-size correlations, multi-ancestry fine-mapping, and the known functional role of *APOE* supports the presence of true genetic correlation across ancestries in this region. Consistently, Logica confirmed significant genetic correlation in this region (*γ*_*g*_ = 0.48, *p* value = 1.56 × 10^−18^), whereas XPASS (*γ*_*g*_ = 0.22, *p* value = 0.007) and Popcorn (*γ*_*g*_ = −0.005, *p* value = 0.99) failed to detect such a signal. Importantly, this region also exhibited significant genetic correlation across ancestries for additional traits. For instance, it displayed significant genetic correlation for HDL, with SNP rs429358 again showing significant associations in both European (*p* value = 9.42 × 10^−128^) and East Asian (*p* value = 2.87 × 10^−30^) ancestries and a high marginal effect-size correlation across ancestries (0.51). Both Logica (*γ*_*g*_ = 0.94, *p* value = 3.13 × 10^−14^) and XPASS (*γ*_*g*_ = 0.84, *p* value = 2.15 × 10^−121^) identified the region as genetically correlated, while Popcorn did not (*ρ* = 1.15, *p* value = 0.62).

The second example involves a region (51.1–53.5 Mb on chromosome 12) with significant genetic correlation for PLT. Within this region, SNP rs10876550 represents the peak GWAS signal for PLT in European (*Z* score = 22.40, *p* value = 4.86 × 10^−111^) and East Asian (*Z* score = 13.32, *p* value = 2.42 × 10^−39^) ancestries (Figure S17). Notably, rs10876550 resides near *NFE2*, a key transcription factor essential for platelet formation.^40^ Although the marginal effect-size correlation of SNPs across ancestries is modest at 0.10, Logica identified a considerably higher conditional effect-size correlation of 0.96. MESuSiE analysis identified five credible sets, among which three were shared credible sets. Additionally, among the four SNPs with PIP greater than 0.5, two represented shared causal signals between ancestries. Logica confirmed significant genetic correlation for PLT in this region (*γ*_*g*_ = 0.95, *p* value = 8.4 × 10^−5^), whereas Popcorn (*γ*_*g*_ = −1.36, *p* value = 0.73) and XPASS (*γ*_*g*_ = 0.01, *p* value = 0.95) failed to identify significant genetic correlation. Importantly, this region was also found to exhibit genetic correlation across ancestries for additional traits. For instance, the same region displayed significant genetic correlation for RBC, with SNP rs10876550 again showing robust associations in both European (*Z* score = 6.22, *p* value = 4.90 × 10^−10^) and East Asian (*Z* score = 5.09, *p* value = 2.68 × 10^−7^) ancestries. The marginal effect-size correlation was higher for RBC (0.33 across ancestries). Here, both Logica (*γ*_*g*_ = 0.95, *p* value = 8.4 × 10^−5^) and XPASS (*γ*_*g*_ = 0.56, *p* value = 1.2 × 10^−21^) identified the region as genetically correlated, while Popcorn did not (*γ*_*g*_ = 1.01, *p* value = 0.67).

The final example examines a potentially null region spanning 69.8–72.2 Mb on chromosome 10, which does not appear to exhibit genetic correlation for LDL. Specifically, the strongest GWAS signal in European ancestry, SNP rs7086624 (*Z* score = −3.68, *p* value = 2 × 10^−4^), exhibits negligible association in East Asian ancestry (*Z* score = −0.02, *p* value = 0.037). Conversely, the top signal in East Asian ancestry, SNP rs7898735 (*Z* score = −12.66, *p* value = 1.02 × 10^−36^), shows non-significant association in European ancestry (*Z* score = −2.82, *p* value = 0.005). Additionally, the BH-adjusted *p* value for the composite null hypothesis of heritability testing is 0.09, suggesting limited evidence for non-zero heritability in this region across both ancestries. Collectively, these results support the conclusion that this region does not exhibit genetic correlation (Figure S18). Consistent with this, Logica estimated a non-significant genetic correlation (*γ*_*g*_ = 0.57, *p* value = 0.34). In contrast, both XPASS and Popcorn identified significant genetic correlation in this region (XPASS: *γ*_*g*_ = 1, *p* value ≈ 0; Popcorn: *γ*_*g*_ = 1.16, *p* value = 2 × 10^−11^), echoing the simulation results in which some of these methods may incorrectly identify genetic correlation in regions lacking true shared signals.

In real data analysis, computational cost mirrored those observed in simulations. Logica averaged 4.95 min per region, with the initial heritable region screening averaging 1.61 min, compared to 0.01 min and 0.08 min per region for XPASS and Popcorn, respectively (Figure S21). Regarding peak memory usage, Logica averaged 1,114 MB per region, whereas XPASS and Popcorn averaged 210 MB and 174 MB, respectively. The maximum peak memory recorded for Logica was 6,084 MB for a region containing 6,350 SNPs (Figure S22).

## Discussion

We have presented Logica, a method for local genetic correlation estimation across ancestries. Logica utilizes a bivariate linear mixed model to explicitly account for diverse LD structures across ancestries, performs inference within a likelihood framework, and relies on a parameter expansion EM algorithm for scalable optimization. As a result, Logica provides more accurate local genetic correlation estimates, effectively detects genomic regions with significant local genetic correlation across a range of scenarios, and remains computationally efficient. As global biobanks continue to expand in size and diversity, integrating GWAS data from historically under-represented populations through the Logica framework is expected to enhance our understanding of shared genetic underpinning of complex traits and disease across groups, ultimately helping to extend the benefits of GWAS to these under-represented populations.

Several local genetic correlation methods have been developed for analyzing correlations across traits within a single ancestry, such as SUPERGNOVA and LAVA.^15^^,^^41^ However, these methods typically rely on an LD reference panel from a single ancestry and therefore do not account for differences in LD structure across ancestries. As a result, they are primarily suitable for cross-trait correlation estimation within a single ancestry. If one were to use these existing methods in a cross-ancestry setting, an ad hoc approach would be to apply them using the LD reference panel from only one ancestry. This would lead to a mismatch in LD patterns for other ancestries, likely resulting in biased local heritability estimates and, consequently, biased estimates of local genetic correlations. We provide a simulation-based illustration of this limitation (detailed in Note S3).

In our study, European and East Asian analyses were based on LD reference panels of different sample sizes: 50,000 individuals from the UKB for Europeans and 504 individuals from 1000G for East Asians. This difference reflected practical considerations, as larger European reference panels are more readily available than those for other ancestries. To further evaluate the impact of this choice, we repeated the analyses using 503 European individuals from 1000G, which ensured balanced panel sizes in simulation and the use of external panels for both ancestries in real data. Across both settings, Logica consistently outperformed comparison methods, and results were similar regardless of the LD reference panel used (details in Note S4).

While Logica offers a powerful and scalable framework for local genetic correlation inference across ancestries, it is not without limitations. First, genetic correlation analyses across ancestries focus only on common SNPs, neglecting population-specific variants. Our simulations show that, when population-specific variants contribute to local heritability, both Logica and compared methods yield upwardly biased correlation estimates (Note S5). There remains no established consensus on defining genetic correlation that fully accounts for these variants. One potential methodological extension is partial correlation analysis. Specifically, this approach could involve separately estimating local heritability (including both common and ancestry-specific SNPs) and genetic covariance (using only common SNPs), subsequently defining local genetic correlation as the ratio of genetic covariance to the geometric mean of local heritability. Second, Logica requires storing LD matrices during computation, which can result in high memory demands. A potential workaround is to approximate each LD matrix as the sum of a low-rank matrix and a banded matrix, as suggested by Li et al.,^16^ or to leverage sparse LD matrix following Li et al.,^42^ both of which can substantially reduce memory usage. However, the impact of such approximation on estimation accuracy remains to be evaluated. Third, the current implementation of Logica examines genetic correlation between two ancestries at a time. While Logica can be extended to accommodate more than two ancestries, its computational complexity scales cubically with both the number of ancestries and the number of SNPs. Consequently, applying Logica to more than two ancestries is expected to increase computational burden. Finally, our current practical approach of hypothesis testing remains a two-step procedure: first separately testing for heritability in both ancestries and subsequently testing genetic covariance. A fully joint testing framework, while methodologically desirable, requires substantial further methodological research and innovation. Specifically, we are interested in identifying regions where $h_{1k}^{2}\neq 0\;\text{and}\;h_{2k}^{2}\neq 0\;\text{and}\;\rho_{gk}=0$, which involves three distinct parameters $h_{1k}^{2},h_{2k}^{2}$, and *ρ*_*gk*_. By definition, any configuration in which at least one heritability is zero necessarily implies zero genetic covariance, as the corresponding genetic effect has zero variance and therefore cannot covary with the other. This restriction reduces the full 8-binary-parameter space to five valid scenarios, a–e: (a) $h_{1k}^{2}=0\;\text{and}\;h_{2k}^{2}\neq 0\;\text{and}\;\rho_{gk}=0$; (b) $h_{1k}^{2}\neq 0\;\text{and}\;h_{2k}^{2}=0\;\text{and}\;\rho_{gk}=0$; (c) $h_{1k}^{2}=0\;\text{and}\;h_{2k}^{2}=0\;\text{and}\;\rho_{gk}=0$; (d) $h_{1k}^{2}\neq 0\;\text{and}\;h_{2k}^{2}\neq 0\;\text{and}\;\rho_{gk}=0$; and (e) $h_{1k}^{2}\neq 0\;\text{and}\;h_{2k}^{2}\neq 0\;\text{and}\;\rho_{gk}\neq 0$. Given that scenario (d) is our desired alternative, the composite null hypothesis is

$$H_{0}:a\cup b\cup c\cup e\text{.}$$

This composite null hypothesis is challenging because it encompasses multiple distinct scenarios and internal conditions involving non-zero genetic correlation (scenario d). Consequently, constructing an appropriate joint test statistic is non-trivial, as it requires explicitly estimating the mixture proportions for these null scenarios and accurately characterizing the null distribution. Additionally, accurately controlling the type I error rate is significantly complicated by this mixture.

There are several important potential future extensions of Logica. For example, Logica could be extended to estimate genetic correlation across all genomic regions jointly by incorporating a global local shrinkage prior, as suggested by Ge et al.,^43^ which may further improve estimation accuracy. Additionally, within each genomic region, Logica can be extended to better capture local genetic architecture by modeling both large-effect and infinitesimal-effect SNPs, following Zhou et al.^44^ Furthermore, incorporating SNP functional annotations into Logica could further enhance its estimation accuracy.^19^^,^^22^ In addition, we extended Logica to admixed populations by explicitly modeling local ancestry dosage, which allows for the estimation of local genetic correlation in admixed individuals. In simulations, we show that this extension provides unbiased estimates of local genetic correlation across local ancestry dosages (detailed in Note S6). Finally, quantifying the contribution of common and rare variants to genetic correlation can provide deeper insights into shared genetic architectures across ancestries. Several methods, such as GREML-LDMS and BOLT-REML, have been developed to quantify heritability contributions based on SNP MAF and LD.^45^^,^^46^ Logica could be further extended to estimate genetic correlation using SNPs within specific MAF bins, allowing for the characterization of how SNPs with different MAFs contribute to genetic correlation. Such extensions, however, remain challenging, since many rare variants are population specific, complicating a clear definition of genetic correlation in this context.^6^

## Data and code availability

The R package developed in this study, Logica (v.1.0), is publicly available at https://xiangzhou.github.io/software/.

## Acknowledgments

This study was supported by the 10.13039/100000002National Institutes of Health (NIH) grants R01HG009124 and R01GM144960 (to X.Z.). The funders had no role in study design, data collection and analysis, decision to publish, or preparation of the manuscript. This study has been conducted using the UK Biobank resource under application number 30186. UK Biobank was established by the Wellcome Trust medical charity, Medical Research Council, Department of Health, Scottish Government, and the Northwest Regional Development Agency. It has also had funding from the Welsh Assembly Government, 10.13039/501100000274British Heart Foundation, and 10.13039/501100000361Diabetes UK. Our study complies with all pertinent ethical regulations. This study was approved by the University of Michigan Institutional Review Board (HUM00156494).

## Declaration of interests

The authors declare no competing interests.

## Web resources

Biobank Japan Project GWAS summary statistics, https://pheweb.jp/downloads
Korean Biobank Project GWAS summary statistics, https://zenodo.org/records/7042518
ma-focus, https://github.com/mancusolab/ma-focus/tree/master/pyfocus/data/ld_blocks
Neale lab GWAS summary statistics, https://docs.google.com/spreadsheets/d/1kvPoupSzsSFBNSztMzl04xMoSC3Kcx3CrjVf4yBmESU/edit?gid=227859291#gid=227859291
Taiwan Biobank GWAS summary statistics, http://ftp.ebi.ac.uk/pub/databases/gwas/summary_statistics/

## Supplemental information

Document S1. Figures S1–S36, Tables S1–S3, and Notes S1–S6

Document S2. Article plus supplemental information

## References

[1] Taliun D., Harris D.N., Kessler M.D., Carlson J., Szpiech Z.A., Torres R., Taliun S.A.G., Corvelo A., Gogarten S.M., Kang H.M. (2021). Sequencing of 53,831 diverse genomes from the NHLBI TOPMed Program. Nature.

[2] All of Us Research Program Genomics Investigators (2024). Genomic data in the All of Us Research Program. Nature.

[3] Nagai A., Hirata M., Kamatani Y., Muto K., Matsuda K., Kiyohara Y., Ninomiya T., Tamakoshi A., Yamagata Z., Mushiroda T. (2017). Overview of the BioBank Japan Project: Study design and profile. J. Epidemiol..

[4] Sudlow C., Gallacher J., Allen N., Beral V., Burton P., Danesh J., Downey P., Elliott P., Green J., Landray M. (2015). UK Biobank: An Open Access Resource for Identifying the Causes of a Wide Range of Complex Diseases of Middle and Old Age. PLoS Med..

[5] Bycroft C., Freeman C., Petkova D., Band G., Elliott L.T., Sharp K., Motyer A., Vukcevic D., Delaneau O., O’Connell J. (2018). The UK Biobank resource with deep phenotyping and genomic data. Nature.

[6] Brown B.C., Ye C.J., Price A.L., Zaitlen N., Asian Genetic Epidemiology Network Type 2 Diabetes Consortium (2016). Transethnic Genetic-Correlation Estimates from Summary Statistics. Am. J. Hum. Genet..

[7] Cai M., Xiao J., Zhang S., Wan X., Zhao H., Chen G., Yang C. (2021). A unified framework for cross-population trait prediction by leveraging the genetic correlation of polygenic traits. Am. J. Hum. Genet..

[8] Shi H., Gazal S., Kanai M., Koch E.M., Schoech A.P., Siewert K.M., Kim S.S., Luo Y., Amariuta T., Huang H. (2021). Population-specific causal disease effect sizes in functionally important regions impacted by selection. Nat. Commun..

[9] Miao J., Guo H., Song G., Zhao Z., Hou L., Lu Q. (2023). Quantifying portable genetic effects and improving cross-ancestry genetic prediction with GWAS summary statistics. Nat. Commun..

[10] LaPierre N., Taraszka K., Huang H., He R., Hormozdiari F., Eskin E. (2021). Identifying causal variants by fine mapping across multiple studies. PLoS Genet..

[11] Kichaev G., Roytman M., Johnson R., Eskin E., Lindström S., Kraft P., Pasaniuc B. (2017). Improved methods for multi-trait fine mapping of pleiotropic risk loci. Bioinformatics.

[12] Yuan K., Longchamps R.J., Pardiñas A.F., Yu M., Chen T.-T., Lin S.-C., Chen Y., Lam M., Liu R., Xia Y. (2023). Fine-mapping across diverse ancestries drives the discovery of putative causal variants underlying human complex traits and diseases. medRxiv.

[13] Gao B., Zhou X. (2024). MESuSiE enables scalable and powerful multi-ancestry fine-mapping of causal variants in genome-wide association studies. Nat. Genet..

[14] Shi H., Mancuso N., Spendlove S., Pasaniuc B. (2017). Local Genetic Correlation Gives Insights into the Shared Genetic Architecture of Complex Traits. Am. J. Hum. Genet..

[15] Zhang Y., Lu Q., Ye Y., Huang K., Liu W., Wu Y., Zhong X., Li B., Yu Z., Travers B.G. (2021). SUPERGNOVA: local genetic correlation analysis reveals heterogeneous etiologic sharing of complex traits. Genome Biol..

[16] Li H., Mazumder R., Lin X. (2023). Accurate and efficient estimation of local heritability using summary statistics and the linkage disequilibrium matrix. Nat. Commun..

[17] Lu Z., Gopalan S., Yuan D., Conti D.V., Pasaniuc B., Gusev A., Mancuso N. (2022). Multi-ancestry fine-mapping improves precision to identify causal genes in transcriptome-wide association studies. Am. J. Hum. Genet..

[18] Shi H., Burch K.S., Johnson R., Freund M.K., Kichaev G., Mancuso N., Manuel A.M., Dong N., Pasaniuc B. (2020). Localizing Components of Shared Transethnic Genetic Architecture of Complex Traits from GWAS Summary Data. Am. J. Hum. Genet..

[19] Zhou X. (2017). A unified framework for variance component estimation with summary statistics in genome-wide association studies. Ann. Appl. Stat..

[20] Gao B., Yang C., Liu J., Zhou X. (2021). Accurate genetic and environmental covariance estimation with composite likelihood in genome-wide association studies. PLoS Genet..

[21] Bulik-Sullivan B.K., Loh P.R., Finucane H.K., Ripke S., Yang J., Patterson N., Daly M.J., Price A.L., Neale B.M., Schizophrenia Working Group of the Psychiatric Genomics Consortium (2015). LD score regression distinguishes confounding from polygenicity in genome-wide association studies. Nat. Genet..

[22] Lu Q., Li B., Ou D., Erlendsdottir M., Powles R.L., Jiang T., Hu Y., Chang D., Jin C., Dai W. (2017). A Powerful Approach to Estimating Annotation-Stratified Genetic Covariance via GWAS Summary Statistics. Am. J. Hum. Genet..

[23] Liu C., Rubin D.B., Ying Nian W.U. (1998). Parameter expansion to accelerate EM: the PX-EM algorithm. Biometrika.

[24] Dai J.Y., Stanford J.L., LeBlanc M. (2022). A Multiple-Testing Procedure for High-Dimensional Mediation Hypotheses. J. Am. Stat. Assoc..

[25] Storey J.D. (2002). A direct approach to false discovery rates. J. R. Stat. Soc. Series B Stat. Methodol..

[26] Du J., Zhou X., Clark-Boucher D., Hao W., Liu Y., Smith J.A., Mukherjee B. (2023). Methods for large-scale single mediator hypothesis testing: Possible choices and comparisons. Genet. Epidemiol..

[27] Yang J., Lee S.H., Goddard M.E., Visscher P.M. (2011). GCTA: A tool for genome-wide complex trait analysis. Am. J. Hum. Genet..

[28] Song S., Jiang W., Zhang Y., Hou L., Zhao H. (2022). Leveraging LD eigenvalue regression to improve the estimation of SNP heritability and confounding inflation. Am. J. Hum. Genet..

[29] Auton A., Abecasis G.R., Altshuler D.M., Durbin R.M., Bentley D.R., Chakravarti A., Clark A.G., Donnelly P., Eichler E.E., Flicek P. (2015). A global reference for human genetic variation. Nature.

[30] Wang L., Gao B., Fan Y., Xue F., Zhou X. (2021). Mendelian randomization under the omnigenic architecture. Brief. Bioinform..

[31] Zou Y., Carbonetto P., Wang G., Stephens M. (2022). Fine-mapping from summary data with the “Sum of Single Effects” model. PLoS Genet..

[32] Weissbrod O., Hormozdiari F., Benner C., Cui R., Ulirsch J., Gazal S., Schoech A.P., van de Geijn B., Reshef Y., Márquez-Luna C. (2020). Functionally informed fine-mapping and polygenic localization of complex trait heritability. Nat. Genet..

[33] Sakaue S., Kanai M., Tanigawa Y., Karjalainen J., Kurki M., Koshiba S., Narita A., Konuma T., Yamamoto K., Akiyama M. (2021). A cross-population atlas of genetic associations for 220 human phenotypes. Nat. Genet..

[34] Kim Y.J., Moon S., Hwang M.Y., Han S., Jang H.M., Kong J., Shin D.M., Yoon K., Kim S.M., Lee J.E. (2022). The contribution of common and rare genetic variants to variation in metabolic traits in 288,137 East Asians. Nat. Commun..

[35] Feng Y.C.A., Chen C.Y., Chen T.T., Kuo P.H., Hsu Y.H., Yang H.I., Chen W.J., Su M.W., Chu H.W., Shen C.Y. (2022). Taiwan Biobank: A rich biomedical research database of the Taiwanese population. Cell Genom..

[36] Willer C.J., Li Y., Abecasis G.R. (2010). METAL: Fast and efficient meta-analysis of genomewide association scans. Bioinformatics.

[37] McLaren W., Gil L., Hunt S.E., Riat H.S., Ritchie G.R.S., Thormann A., Flicek P., Cunningham F. (2016). The Ensembl Variant Effect Predictor. Genome Biol..

[38] Lonsdale J., Thomas J., Salvatore M., Phillips R., Lo E., Shad S., Hasz R., Walters G., Garcia F., Young N. (2013). The Genotype-Tissue Expression (GTEx) project. Nat. Genet..

[39] Eichner J.E., Dunn S.T., Perveen G., Thompson D.M., Stewart K.E., Stroehla B.C. (2002). Apolipoprotein E polymorphism and cardiovascular disease: A HuGE review. Am. J. Epidemiol..

[40] Shivdasani R.A., Rosenblatt M.F., Zucker-Franklin D., Jackson C.W., Hunt P., Saris C.J., Orkin S.H. (1995). Transcription factor NF-E2 is required for platelet formation independent of the actions of thrombopoeitin/MGDF in megakaryocyte development. Cell.

[41] Werme J., van der Sluis S., Posthuma D., de Leeuw C.A. (2022). An integrated framework for local genetic correlation analysis. Nat. Genet..

[42] Li X., Quick C., Zhou H., Gaynor S.M., Liu Y., Chen H., Selvaraj M.S., Sun R., Dey R., Arnett D.K. (2023). Powerful, scalable and resource-efficient meta-analysis of rare variant associations in large whole genome sequencing studies. Nat. Genet..

[43] Ge T., Chen C.Y., Ni Y., Feng Y.C.A., Smoller J.W. (2019). Polygenic prediction via Bayesian regression and continuous shrinkage priors. Nat. Commun..

[44] Zhou X., Carbonetto P., Stephens M. (2013). Polygenic Modeling with Bayesian Sparse Linear Mixed Models. PLoS Genet..

[45] Yang J., Bakshi A., Zhu Z., Hemani G., Vinkhuyzen A.A.E., Lee S.H., Robinson M.R., Perry J.R.B., Nolte I.M., Van Vliet-Ostaptchouk J.V. (2015). Genetic variance estimation with imputed variants finds negligible missing heritability for human height and body mass index. Nat. Genet..

[46] Loh P.R., Bhatia G., Gusev A., Finucane H.K., Bulik-Sullivan B.K., Pollack S.J., de Candia T.R., Lee S.H., Wray N.R., Schizophrenia Working Group of Psychiatric Genomics Consortium (2015). Contrasting genetic architectures of schizophrenia and other complex diseases using fast variance-components analysis. Nat. Genet..

