# Supplementary material for: Logica: A likelihood framework for cross-ancestry local genetic correlation estimation using summary statistics
Source: Am J Hum Genet. 2025 Oct 23;112(11):2789–804. doi: 10.1016/j.ajhg.2025.10.001 (PMC12739644; doi:10.1016/j.ajhg.2025.10.001)
Supplement: Document S1. Figures S1–S36, Tables S1–S3, and Notes S1–S6 [file mmc1.pdf]

The American Journal of Human Genetics, Volume *112*

Supplemental information

Logica: A likelihood framework for cross-ancestry local genetic correlation estimation using summary statistics

Boran Gao, Zheng Li, and Xiang Zhou

Supplemental Figure

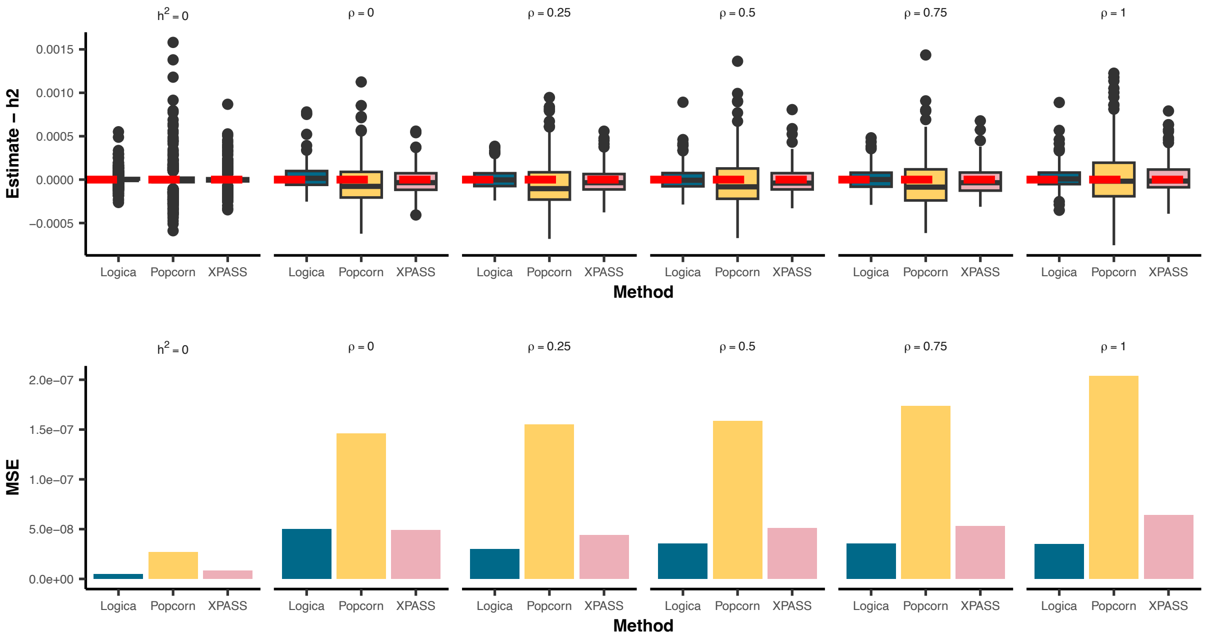

Figure S1: Comparison of local heritability estimates of different methods in baseline simulation setting. Results are shown for the baseline simulation setting with n = 1,368 independent regions across the genome. The simulated sample size for both ancestries is set to 300,000, and in-sample LD matrices are used for model fitting. Among these regions, 40% have zero heritability in both ancestries, 20% exhibit non-zero heritability in only one ancestry with equal probability, and 40% exhibit non-zero heritability in both ancestries. The heritability of these regions is set to either 3×10^{-5} or 5×10^{-5} with equal probability. For regions exhibiting non-zero heritability in both ancestries, genetic correlation is set to 0, 0.25, 0.5, 0.75, or 1 with equal probability. From left to right, columns represent scenarios with zero heritability in at least one ancestry, and scenarios with non-zero heritability in both ancestries, each varying by genetic correlation (0, 0.25, 0.5, 0.75, 1). We compare the performance of Logica (blue) against Popcorn (yellow) and XPASS (pink). The top panel shows boxplots of estimated heritability – true heritability. The bottom panel displays bar plots of mean squared error (MSE) of heritability estimates across different true local genetic correlation values.

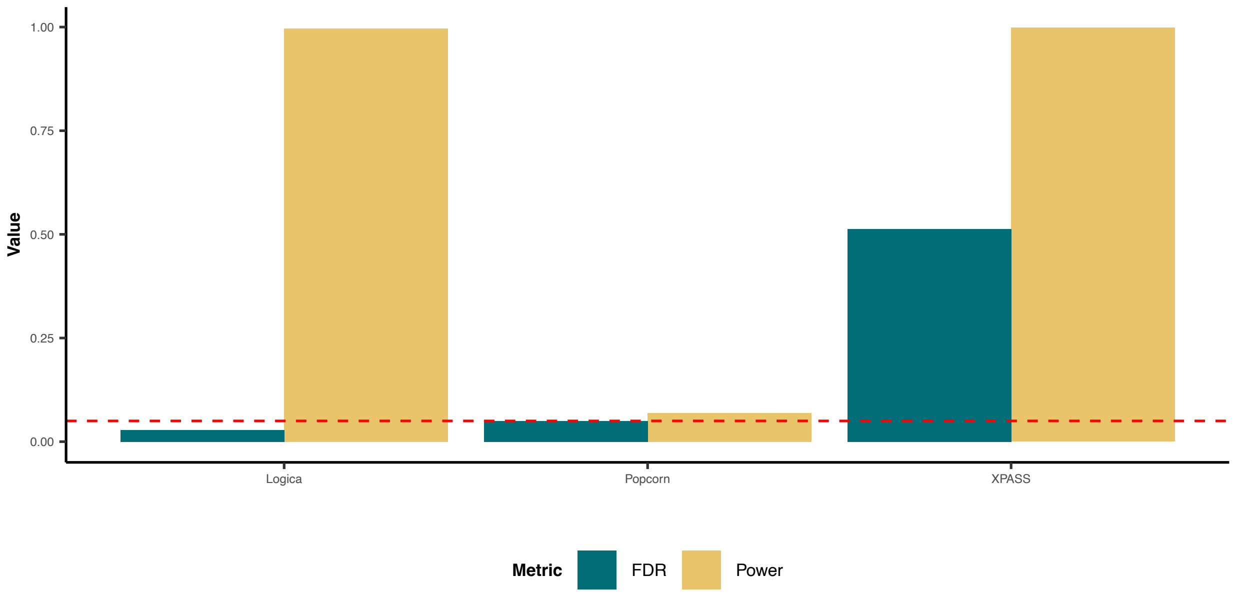

Figure S2: Comparison of FDR and Power among methods for detecting regions with non-zero heritability in both ancestries in the baseline setting. We declare a region as with non-zero heritability in both ancestries if its Benjamini-Hochberg (BH)-adjusted P-value is below a specified threshold. Power is defined as the number of detected signals divided by the total number of genetically correlated regions. False Discovery Rate (FDR) is defined as the number of falsely detected regions divided by the total number of detected genetically correlated regions. FDR (dark green) and power (yellow) are compared among methods based on a BH-adjusted P-value threshold of 0.05. The dashed red line indicates the nominal FDR threshold of 0.05.

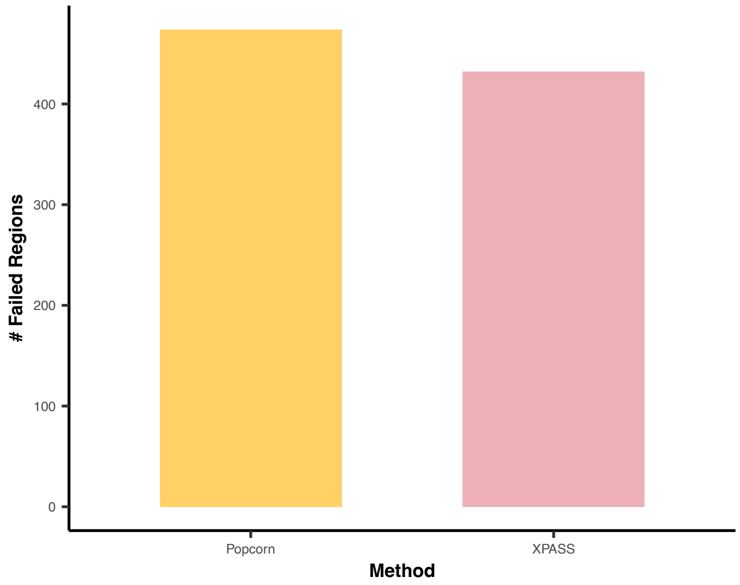

Figure S3: Number of genomic regions for which Popcorn and XPASS failed to provide local genetic correlation estimates in the baseline simulation setting.

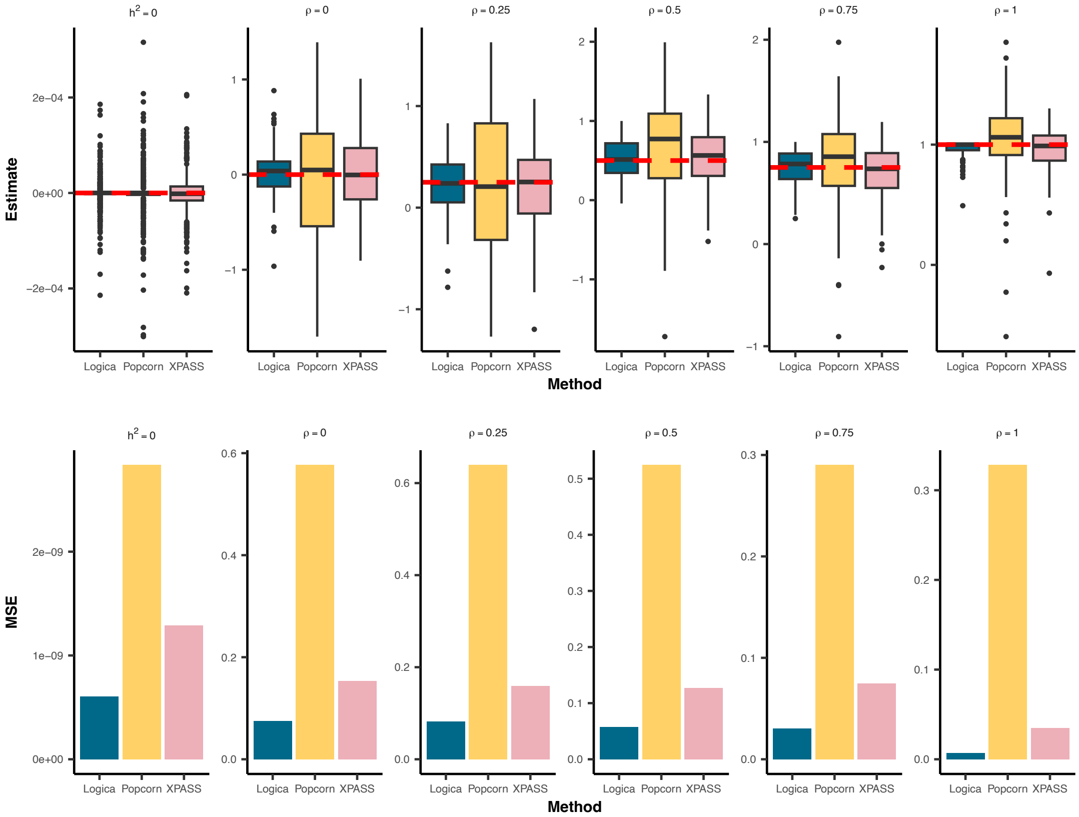

Figure S4: Comparison of local genetic correlation estimation methods in setting when *50% of SNPs have non-zero effects*. Results are shown for the simulation setting with n = 1,368 independent regions across the genome. The simulated sample size for both ancestries is set to 300,000, and in-sample LD matrices are used for model fitting. Among these regions, 40% have zero heritability in both ancestries, 20% exhibit non-zero heritability in only one ancestry with equal probability, and 40% exhibit non-zero heritability in both ancestries. The heritability of these regions is set to either 3×10^{-5} or 5×10^{-5} with equal probability. For regions exhibiting non-zero heritability in both ancestries, genetic correlation is set to 0, 0.25, 0.5, 0.75, or 1 with equal probability. From left to right, columns represent scenarios with zero heritability in at least one ancestry, and scenarios with non-zero heritability in both ancestries, each varying by genetic correlation (0, 0.25, 0.5, 0.75, 1). We compare the performance of Logica (blue) against Popcorn (yellow) and XPASS (pink). The top panel shows boxplots of estimated local genetic correlations. The bottom panel displays bar plots of mean squared error (MSE) across different true local genetic correlation values.

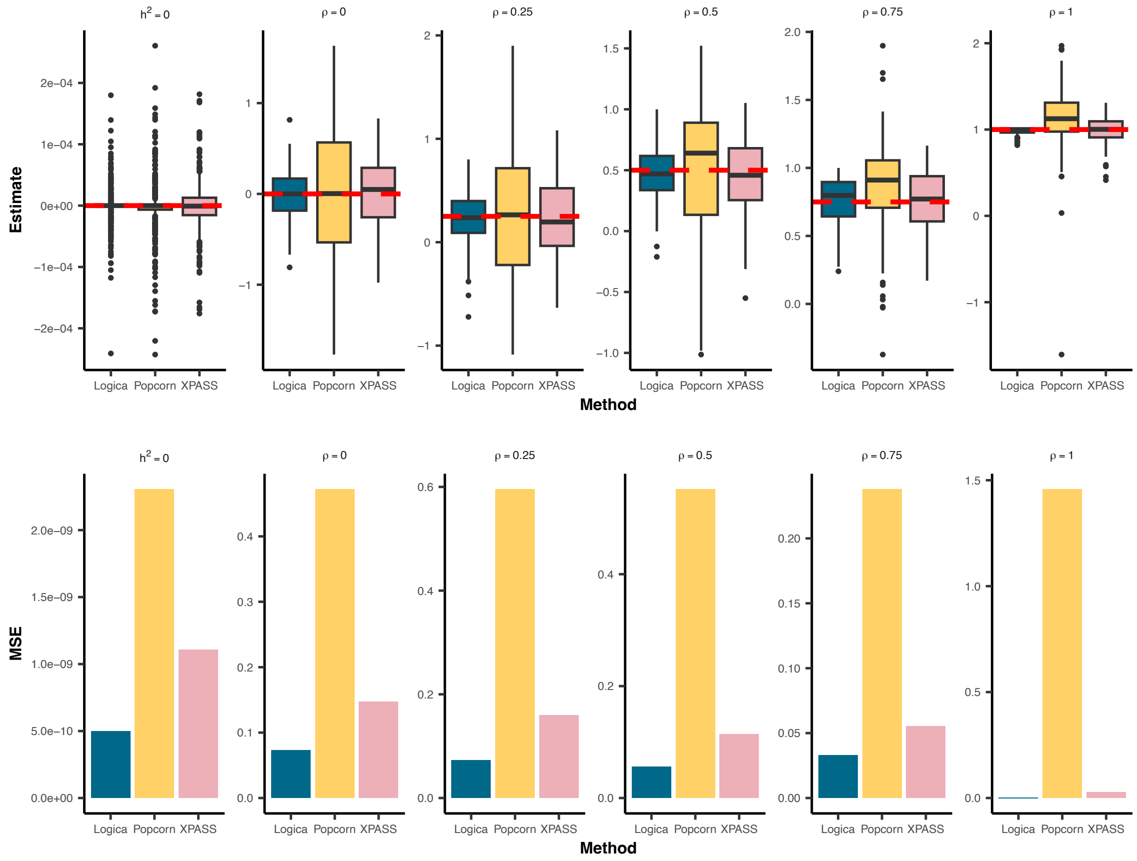

Figure S5: Comparison of local genetic correlation estimation methods in setting when *10% of SNPs have non-zero effects*. Results are shown for the simulation setting with $n = 1{,}368$ independent regions across the genome. The simulated sample size for both ancestries is set to 300,000, and in-sample LD matrices are used for model fitting. Among these regions, 40% have zero heritability in both ancestries, 20% exhibit non-zero heritability in only one ancestry with equal probability, and 40% exhibit non-zero heritability in both ancestries. The heritability of these regions is set to either 3×10^{-5} or 5×10^{-5} with equal probability. For regions exhibiting non-zero heritability in both ancestries, genetic correlation is set to 0, 0.25, 0.5, 0.75, or 1 with equal probability. From left to right, columns represent scenarios with zero heritability in at least one ancestry, and scenarios with non-zero heritability in both ancestries, each varying by genetic correlation (0, 0.25, 0.5, 0.75, 1). We compare the performance of Logica (blue) against Popcorn (yellow) and XPASS (pink). The top panel shows boxplots of estimated local genetic correlations. The bottom panel displays bar plots of mean squared error (MSE) across different true local genetic correlation values.

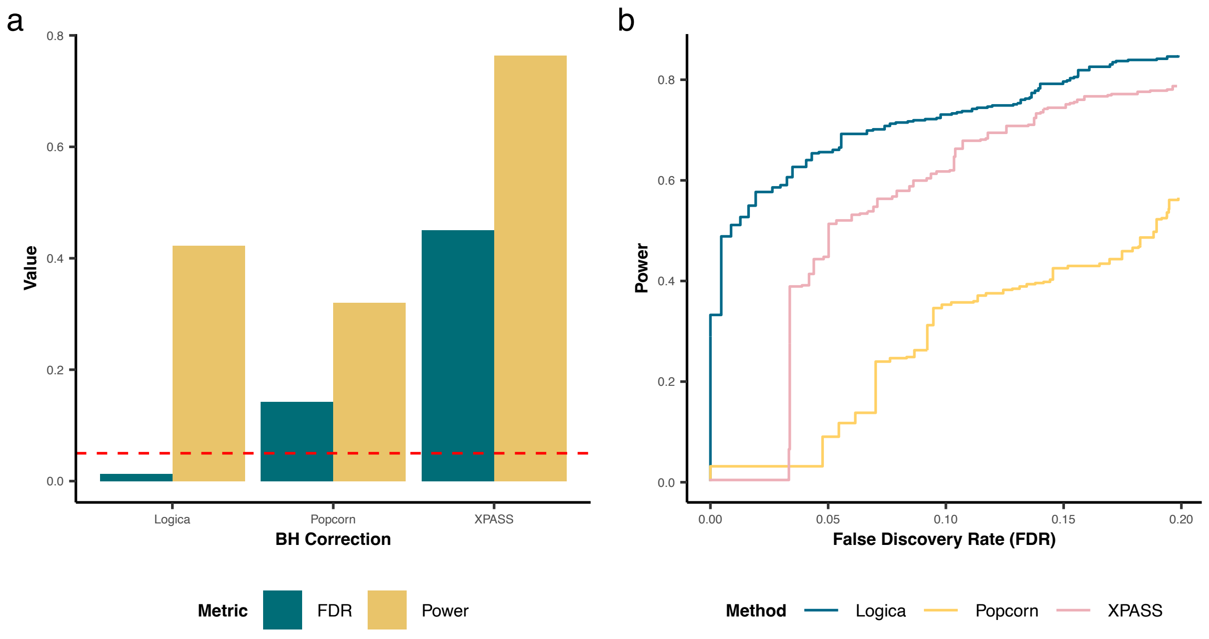

Figure S6: Comparison of FDR and Power among methods for detecting local genetically correlated regions across ancestries in the setting when *50% of SNPs have non-zero effects*. We declare a region as genetically correlated if its Benjamini-Hochberg (BH)-adjusted P-value is below a specified threshold. Power is defined as the number of detected signals divided by the total number of genetically correlated regions. False Discovery Rate (FDR) is defined as the number of falsely detected regions divided by the total number of detected genetically correlated regions. Left panel: FDR (dark green) and power (yellow) are compared among methods based on a BH-adjusted P-value threshold of 0.05. The dashed red line indicates the nominal FDR threshold of 0.05. Right panel: To enable fair comparison, an FDR-power plot is presented comparing the power of Logica (blue), Popcorn (yellow), and XPASS (pink) across different FDR levels.

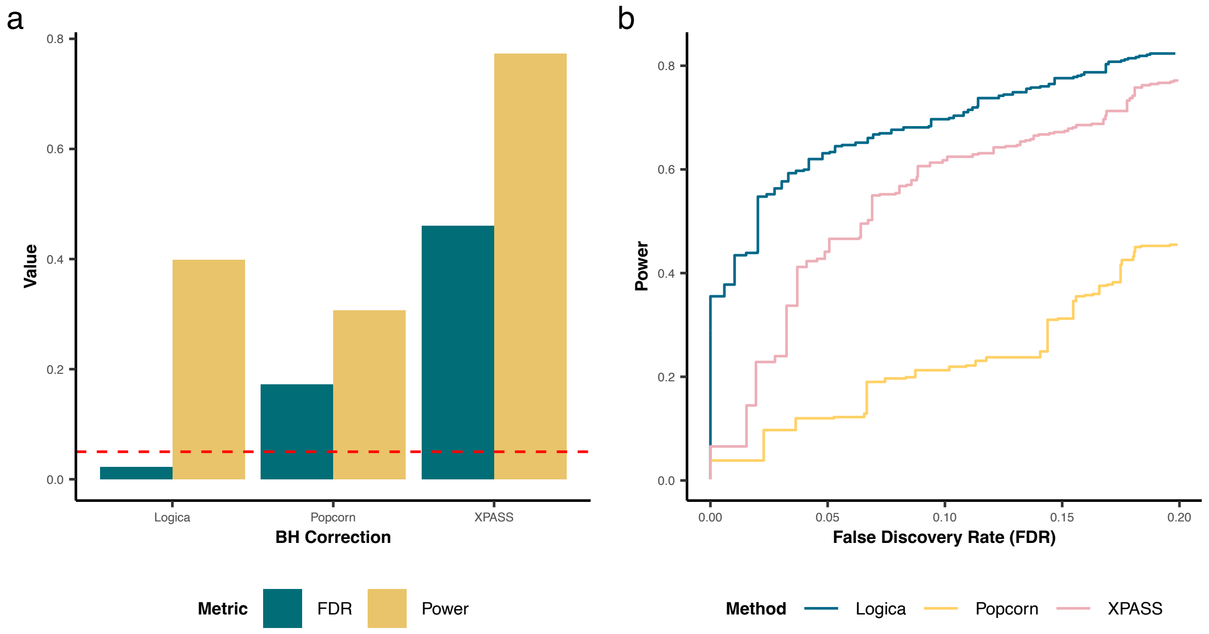

Figure S7: Comparison of FDR and Power among methods for detecting local genetically correlated regions across ancestries in the setting when *10% of SNPs have non-zero effects*. We declare a region as genetically correlated if its Benjamini-Hochberg (BH)-adjusted P-value is below a specified threshold. Power is defined as the number of detected signals divided by the total number of genetically correlated regions. False Discovery Rate (FDR) is defined as the number of falsely detected regions divided by the total number of detected genetically correlated regions. Left panel: FDR (dark green) and power (yellow) are compared among methods based on a BH-adjusted P-value threshold of 0.05. The dashed red line indicates the nominal FDR threshold of 0.05. Right panel: To enable fair comparison, an FDR-power plot is presented comparing the power of Logica (blue), Popcorn (yellow), and XPASS (pink) across different FDR levels.

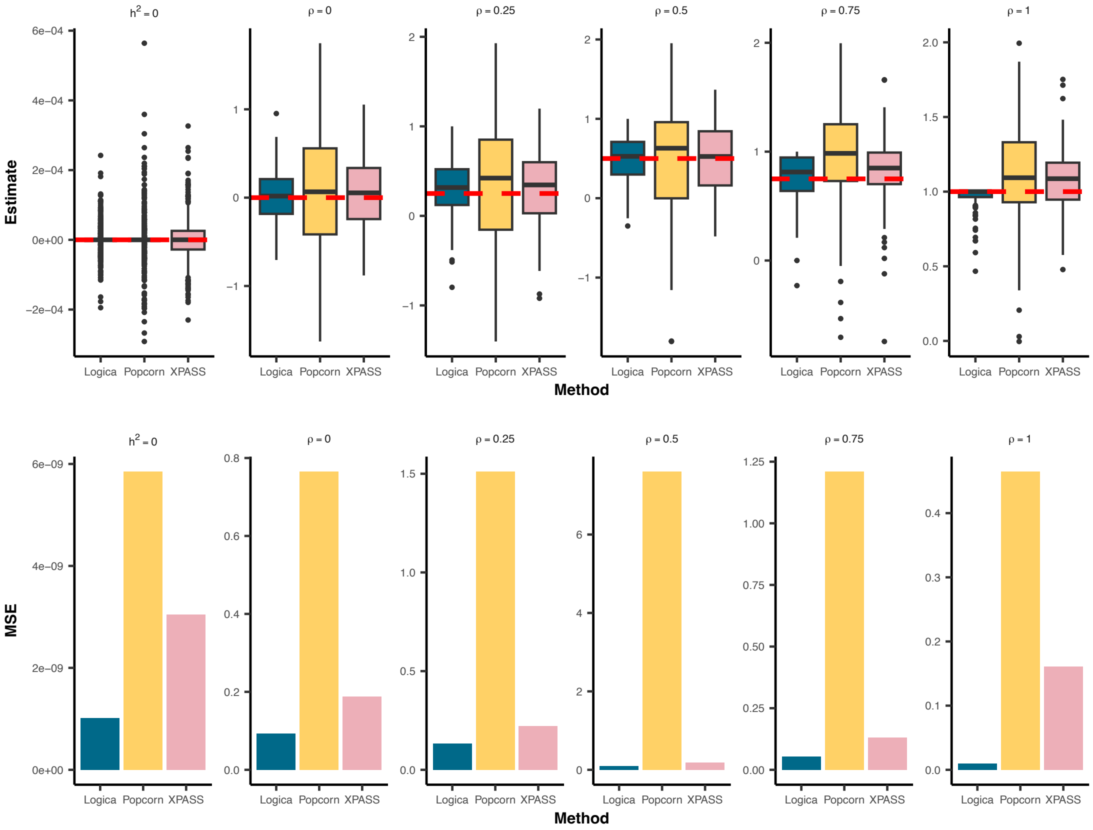

Figure S8: Comparison of local genetic correlation estimation methods in setting with *unbalanced sample size across ancestries*. We simulated data across 1,368 independent genomic regions, with sample sizes set at 300,000 for European ancestry and 100,000 for East-Asian ancestry. In-sample LD matrices were utilized for model fitting. Among these regions, 40% have zero heritability in both ancestries, 20% exhibit non-zero heritability in only one ancestry with equal probability, and 40% exhibit non-zero heritability in both ancestries. The heritability of these regions is set to either 3×10^{-5} or 5×10^{-5} with equal probability. For regions exhibiting non-zero heritability in both ancestries, genetic correlation is set to 0, 0.25, 0.5, 0.75, or 1 with equal probability. From left to right, columns represent scenarios with zero heritability in at least one ancestry, and scenarios with non-zero heritability in both ancestries, each varying by genetic correlation (0, 0.25, 0.5, 0.75, 1). We compare the performance of Logica (blue) against Popcorn (yellow) and XPASS (pink). The top panel shows boxplots of estimated local genetic correlations. The bottom panel displays bar plots of mean squared error (MSE) across different true local genetic correlation values.

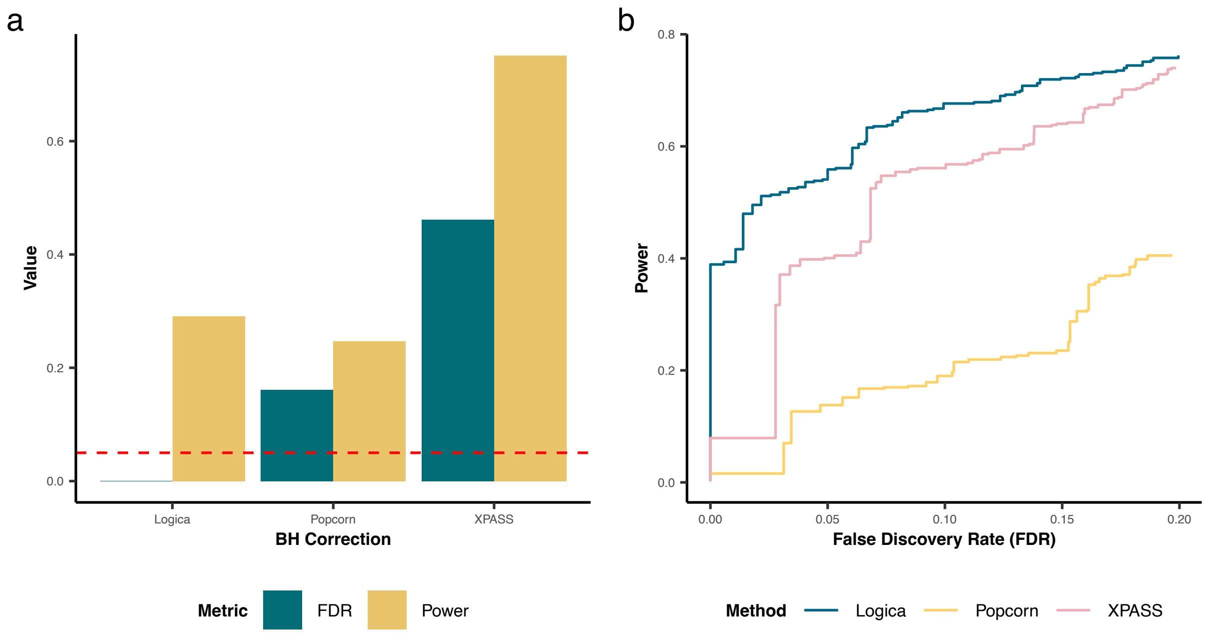

Figure S9: Comparison of FDR and Power among methods for detecting local genetically correlated regions across ancestries in the in setting with *unbalanced sample size across ancestries*. We declare a region as genetically correlated if its Benjamini-Hochberg (BH)-adjusted P-value is below a specified threshold. Power is defined as the number of detected signals divided by the total number of genetically correlated regions. False Discovery Rate (FDR) is defined as the number of falsely detected regions divided by the total number of detected genetically correlated regions. Left panel: FDR (dark green) and power (yellow) are compared among methods based on a BH-adjusted P-value threshold of 0.05. The dashed red line indicates the nominal FDR threshold of 0.05. Right panel: To enable fair comparison, an FDR-power plot is presented comparing the power of Logica (blue), Popcorn (yellow), and XPASS (pink) across different FDR levels.

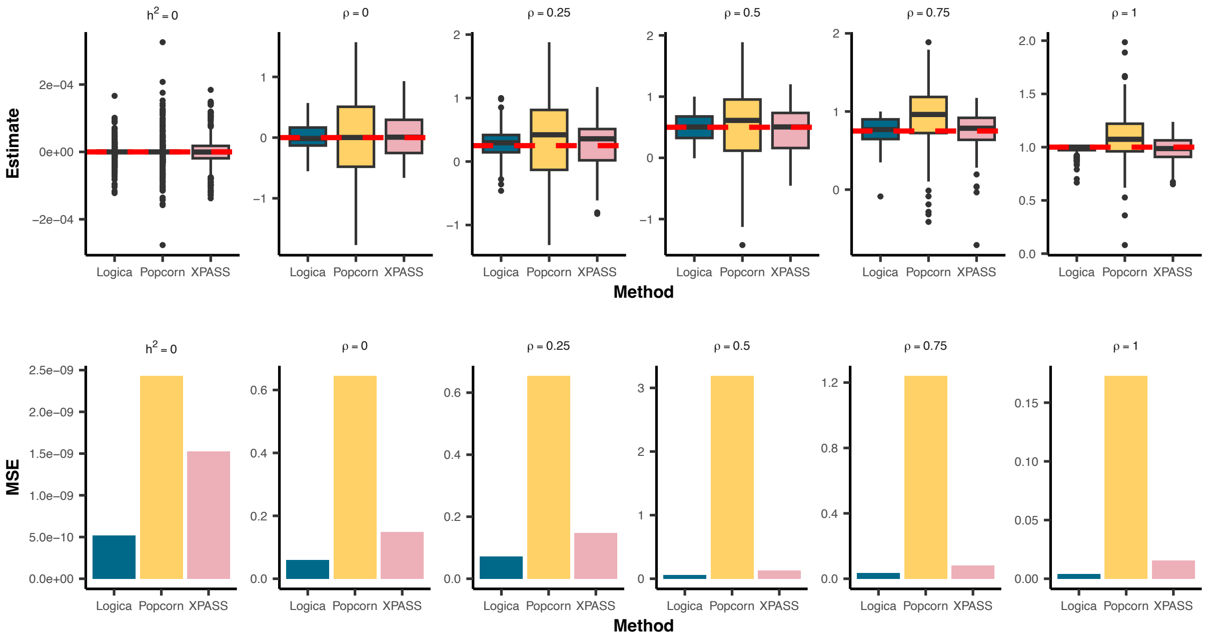

Figure S10: Comparison of local genetic correlation estimation methods in setting with *population stratification*. Results are shown for the baseline simulation setting with n = 1,368 independent regions across the genome. The simulated sample size for both ancestries is set to 300,000, and in-sample LD matrices are used for model fitting. Among these regions, 40% have zero heritability in both ancestries, 20% exhibit non-zero heritability in only one ancestry with equal probability, and 40% exhibit non-zero heritability in both ancestries. The heritability of these regions is set to either 3×10^{-5} or 5×10^{-5} with equal probability. For regions exhibiting non-zero heritability in both ancestries, genetic correlation is set to 0, 0.25, 0.5, 0.75, or 1 with equal probability. From left to right, columns represent scenarios with zero heritability in at least one ancestry, and scenarios with non-zero heritability in both ancestries, each varying by genetic correlation (0, 0.25, 0.5, 0.75, 1). We compare the performance of Logica (blue) against Popcorn (yellow) and XPASS (pink). The top panel shows boxplots of estimated local genetic correlations. The bottom panel displays bar plots of mean squared error (MSE) across different true local genetic correlation values.

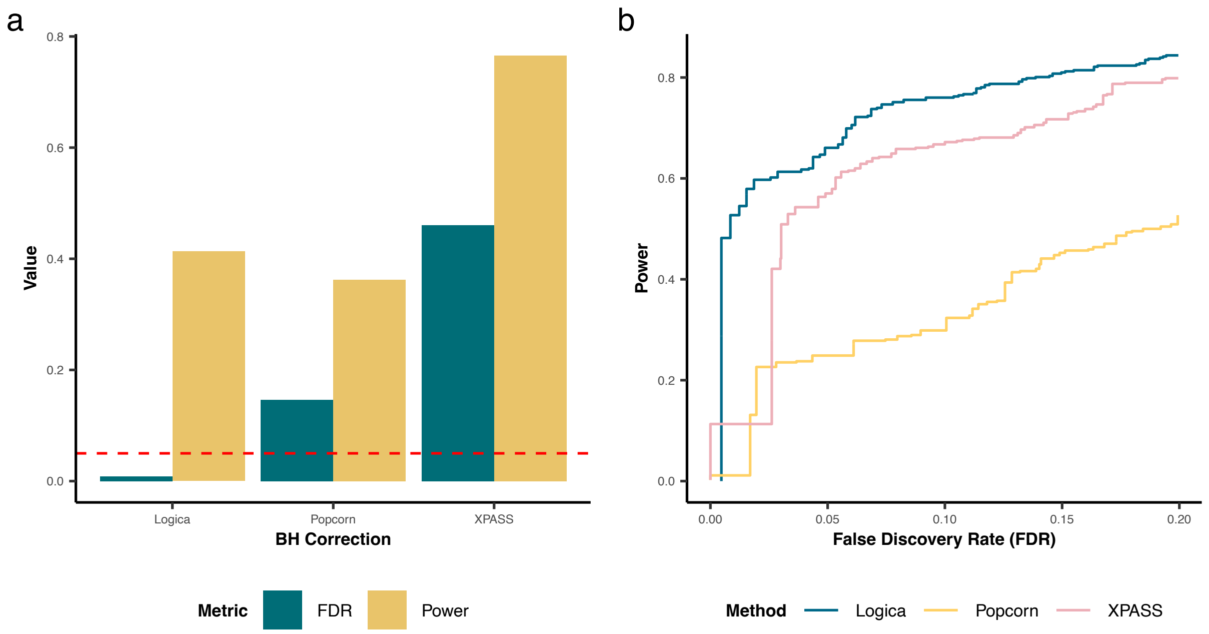

Figure S11: Comparison of FDR and Power among methods for detecting local genetically correlated regions across ancestries in the setting with *population stratification*. We declare a region as genetically correlated if its Benjamini-Hochberg (BH)-adjusted P-value is below a specified threshold. Power is defined as the number of detected signals divided by the total number of genetically correlated regions. False Discovery Rate (FDR) is defined as the number of falsely detected regions divided by the total number of detected genetically correlated regions. Left panel: FDR (dark green) and power (yellow) are compared among methods based on a BH-adjusted P-value threshold of 0.05. The dashed red line indicates the nominal FDR threshold of 0.05. Right panel: To enable fair comparison, an FDR-power plot is presented comparing the power of Logica (blue), Popcorn (yellow), and XPASS (pink) across different FDR levels.

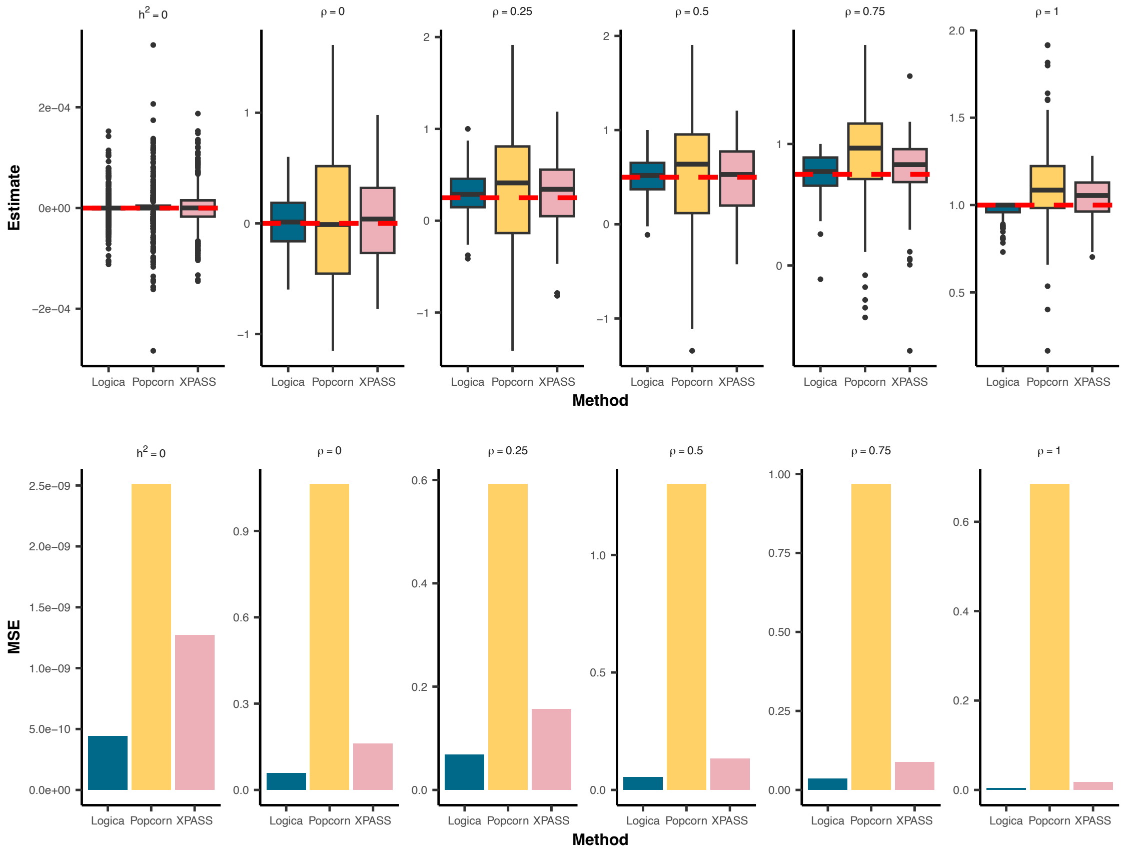

Figure S12: Comparison of local genetic correlation estimation methods in setting with *external LD*. Results are shown for the baseline simulation setting with n = 1,368 independent regions across the genome. The simulated sample size for both ancestries is set to 300,000, and external LD matrices are used for model fitting. Among these regions, 40% have zero heritability in both ancestries, 20% exhibit non-zero heritability in only one ancestry with equal probability, and 40% exhibit non-zero heritability in both ancestries. The heritability of these regions is set to either 3×10^{-5} or 5×10^{-5} with equal probability. For regions exhibiting non-zero heritability in both ancestries, genetic correlation is set to 0, 0.25, 0.5, 0.75, or 1 with equal probability. From left to right, columns represent scenarios with zero heritability in at least one ancestry, and scenarios with non-zero heritability in both ancestries, each varying by genetic correlation (0, 0.25, 0.5, 0.75, 1). We compare the performance of Logica (blue) against Popcorn (yellow) and XPASS (pink). The top panel shows boxplots of estimated local genetic correlations. The bottom panel displays bar plots of mean squared error (MSE) across different true local genetic correlation values.

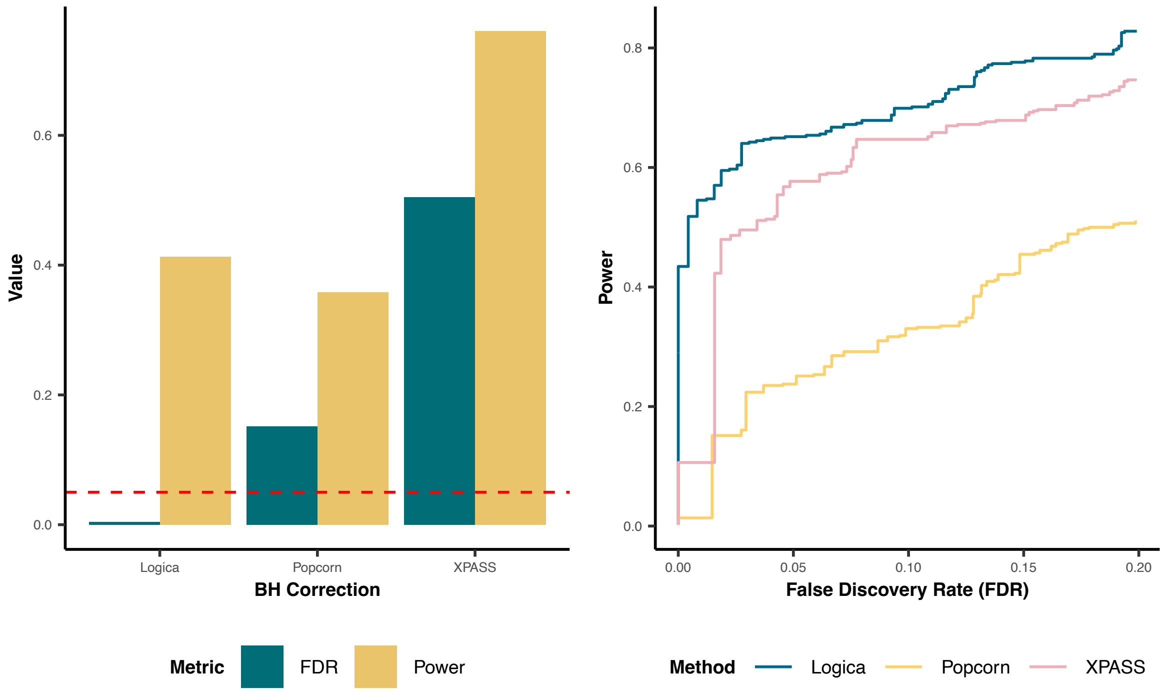

Figure S13: Comparison of FDR and Power among methods for detecting local genetically correlated regions across ancestries in setting with *external LD*. We declare a region as genetically correlated if its Benjamini-Hochberg (BH)-adjusted P-value is below a specified threshold. Power is defined as the number of detected signals divided by the total number of genetically correlated regions. False Discovery Rate (FDR) is defined as the number of falsely detected regions divided by the total number of detected genetically correlated regions. Left panel: FDR (dark green) and power (yellow) are compared among methods based on a BH-adjusted P-value threshold of 0.05. The dashed red line indicates the nominal FDR threshold of 0.05. Right panel: To enable fair comparison, an FDR-power plot is presented comparing the power of Logica (blue), Popcorn (yellow), and XPASS (pink) across different FDR levels.

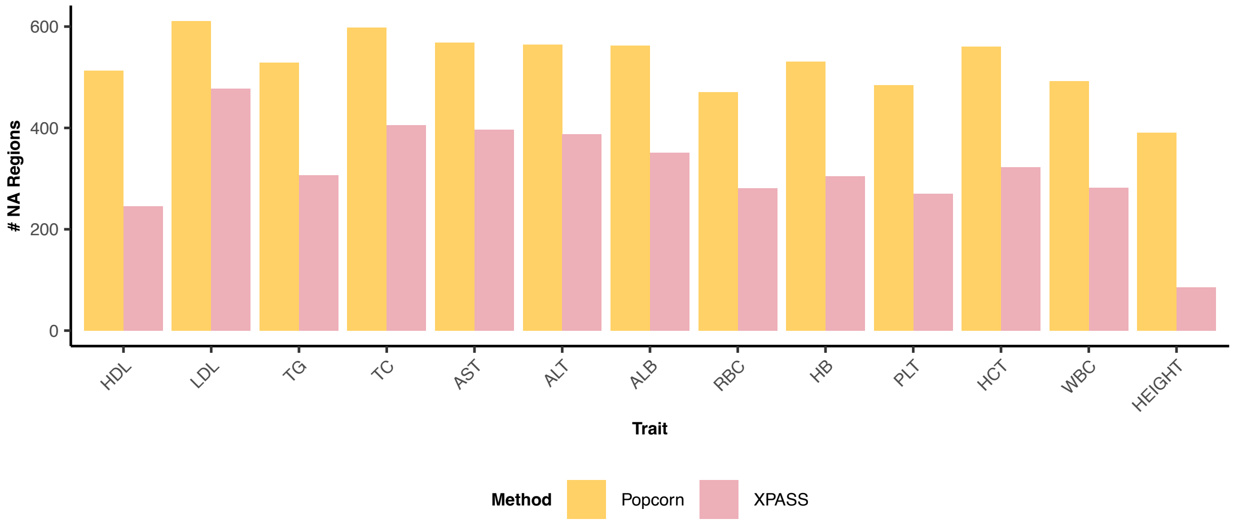

Figure S14: Number of genomic regions for which Popcorn and XPASS failed to provide local genetic correlation estimates across 13 traits in the real data.

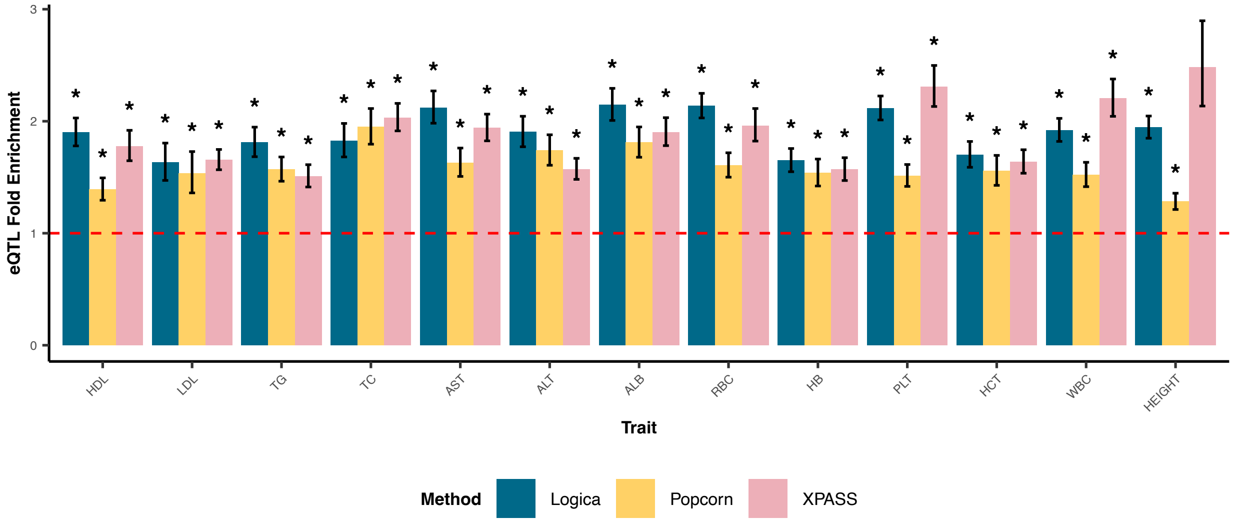

Figure S15: eQTL fold enrichment across detected genetically correlated regions across 13 traits. eQTL fold enrichment is calculated as the ratio of eQTLs within detected genetically correlated regions to those outside such regions. A chi-squared test is used to evaluate statistical significance of enrichment, with asterisks indicating traits with significant eQTL enrichment.

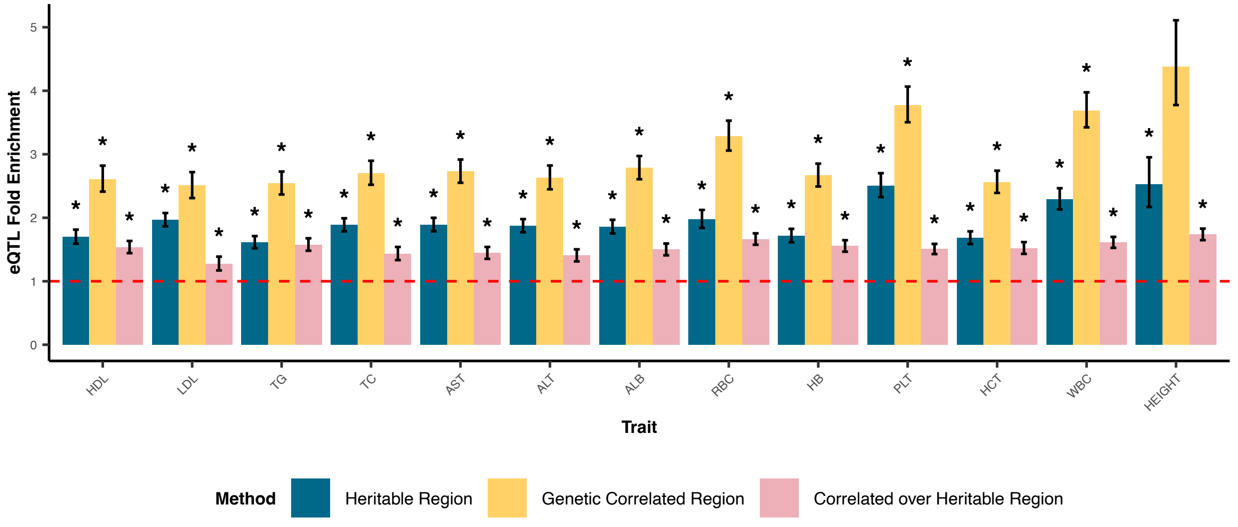

Figure S16: eQTL fold enrichment across genomic regions categorized by heritability and genetic correlation status for 13 complex traits. Regions are grouped based on Logica's testing results into three categories: (1) regions with zero heritability in both ancestries; (2) regions with non-zero heritability in only one ancestry, or in both ancestries but without significant genetic correlation; and (3) regions with non-zero heritability in both ancestries and significant genetic correlation. We performed eQTL enrichment analysis separately for each category comparison: category 2 vs. category 1, category 3 vs. category 2, and category 3 vs. category 1. eQTL fold enrichment is calculated as the ratio of eQTL within regions of a category to those outside such regions. A chi-squared test is used to evaluate statistical significance of enrichment, with asterisks indicating traits with significant eQTL enrichment.

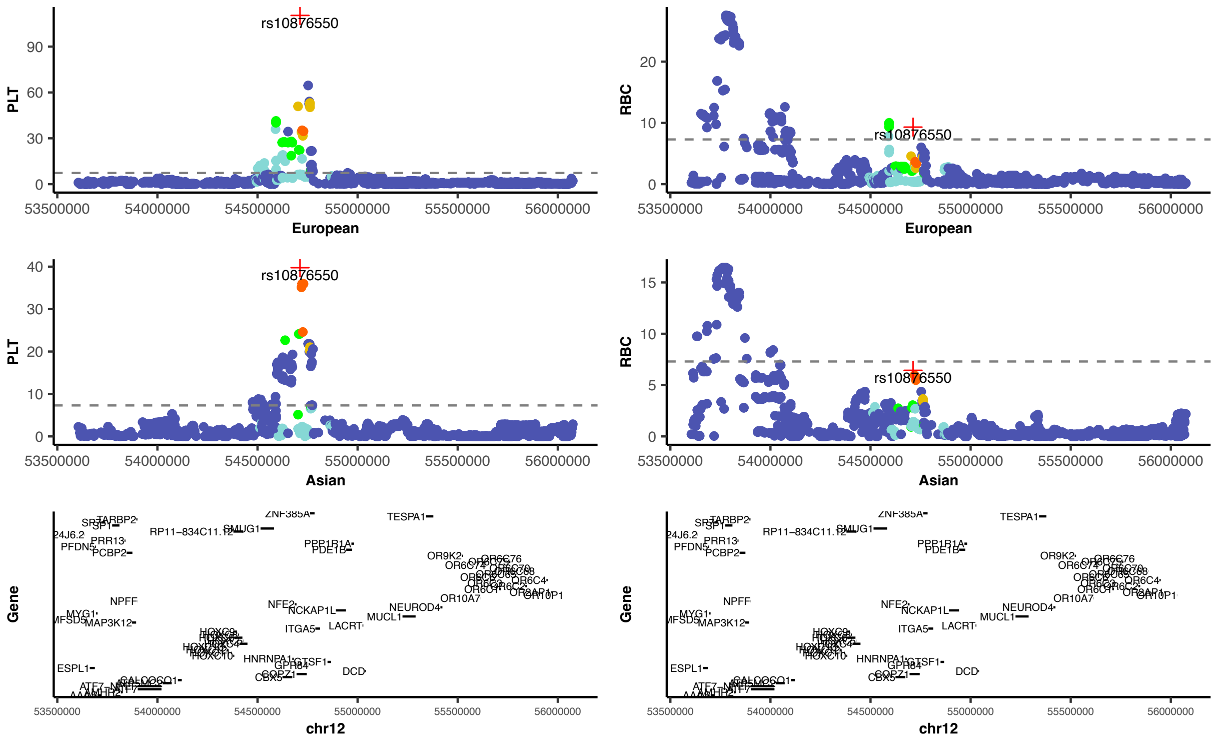

PLT

	Trait	Logica	Popcorn	XPASS
Correlation	PLT	0.82	−1.36	0.01
P−value	PLT	6e−07	0.73	0.95

RBC

	Trait	Logica	Popcorn	XPASS
Correlation	RBC	0.95	1.01	0.56
P−value	RBC	8.4e−05	0.67	1.2e−21

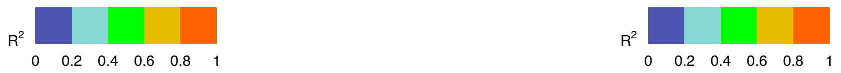

Figure S17: Genetic correlation analysis of PLT and RBC in a genomic region on chromosome 12. Top: LocusZoom plots showing marginal GWAS results (-log10 P-value, y-axis) for PLT (left column) and RBC (right column) across base pair positions (x-axis) in European ancestry. SNP colors indicate linkage disequilibrium (LD, measured as R²) with the lead variant rs410876550. **Second row:** LocusZoom plots of marginal GWAS results for PLT (left) and RBC (right) in East Asian ancestry, with the same color scheme and LD reference variant. **Third row:** Gene annotations within the genomic region, highlighting the candidate gene NFE2. **Bottom:** Table summarizing genetic correlation estimates and their associated P-values from Logica and compared methods for PLT (left) and RBC (right).

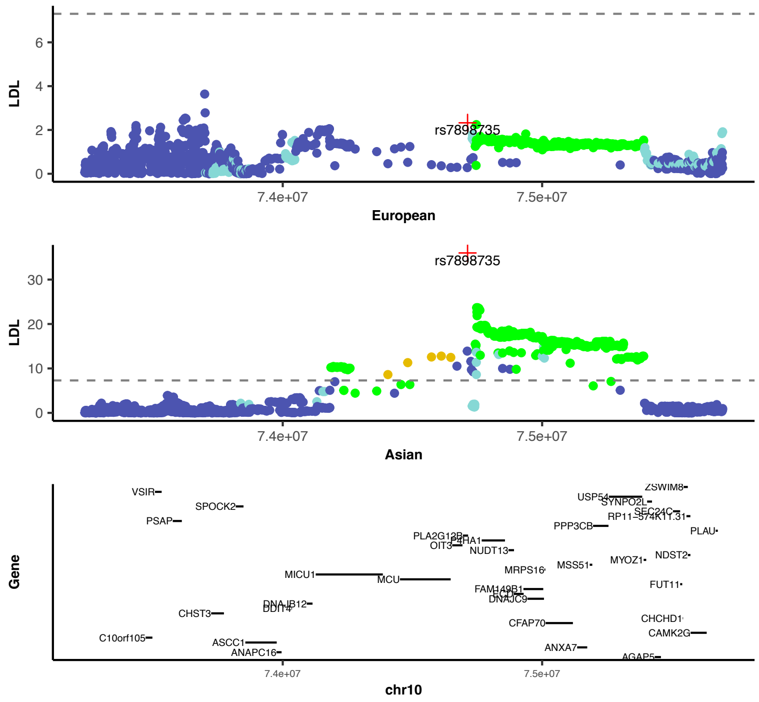

LDL

	Trait	Logica	Popcorn	XPASS
Correlation	LDL	0.57	1.16	1
P−value	LDL	0.34	2e−11	0e+00

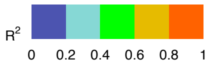

Figure S18: Genetic correlation analysis of LDL in a genomic region on chromosome 10. Top: LocusZoom plots showing marginal GWAS results (-log10 P-value, y-axis) for LDL across base pair positions (x-axis) in European ancestry. SNP colors indicate linkage disequilibrium (LD, measured as R²) with the lead variant rs7898735. **Second row:** LocusZoom plots of marginal GWAS results for LDL in East Asian ancestry, with the same color scheme and LD reference variant. **Third row:** Gene annotations within the genomic region. **Bottom:** Table summarizing genetic correlation estimates and their associated P-values from Logica and compared methods for LDL.

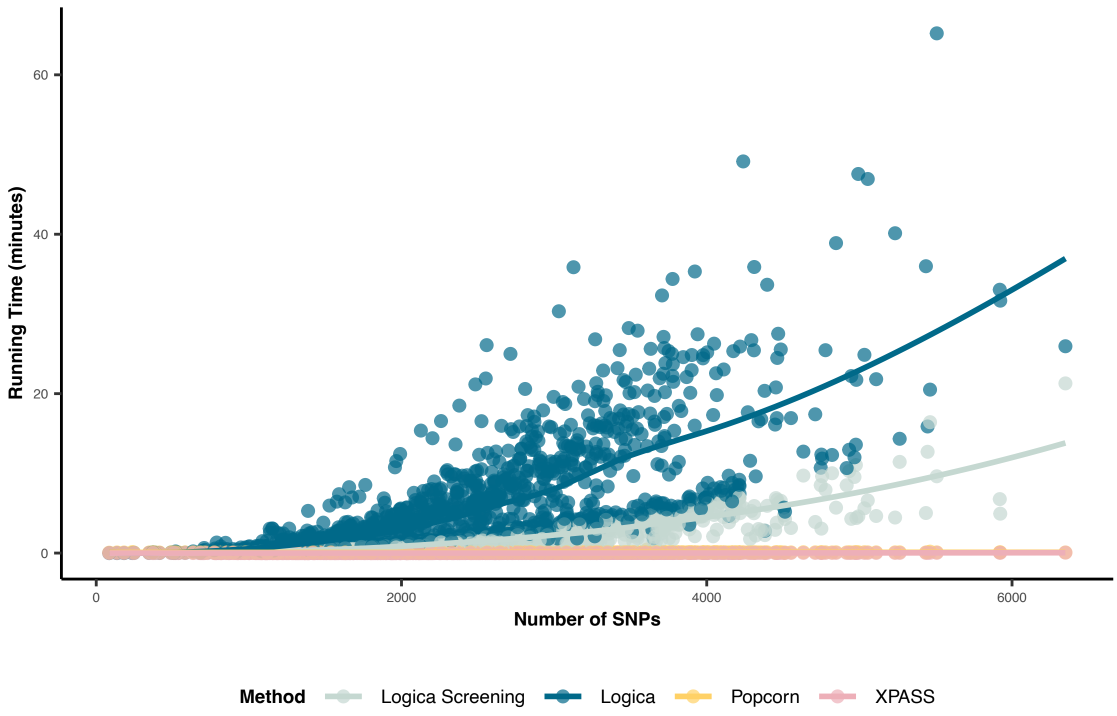

Figure S19: Comparison of computation time. The scatterplot displays the computation time in the baseline setting. The x-axis is the number of SNPs within the locus, the y-axis is the computation time over replicates.

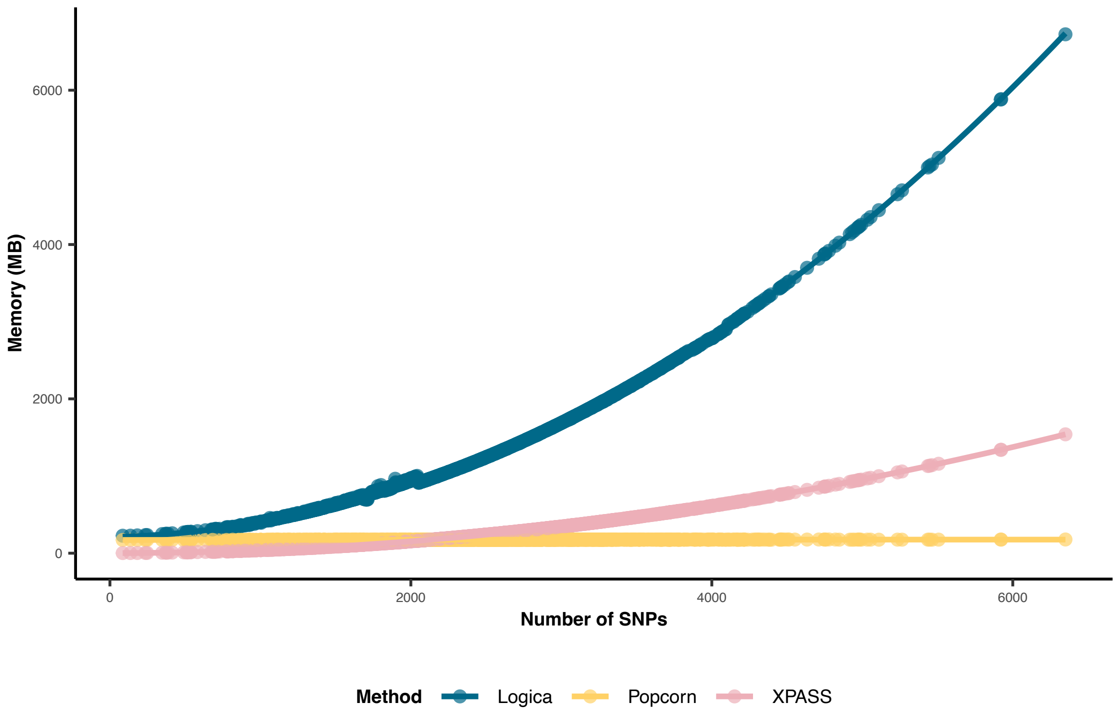

Figure S20: Comparison of peak memory usage. The scatterplot displays the peak memory usage in the baseline setting. The x-axis is the number of SNPs within the locus, the y-axis is the peak memory usage over replicates.

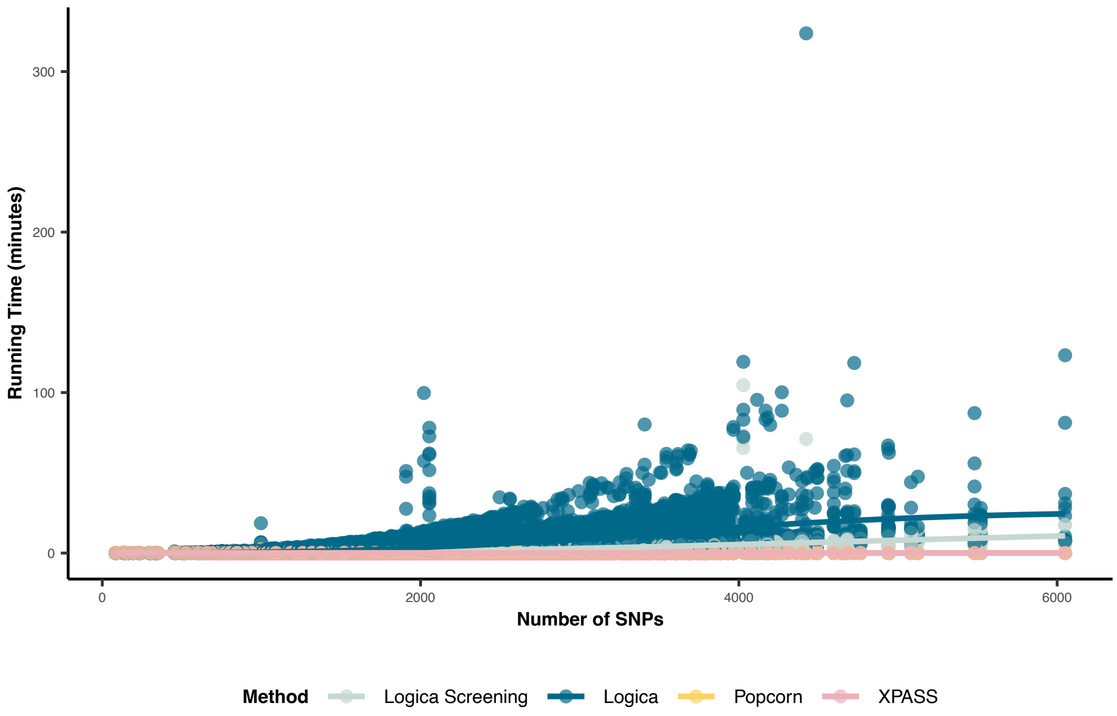

Figure S21: Comparison of computation time in real data analysis. The scatterplot displays the computation time in the real data analysis. The x-axis is the number of SNPs within the locus, the y-axis is the computation time over replicates.

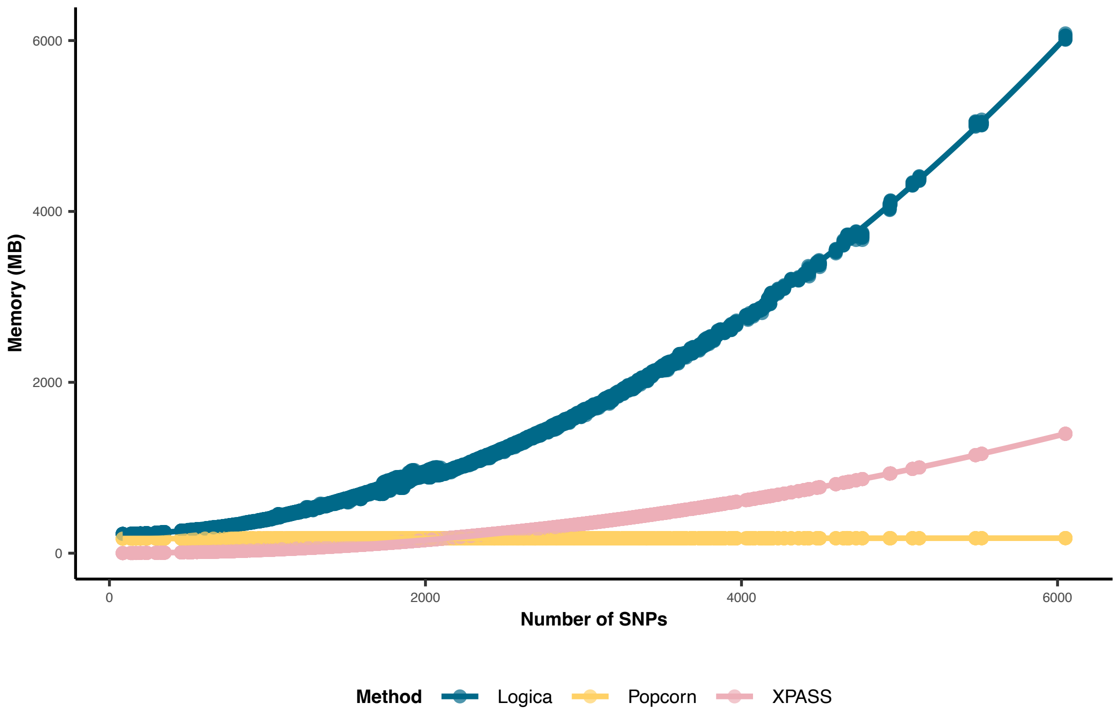

Figure S22: Comparison of peak memory usage in real data analysis. The scatterplot displays the peak memory usage in the real data analysis. The x-axis is the number of SNPs within the locus, the y-axis is the peak memory usage over replicates.

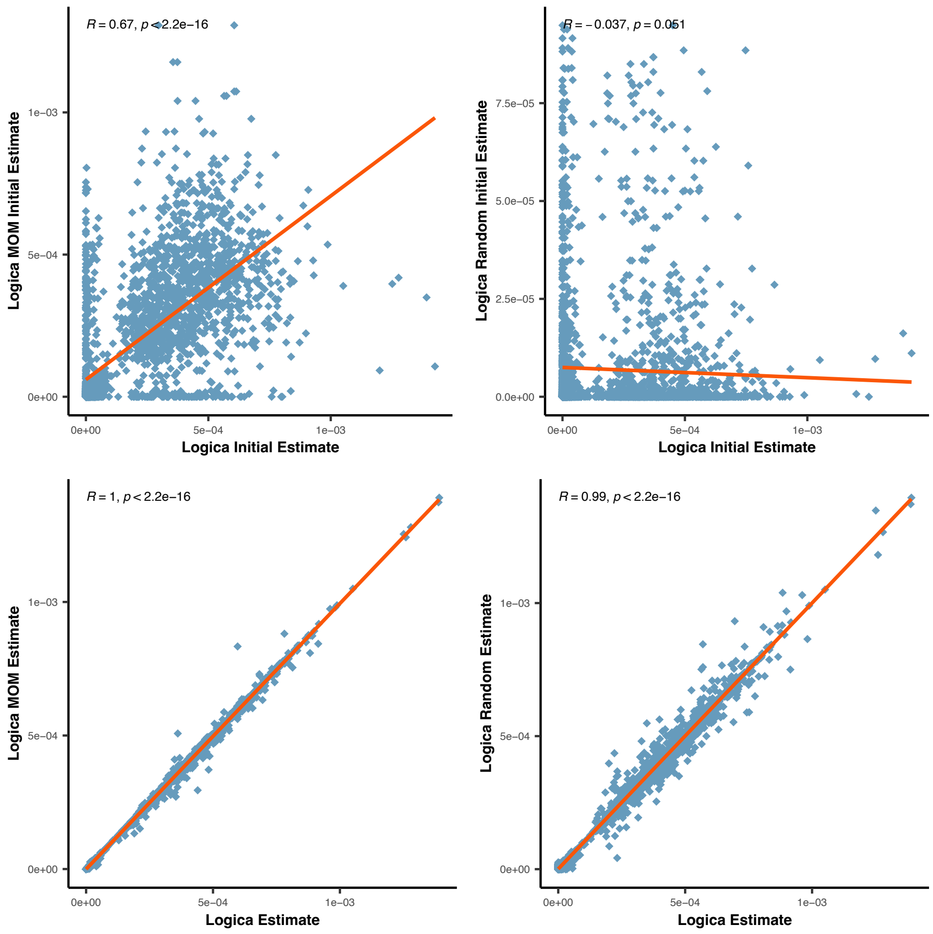

Figure S23: Comparison of local heritability estimates from Logica under different initializations. Top panels show scatter plots comparing Logica initial estimates (x-axis) with those obtained using MoM initialization (left) and random initialization (right). Bottom panels show scatter plots comparing Logica final local heritability estimates (x-axis) with corresponding estimates obtained using MoM initialization (left) and random initialization (right).

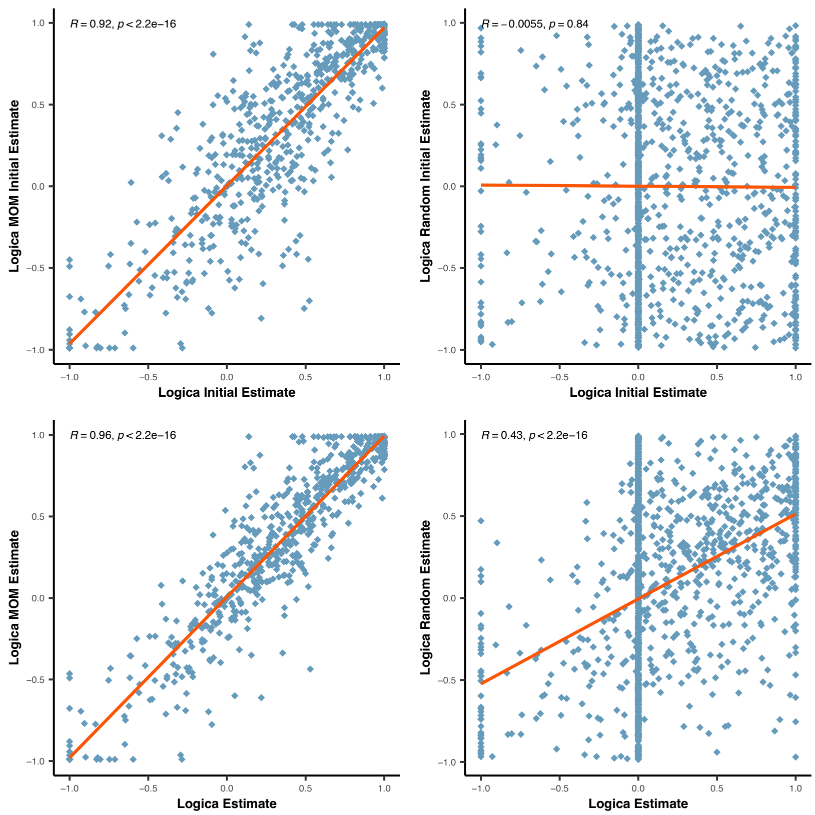

Figure S24: Comparison of local genetic correlation estimates from Logica under different initializations. Top panels show scatter plots comparing Logica initial estimates (x-axis) with those obtained using MoM initialization (left) and random initialization (right). Bottom panels show scatter plots comparing Logica final local heritability estimates (x-axis) with corresponding estimates obtained using MoM initialization (left) and random initialization (right).

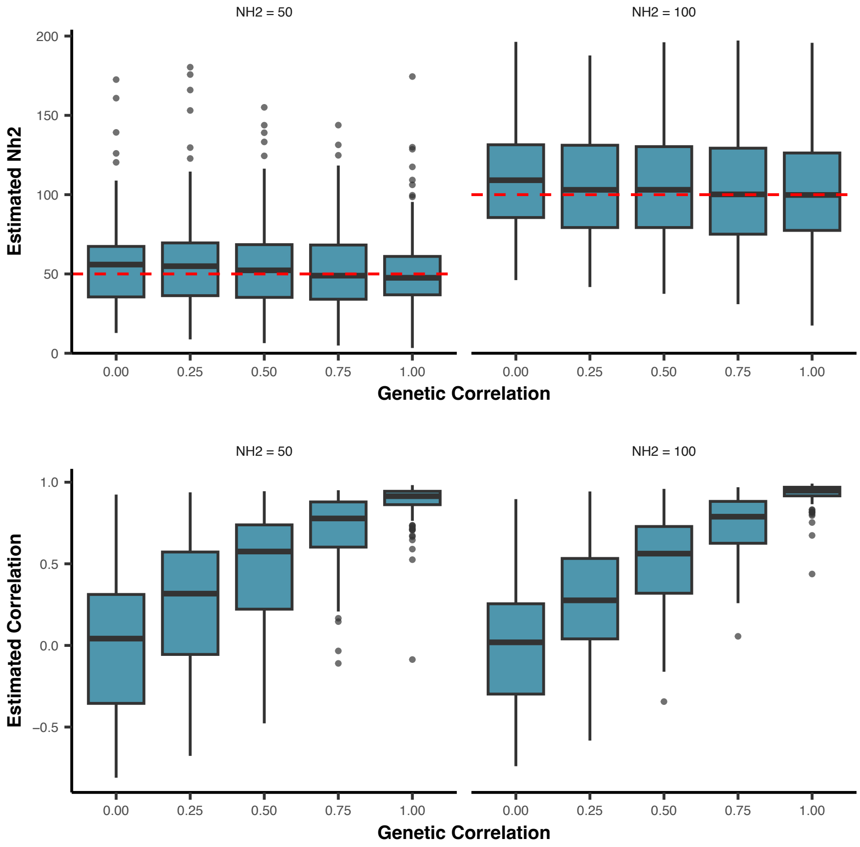

Figure S25: Accuracy of Logica estimates under different genetic correlations and $\boldsymbol{Nh^2}$. Top panels: Top panels: boxplots of estimated local heritability across simulations with $Nh^2 = 50$ (left) and $Nh^2 = 50$ (right). Bottom panels: boxplots of estimated local genetic correlation across the same settings.

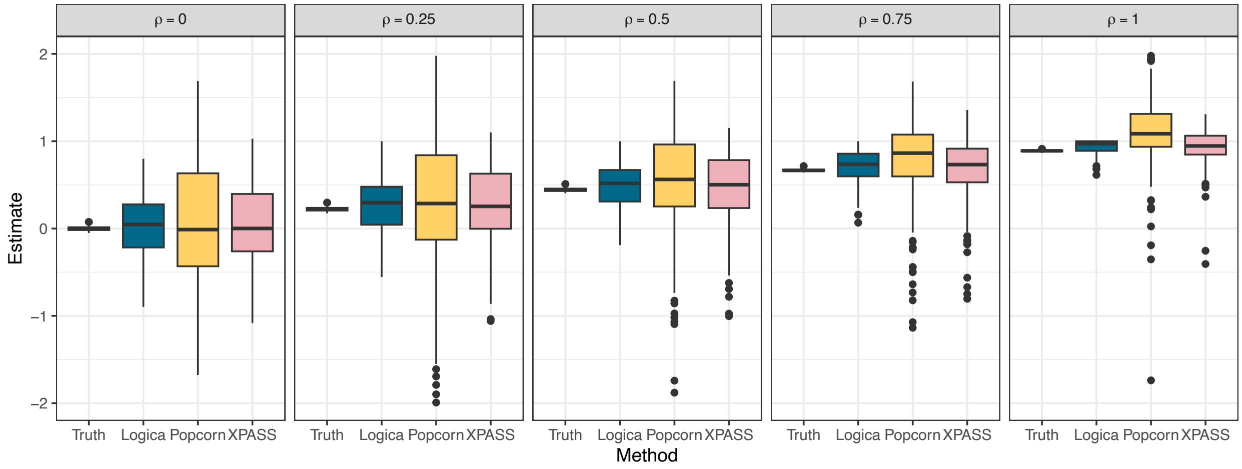

Figure S26: Local genetic correlation in the presence of population-specific variants. Boxplots of estimated local genetic correlation from Logica, Popcorn, and XPASS under simulation settings with shared-variant correlations of 0, 0.25, 0.5, 0.75, and 1. Population-specific variants were included with effect sizes set to zero in the other ancestry; "Truth" denotes the effect size correlation computed from all variants.

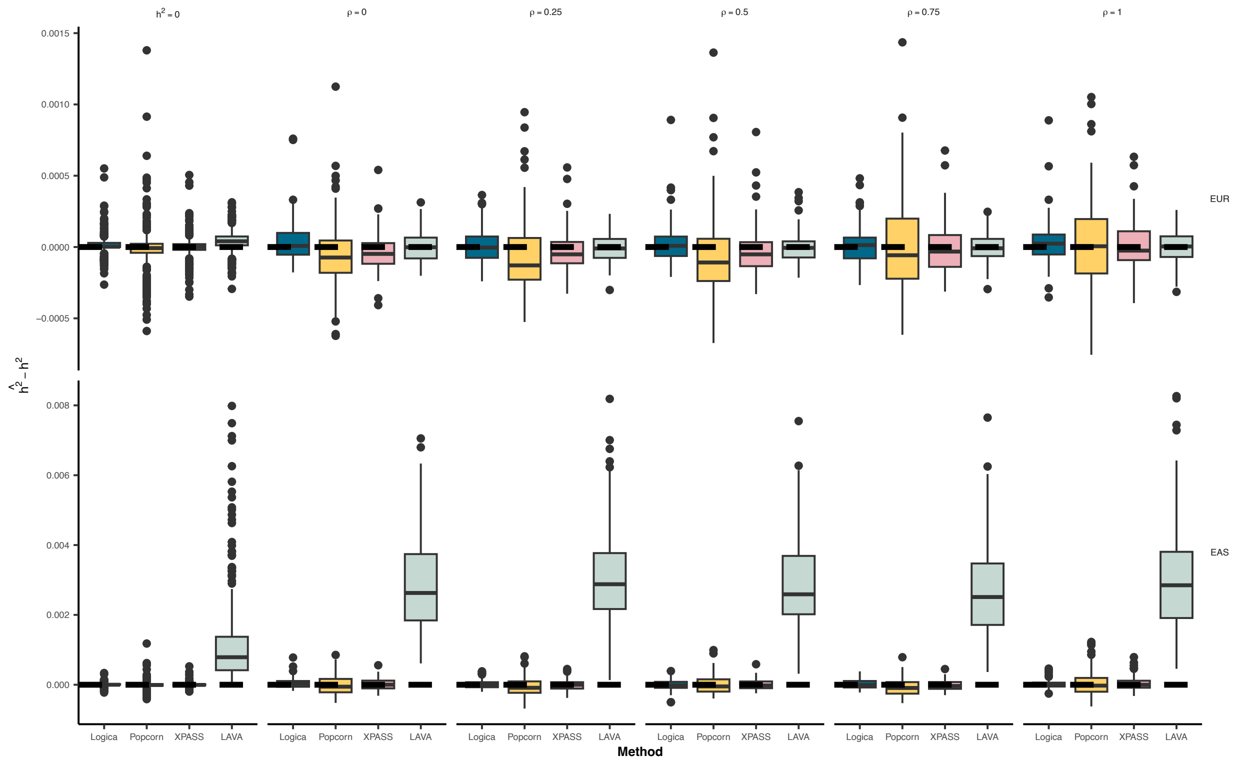

Figure S27: Comparison of local heritability estimates of different methods in baseline simulation setting including LAVA. The heritability of these regions is set to either 3×10^{-5} or 5×10^{-5} with equal probability. For regions exhibiting non-zero heritability in both ancestries, genetic correlation is set to 0, 0.25, 0.5, 0.75, or 1 with equal probability. From left to right, columns represent scenarios with zero heritability in at least one ancestry, and scenarios with non-zero heritability in both ancestries, each varying by genetic correlation (0, 0.25, 0.5, 0.75, 1). We compare the performance of Logica (blue) against Popcorn (yellow), XPASS (pink) and LAVA (green). LAVA is originally a cross-trait local correlation method within a single ancestry; here it is applied with a European LD panel to illustrate its performance in a cross-ancestry setting. The top panel shows boxplots of estimated heritability – true heritability in European ancestry. The bottom panel shows boxplots of estimated heritability – true heritability in East Asian ancestry.

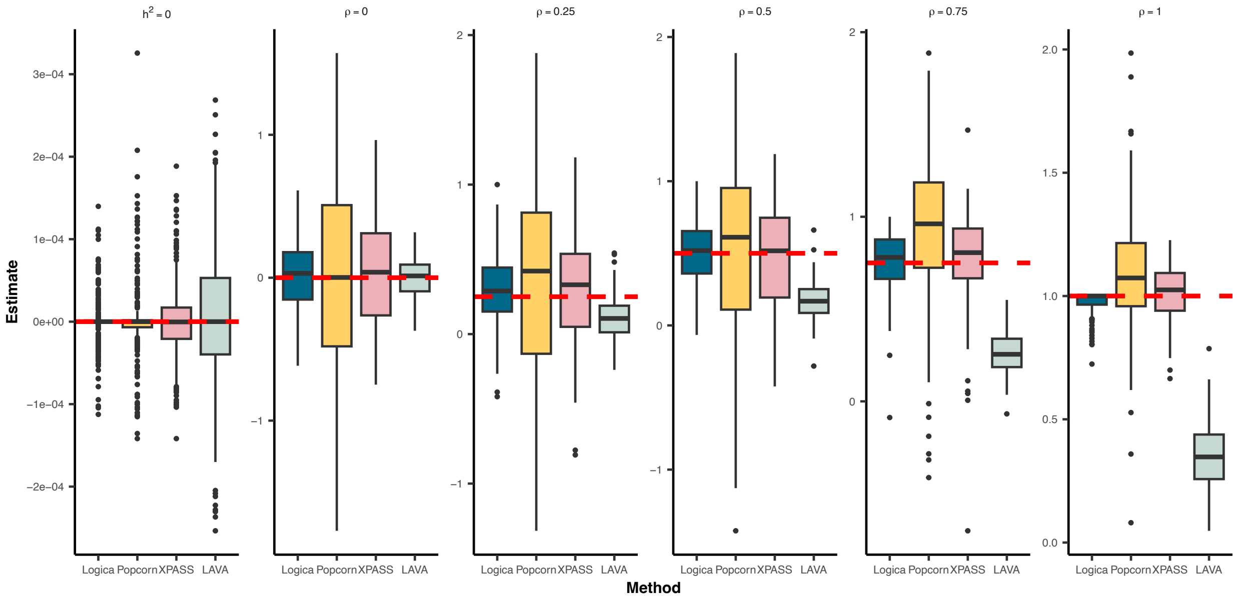

Figure S28: Comparison of local genetic correlation estimates of different methods in baseline simulation setting including LAVA. From left to right, columns represent scenarios with zero heritability in at least one ancestry, and scenarios with non-zero heritability in both ancestries, each varying by genetic correlation (0, 0.25, 0.5, 0.75, 1). We compare the performance of Logica (blue) against Popcorn (yellow), XPASS (pink) and LAVA (green). LAVA is originally a cross-trait local correlation method within a single ancestry; here it is applied with a European LD panel to illustrate its performance in a cross-ancestry setting. The panel shows boxplots of estimated genetic correlations.

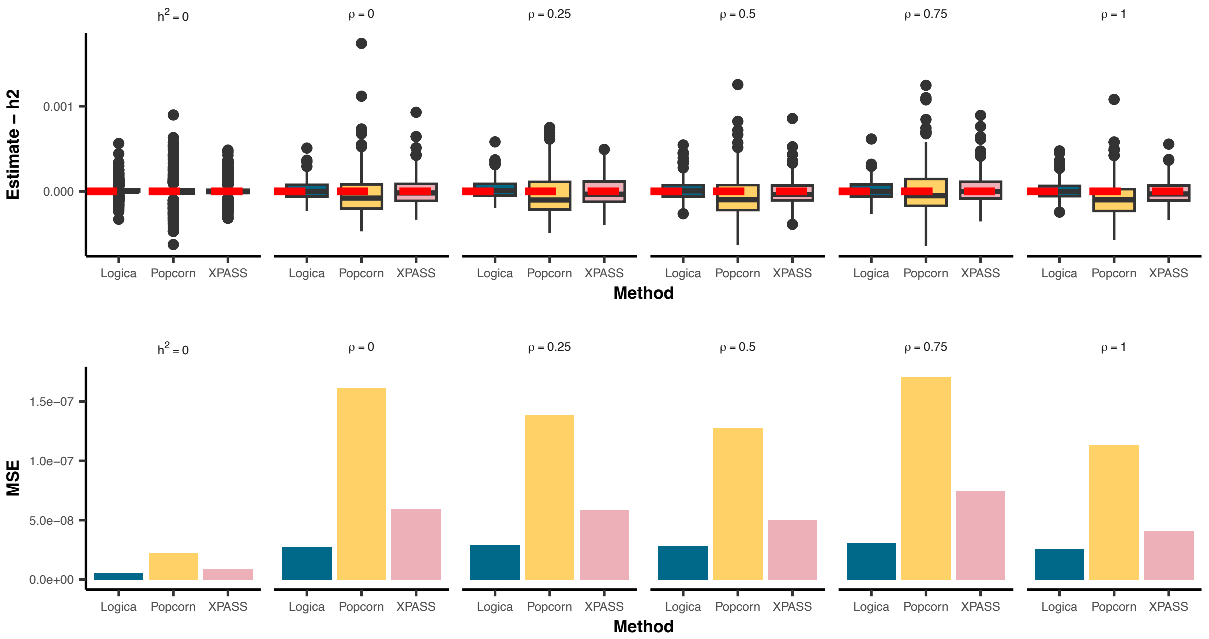

Figure S29: Comparison of local heritability estimates of different methods in 1000G reference panel simulation. Results are shown for the baseline simulation setting with n = 1,368 independent regions across the genome. The simulated sample size for both ancestries is set to 300,000, and in-sample LD matrices are used for model fitting. Among these regions, 40% have zero heritability in both ancestries, 20% exhibit non-zero heritability in only one ancestry with equal probability, and 40% exhibit non-zero heritability in both ancestries. The heritability of these regions is set to either 3×10^{-5} or 5×10^{-5} with equal probability. For regions exhibiting non-zero heritability in both ancestries, genetic correlation is set to 0, 0.25, 0.5, 0.75, or 1 with equal probability. From left to right, columns represent scenarios with zero heritability in at least one ancestry, and scenarios with non-zero heritability in both ancestries, each varying by genetic correlation (0, 0.25, 0.5, 0.75, 1). We compare the performance of Logica (blue) against Popcorn (yellow) and XPASS (pink). The top panel shows boxplots of estimated heritability – true heritability. The bottom panel displays bar plots of mean squared error (MSE) of heritability estimates across different true local genetic correlation values.

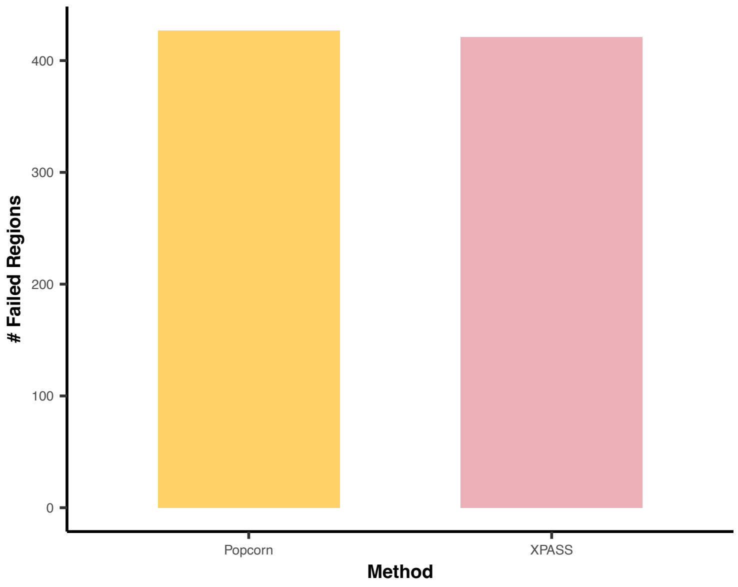

Figure S30: Number of genomic regions for which Popcorn and XPASS failed to provide local genetic correlation estimates in the 1000G reference panel simulation setting.

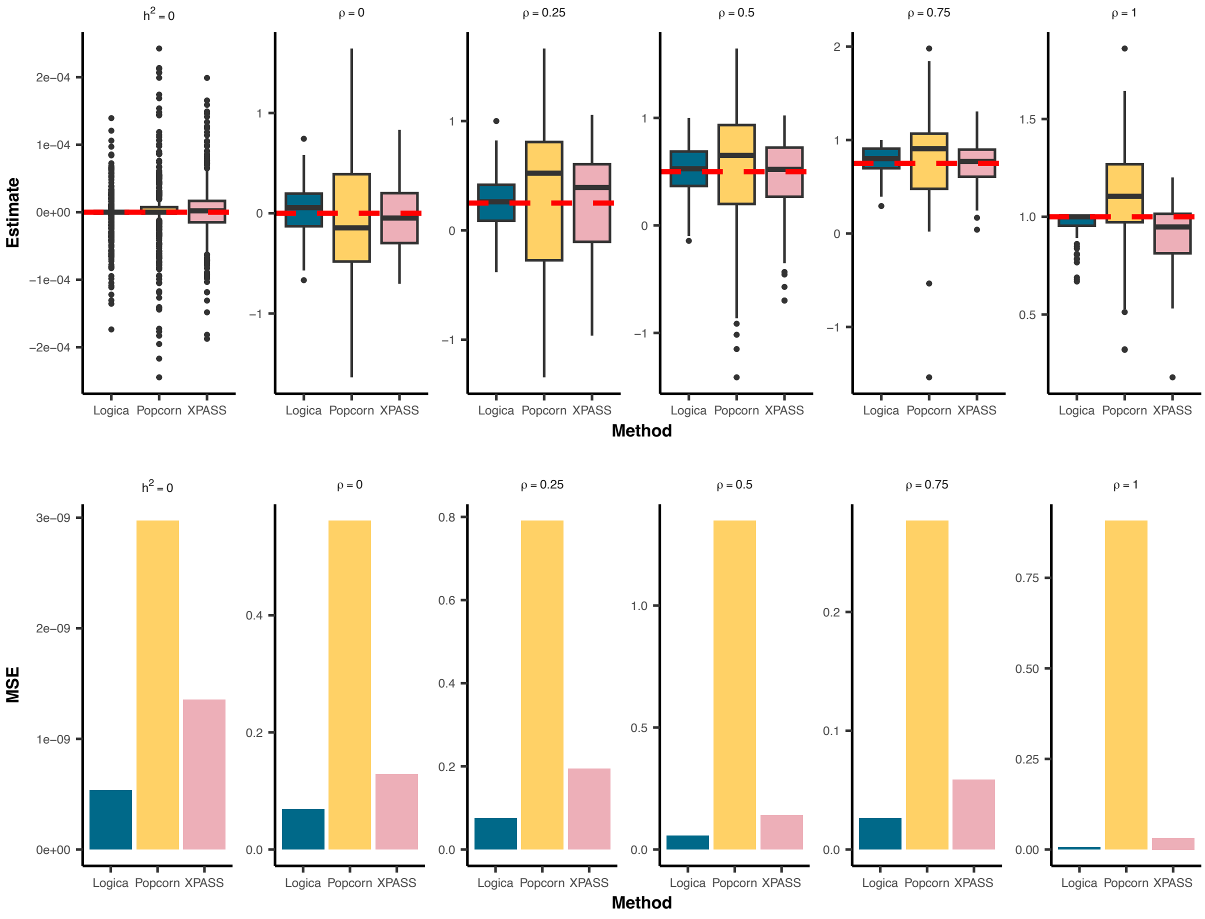

Figure S31: Comparison of methods for local genetic correlation estimation accuracy in the 1000G reference panel simulation setting. From left to right, columns represent scenarios with zero heritability in at least one ancestry ($h^2 = 0$), and scenarios with non-zero heritability in both ancestries, each varying by genetic correlation (ρ =0, 0.25, 0.5, 0.75, or 1). Because genetic correlation is undefined when $h^2 = 0$, the left column reports the cross-ancestry covariance .We compare the performance of Logica (blue) against Popcorn (yellow) and XPASS (pink). **Panel a** shows boxplot of the estimated local genetic correlations for each method. The red dashed lines indicate the true genetic correlation values. **Panel b** displays bar plots of the mean squared error (MSE) of the estimates, comparing the performance of each method across different true genetic correlation values.

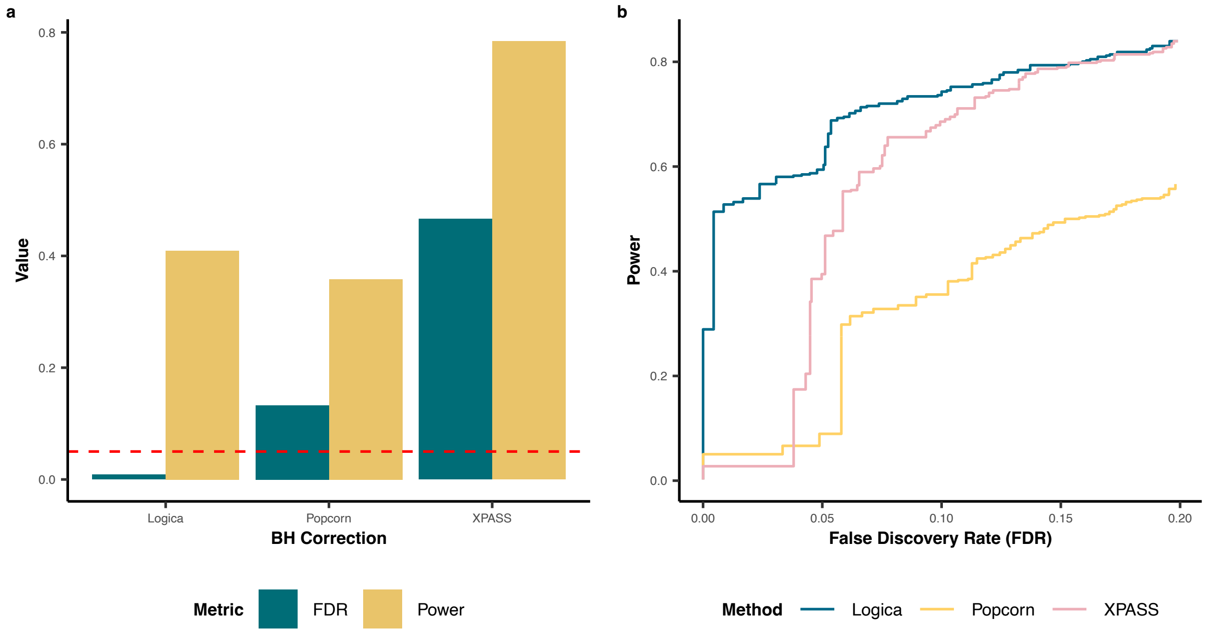

Figure S32: Comparison of FDR and Power among methods for detecting local genetically correlated regions across ancestries in the 1000G reference panel simulation setting. We declare a region as genetically correlated if its Benjamini-Hochberg (BH)-adjusted P-value is below a specified threshold. Power is defined as the number of detected signals divided by the total number of genetically correlated regions. False Discovery Rate (FDR) is defined as the number of falsely detected regions divided by the total number of detected genetically correlated regions. Left panel: FDR (dark green) and power (yellow) are compared among methods based on a BH-adjusted P-value threshold of 0.05. The dashed red line indicates the nominal FDR threshold of 0.05. Right panel: To enable fair comparison, an FDR-power plot is presented comparing the power of Logica (blue), Popcorn (yellow), and XPASS (pink) across different FDR levels.

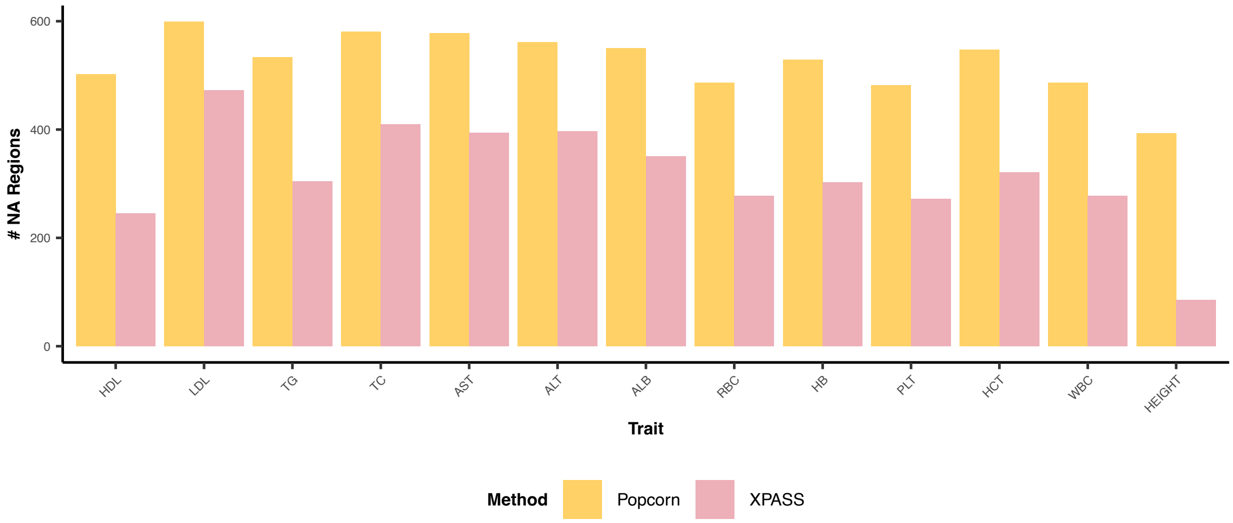

Figure S33: Number of genomic regions for which Popcorn and XPASS failed to provide local genetic correlation estimates across 13 traits in the real data using 1000G reference panel.

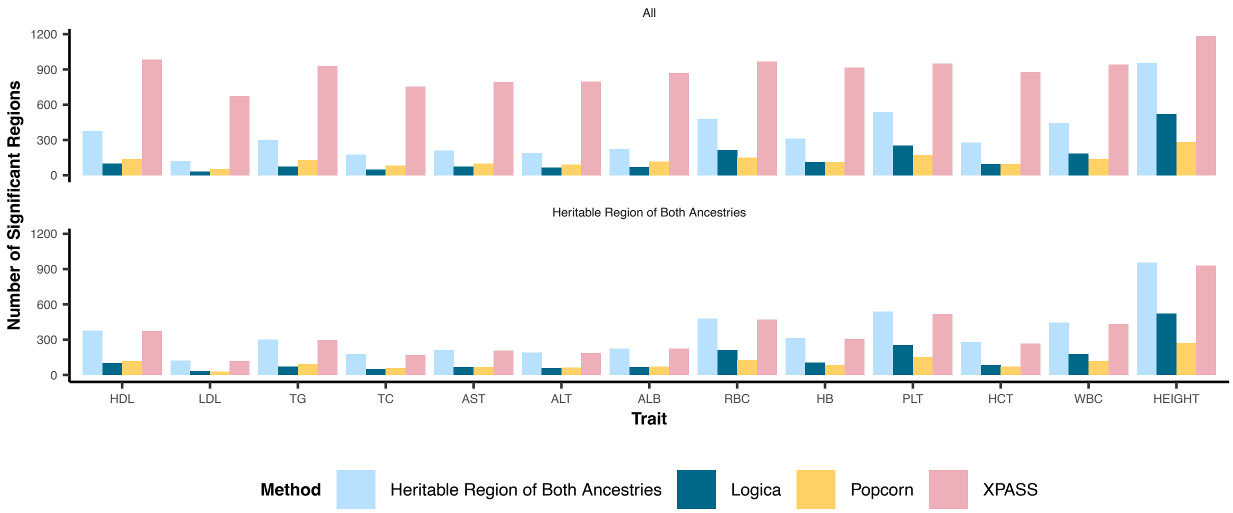

Figure S34: Number of detected genetic correlated region across ancestries for 13 complex traits in the real data application. Using 1000G reference panel. Top panel displays the number of genomic regions detected as heritable in both ancestries by Logica (dark blue), and regions detected as genetically correlated by Logica (dark blue), Popcorn (yellow), and XPASS (pink) across multiple traits. Bottom panel displays the number of genomic regions detected as heritable in both ancestries by Logica (dark blue), and the subset of genetically correlated regions that overlap with these heritable regions identified by Logica (dark blue), Popcorn (yellow), and XPASS (pink).

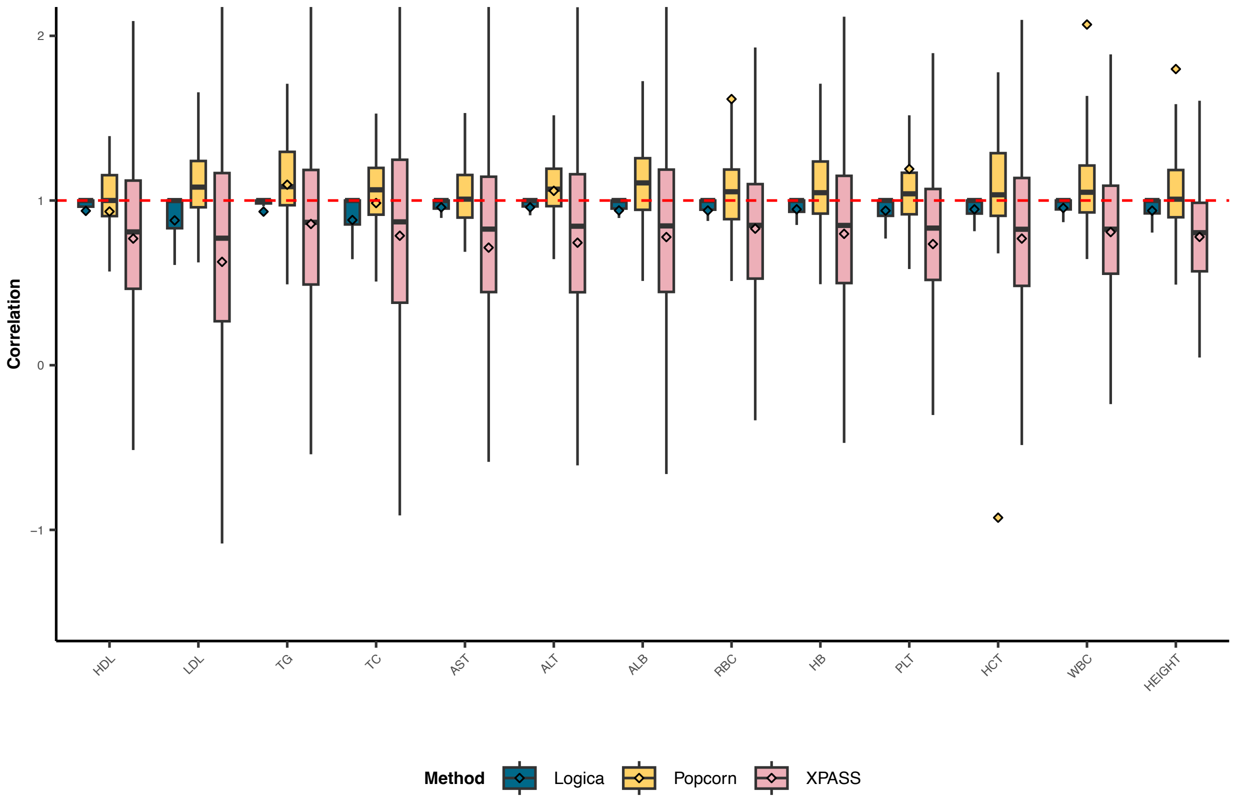

Figure S35: Genetic correlation estimates across ancestries for 13 complex traits in the real data application using 1000G reference panel. Boxplots show the estimated local genetic correlations from detected regions across 13 traits using Logica (blue), Popcorn (yellow), and XPASS (pink).

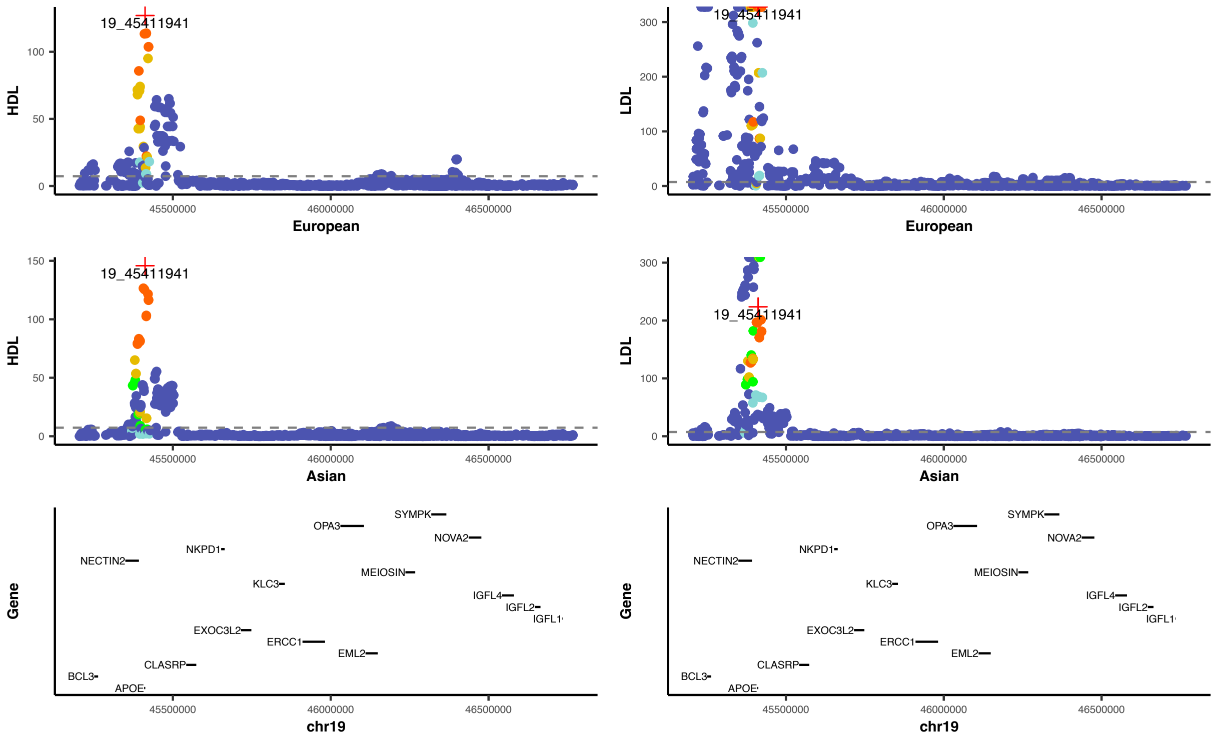

HDL

	Logica	Popcorn	XPASS
Correlation	0.64	2.7	0.91
P−value	4.6e−16	0.18	6.0e−136

LDL

	Logica	Popcorn	XPASS
Correlation	0.42	−0.01	0.07
P−value	1.2e−46	0.97	0.33

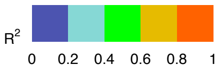

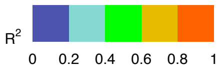

Figure S36: Genetic correlation analysis of HDL and LDL in a genomic region on chromosome 19 with 1000G reference panel. Top: LocusZoom plots showing marginal GWAS results (-log10 P-value, y-axis) for HDL (left column) and LDL (right column) across base pair positions (x-axis) in European ancestry. SNP colors indicate linkage disequilibrium (LD, measured as R^2) with the lead variant rs429358. **Second row:** LocusZoom plots of marginal GWAS results for HDL (left) and LDL (right) in East Asian ancestry, with the same color scheme and LD reference variant. **Third row:** Gene annotations within the genomic region, highlighting the candidate gene APOE. **Bottom:** Table summarizing genetic correlation estimates and their associated P-values from Logica and compared methods for HDL (left) and LDL (right).

Supplemental Table

Setting	Non-heritable/ Heritable in One/ Heritable in Both	Effective SNP Proportion	Local Heritability	ρ	EUR Sample	EAS Sample	LD	Pop-Stratification
Baseline	40%,20%,40%	100%	3e-4,5e-4	0,0.25,0.5,0.75,1	300,000	300,000	In Sample	No
Sparse Setting 1	40%,20%,40%	50%	3e-4,5e-4	0,0.25,0.5,0.75,2	300,000	300,000	In Sample	No
Sparse Setting 2	40%,20%,40%	10%	3e-4,5e-4	0,0.25,0.5,0.75,3	300,000	300,000	In Sample	No
Unbalance Sample size	40%,20%,40%	100%	3e-4,5e-4	0,0.25,0.5,0.75,1	300,000	100,000	In Sample	No
External LD	40%,20%,40%	100%	3e-4,5e-4	0,0.25,0.5,0.75,1	300,000	300,000	External	No
Population Stratification	40%,20%,40%	100%	3e-4,5e-4	0,0.25,0.5,0.75,1	300,000	300,000	In Sample	Yes

Table S1. Overview of simulation settings. This table details the parameters used in the simulation study, including effective SNP proportion (10%, 50%, or 100%), local heritability (SNP heritability within a region), genetic correlation (ρ; correlation of effect sizes across ancestries), EUR Sample (number of European ancestry samples), EAS Sample (number of East Asian ancestry samples), LD (linkage disequilibrium calculated either from in-sample data or external reference data), and Pop-Stratification (presence or absence of population stratification). For all simulation scenarios, we set 40% of regions as non-heritable in both ancestries, 20% heritable in only one ancestry, and 40% heritable in both ancestries.

Trait	Acronym	Category	GWAS	Ancestry	# SNP	Sample Size
Height	HGT	Anthropometric	UKB	EUR	4,995,795	361,194
Red blood cell count	RBC	Hematological	UKB	EUR	4,995,795	361,194
Hemoglobin	HB	Hematological	UKB	EUR	4,995,795	361,194
Platelet count	PLT	Hematological	UKB	EUR	4,995,795	361,194
Hematocrit	HCT	Hematological	UKB	EUR	4,995,795	361,194
White blood cell count	WBC	Hematological	UKB	EUR	4,995,795	361,194
High-density lipoprotein	HDL	Metabolic	UKB	EUR	4,995,795	361,194
Low-density lipoprotein	LDL	Metabolic	UKB	EUR	4,995,795	361,194
Triglycerides	TG	Metabolic	UKB	EUR	4,995,795	361,194
Total cholesterol	TC	Metabolic	UKB	EUR	4,995,795	361,194
Alanine aminotransferase	ALT	Liver Function	UKB	EUR	4,995,795	361,194
Aspartate aminotransferase	AST	Liver Function	UKB	EUR	4,995,795	361,194
Albumin	ALB	Liver Function	UKB	EUR	4,995,795	361,194
Height	HGT	Anthropometric	BJP+KBP+TWB	EAS	3,829,633	329,959
Red blood cell count	RBC	Hematological	BJP+KBP+TWB	EAS	3,829,633	300,491
Hemoglobin	HB	Hematological	BJP+KBP+TWB	EAS	3,829,633	315,705
Platelet count	PLT	Hematological	BJP+KBP+TWB	EAS	3,829,633	361,194
Hematocrit	HCT	Hematological	BJP+KBP+TWB	EAS	3,829,633	299,995
White blood cell count	WBC	Hematological	BJP+KBP+TWB	EAS	3,829,633	361,194
High-density lipoprotein	HDL	Metabolic	BJP+KBP+TWB	EAS	3,829,633	239,882
Low-density lipoprotein	LDL	Metabolic	BJP+KBP+TWB	EAS	3,829,633	237,613
Triglycerides	TG	Metabolic	BJP+KBP+TWB	EAS	3,829,633	276,477
Total cholesterol	TC	Metabolic	BJP+KBP+TWB	EAS	3,829,633	300,720
Alanine aminotransferase	ALT	Liver Function	BJP+KBP+TWB	EAS	3,829,633	313,710
Aspartate aminotransferase	AST	Liver Function	BJP+KBP+TWB	EAS	3,829,633	313,355
Albumin	ALB	Liver Function	BJP+KBP+TWB	EAS	3,829,633	285,450

Table S2. Overview of GWAS data for genetic correlation estimation across European and Asian ancestries.

Est	ρ	Logica	Popcorn	XPASS
$\boldsymbol{h^2 = 0}$	NA	5.92e-07 (-1.12e-04,1.4e-04)	1.03e-06 (-2.77e-04, 3.26e-04)	1.56e-07 (-1.4e-04, 1.9e-04)
$\boldsymbol{h^2 \neq 0}$	0	0.011 (-0.62, 0.61)	0.0042 (-4.16, 2.48)	0.025 (-0.75, 0.97)
$\boldsymbol{h^2 \neq 0}$	0.25	0.29 (-0.42, 1)	0.29 (-2.97, 3.35)	0.29 (-0.81, 1.18)
$\boldsymbol{h^2 \neq 0}$	0.5	0.51 (-0.0805, 1)	0.38 (-17.1, 2.9)	0.48 (-0.42, 1.19)
$\boldsymbol{h^2 \neq 0}$	0.75	0.75 (-0.088, 1)	0.91 (-7.56, 4.56)	0.764 (-0.70, 1.47)
$\boldsymbol{h^2 \neq 0}$	1	0.973 (0.724, 1)	1.16 (0.080, 2.98)	1.02 (0.67, 1.23)

Table S3. Estimated local genetic correlations and covariances across simulation scenarios. For each method (Logica, Popcorn, and XPASS), the table reports the mean estimate across replicates, with the range of observed estimates shown in parentheses. When h2=0h^2 = 0h2=0, genetic correlation is not defined, and the covariance estimate is reported instead. This table provides the numerical summaries corresponding to Figure 2a.

Supplemental Note 1

PX-EM algorithm

In this section, we describe the PX-EM inference algorithm for Logic. Rather than working directly with model (1) in Methods, we consider its parameter-expanded form:

$$\begin{aligned} \boldsymbol{y_1} &= \gamma_1 \boldsymbol{X_1} \boldsymbol{\beta_1} + \boldsymbol{\epsilon_1}, \\ \boldsymbol{y_2} &= \gamma_2 \boldsymbol{X_2} \boldsymbol{\beta_2} + \boldsymbol{\epsilon_2}, \end{aligned} \tag{3}$$

where γ_1 and γ_2 are expansion parameters. To simply the notation, we rewrite the model (3) as

$$\boldsymbol{y} = \boldsymbol{X} * (\boldsymbol{\Gamma}\boldsymbol{\beta}) + \boldsymbol{\epsilon}. \tag{4}$$

Here, $\boldsymbol{y}$ is a catenated phenotype vector $\begin{pmatrix} \boldsymbol{y}_1 \\ \boldsymbol{y}_2 \end{pmatrix}$, $\boldsymbol{X}$ is the catenated genotype matrix $\begin{bmatrix} \boldsymbol{X}_1 & 0 \\ 0 & \boldsymbol{X}_2 \end{bmatrix}$, and $\boldsymbol{\Gamma}$ is the diagonal matrix of expansion parameter $\begin{bmatrix} \gamma_1 \boldsymbol{I}_m & 0 \\ 0 & \gamma_2 \boldsymbol{I}_m \end{bmatrix}$, $\boldsymbol{\beta}$ is the catenated effect size vector $\begin{pmatrix} \boldsymbol{\beta}_1 \\ \boldsymbol{\beta}_2 \end{pmatrix}$, and $\boldsymbol{\epsilon}$ is the catenated random error vector $\begin{pmatrix} \boldsymbol{\epsilon}_1 \\ \boldsymbol{\epsilon}_2 \end{pmatrix}$.

By rewriting the model, the distribution of $\boldsymbol{\beta}$ and $\boldsymbol{\epsilon}$ become:

$$\boldsymbol{\beta} \sim MVN(\boldsymbol{0_{2m}}, \boldsymbol{V} \otimes \boldsymbol{I_m}), \boldsymbol{\epsilon} \sim MVN\left(\boldsymbol{0_{n_1+n_2}}, \boldsymbol{\Sigma_e}\right),$$

where:

$$\boldsymbol{V} = \begin{pmatrix} \frac{h_1^2}{m} & \frac{\rho_g}{m} \\ \frac{\rho_g}{m} & \frac{h_2^2}{m} \end{pmatrix}, \boldsymbol{\Sigma_e} = \begin{pmatrix} \sigma_{e1}^2 \boldsymbol{I_{n_1}} & 0 \\ 0 & \sigma_{e2}^2 \boldsymbol{I_{n_2}} \end{pmatrix}.$$

Let $\theta = \{\gamma_1, \gamma_2, h_1^2, h_2^2, \rho_g, \sigma_{e1}^2, \sigma_{e2}^2\}$ represent the collection of model parameters. Viewing $\boldsymbol{\beta}$ as the latent variable, the complete-data log-likelihood is given by:

$$\begin{aligned} logf(\boldsymbol{y}, \boldsymbol{\beta} | \boldsymbol{\theta}) = -\frac{1}{2}\log(|2\pi\boldsymbol{\Sigma_e}|) - \frac{1}{2}\log(|2\pi\boldsymbol{V} \otimes \boldsymbol{I_m}|) \\ -\frac{1}{2}\{\boldsymbol{y^T \Sigma_e^{-1} y} + \boldsymbol{\beta}^T[\boldsymbol{V^{-1}} \otimes \boldsymbol{I_m} + \boldsymbol{\Gamma^T X^T \Sigma_e^{-1} X \Gamma}]\boldsymbol{\beta} - 2\boldsymbol{\beta^T \Gamma^T X^T \Sigma_e^{-1} y}\}. \end{aligned} \tag{5}$$

E-step

Give current $(\boldsymbol{\theta}^{(t)})$, the conditional distribution of $\boldsymbol{\beta}$ given $\boldsymbol{y}$ and the current estimates of $\boldsymbol{\Sigma}_{\boldsymbol{e}}^{(t)}$ and $\boldsymbol{V}^{(t)}$ is multivariate Gaussian $MVN(\boldsymbol{\mu}_{\boldsymbol{\beta}}^{(t)}, \boldsymbol{\Sigma}_{\boldsymbol{\beta}}^{(t)})$ with:

$$\boldsymbol{\Sigma}_{\boldsymbol{\beta}}^{(t)} = \left[\boldsymbol{V}^{(t)^{-1}} \otimes \mathbf{I_m} + \boldsymbol{\Gamma}^{\mathbf{T}}\mathbf{X}^{\mathbf{T}}\boldsymbol{\Sigma}_{\boldsymbol{e}}^{(t)^{-1}}\mathbf{X}\boldsymbol{\Gamma}\right]^{-1},$$

$$\boldsymbol{\mu}_{\boldsymbol{\beta}}^{(t)} = \left[\boldsymbol{V}^{(t)^{-1}} \otimes \boldsymbol{I_m} + \boldsymbol{\Gamma}^{\boldsymbol{T}}\boldsymbol{X}^{\boldsymbol{T}}\boldsymbol{\Sigma}_{\boldsymbol{e}}^{(t)^{-1}}\boldsymbol{X}\boldsymbol{\Gamma}\right]^{-1} \boldsymbol{\Gamma}^{\boldsymbol{T}}\boldsymbol{X}^{\boldsymbol{T}}\boldsymbol{\Sigma}_{\boldsymbol{e}}^{(t)^{-1}}\boldsymbol{y}.$$

Consequently, $\boldsymbol{E}(\boldsymbol{\beta}^{\boldsymbol{T}}\boldsymbol{A}\boldsymbol{\beta}) = \boldsymbol{\mu}_{\boldsymbol{\beta}}^{\boldsymbol{T}}\boldsymbol{A}\boldsymbol{\mu}_{\boldsymbol{\beta}} + trace(\boldsymbol{A}\boldsymbol{\Sigma}_{\boldsymbol{\beta}})$ for any symmetric matrix $\boldsymbol{A}$.

The Q function is given by:

$$\begin{aligned}
&Q_{\boldsymbol{\beta}|\boldsymbol{y},\theta^{(t)}}\left(\widetilde{\boldsymbol{\theta}}\middle|y,\theta^{(t)}\right) \\
&\quad= -\frac{1}{2}\log\left(\left|2\pi\boldsymbol{\Sigma}_{\boldsymbol{e}}^{(t)}\right|\right) - \frac{1}{2}\log\left(\left|2\pi\boldsymbol{V}^{(t)} \otimes \boldsymbol{I_m}\right|\right) \\
&\quad- \frac{1}{2}\Big\{\boldsymbol{y}^{\boldsymbol{T}}\boldsymbol{\Sigma}_{\boldsymbol{e}}^{(t)^{-1}}\boldsymbol{y} + \boldsymbol{\mu}_{\boldsymbol{\beta}}^{\boldsymbol{T}}\left[\boldsymbol{V}^{(t)^{-1}} \otimes \boldsymbol{I_m} + \boldsymbol{\Gamma}^{\boldsymbol{T}}\boldsymbol{X}^{\boldsymbol{T}}\boldsymbol{\Sigma}_{\boldsymbol{e}}^{(t)^{-1}}\boldsymbol{X}\boldsymbol{\Gamma}\right]\boldsymbol{\mu}_{\boldsymbol{\beta}} \\
&\quad+ trace(\left[\boldsymbol{V}^{(t)^{-1}} \otimes \boldsymbol{I_m} + \boldsymbol{\Gamma}^{\boldsymbol{T}}\boldsymbol{X}^{\boldsymbol{T}}\boldsymbol{\Sigma}_{\boldsymbol{e}}^{(t)^{-1}}\boldsymbol{X}\boldsymbol{\Gamma}\right]\boldsymbol{\Sigma}_{\boldsymbol{\beta}}) - 2\boldsymbol{\mu}_{\boldsymbol{\beta}}^{\boldsymbol{T}}\boldsymbol{\Gamma}^{\boldsymbol{T}}\boldsymbol{X}^{\boldsymbol{T}}\boldsymbol{\Sigma}_{\boldsymbol{e}}^{(t)^{-1}}\boldsymbol{y}\Big\}
\end{aligned}$$

M-step

We updated the parameters by setting the derivative of Q to zero, with the updating equations given as follows

$$\gamma_1^{(t+1)} = \frac{\boldsymbol{\mu}_{\boldsymbol{\beta_1}}^{(t)^{\boldsymbol{T}}}\boldsymbol{X}_{\mathbf{1}}^{\boldsymbol{T}}\boldsymbol{y}_{\mathbf{1}}}{\boldsymbol{\mu}_{\boldsymbol{\beta_1}}^{(t)^{\boldsymbol{T}}}\boldsymbol{X}_{\mathbf{1}}^{\boldsymbol{T}}\boldsymbol{X}_{\mathbf{1}}\boldsymbol{\mu}_{\boldsymbol{\beta_1}}^{(t)} + trace\left(\boldsymbol{X}_{\mathbf{1}}^{\boldsymbol{T}}\boldsymbol{X}_{\mathbf{1}}\boldsymbol{\Sigma}_{\boldsymbol{\beta_{11}}}^{(t)}\right)},$$

$$\gamma_2^{(t+1)} = \frac{\boldsymbol{\mu}_{\boldsymbol{\beta_2}}^{(t)^{\boldsymbol{T}}}\boldsymbol{X}_{\mathbf{2}}^{\boldsymbol{T}}\boldsymbol{y}_{\mathbf{2}}}{\boldsymbol{\mu}_{\boldsymbol{\beta_2}}^{(t)^{\boldsymbol{T}}}\boldsymbol{X}_{\mathbf{2}}^{\boldsymbol{T}}\boldsymbol{X}_{\mathbf{2}}\boldsymbol{\mu}_{\boldsymbol{\beta_2}}^{(t)} + trace\left(\boldsymbol{X}_{\mathbf{2}}^{\boldsymbol{T}}\boldsymbol{X}_{\mathbf{2}}\boldsymbol{\Sigma}_{\boldsymbol{\beta_{22}}}^{(t)}\right)},$$

$$\begin{aligned}
\sigma_{e_1}^{2\ (t+1)} &= \frac{1}{n_1}\Big[\boldsymbol{y}_{\mathbf{1}}^{\boldsymbol{T}}\boldsymbol{y}_{\mathbf{1}} + \gamma_1^{(t+1)^2}\boldsymbol{\mu}_{\boldsymbol{\beta_1}}^{(t)^{\boldsymbol{T}}}\boldsymbol{X}_{\mathbf{1}}^{\boldsymbol{T}}\boldsymbol{X}_{\mathbf{1}}\boldsymbol{\mu}_{\boldsymbol{\beta_1}}^{(t)} + \gamma_1^{(t+1)^2}trace\left(\boldsymbol{X}_{\mathbf{1}}^{\boldsymbol{T}}\boldsymbol{X}_{\mathbf{1}}\boldsymbol{\Sigma}_{\boldsymbol{\beta_{11}}}^{(t)}\right) \\
&\quad - 2\gamma_1^{(t+1)}\boldsymbol{\mu}_{\boldsymbol{\beta_1}}^{(t)^{\boldsymbol{T}}}\boldsymbol{X}_{\mathbf{1}}^{\boldsymbol{T}}\boldsymbol{y}_{\mathbf{1}}\Big],
\end{aligned}$$

$$\begin{aligned}
\sigma_{e_2}^{2\ (t+1)} &= \frac{1}{n_2}\Big[\boldsymbol{y}_{\mathbf{2}}^{\boldsymbol{T}}\boldsymbol{y}_{\mathbf{2}} + \gamma_2^{(t+1)^2}\boldsymbol{\mu}_{\boldsymbol{\beta_2}}^{(t)^{\boldsymbol{T}}}\boldsymbol{X}_{\mathbf{2}}^{\boldsymbol{T}}\boldsymbol{X}_{\mathbf{2}}\boldsymbol{\mu}_{\boldsymbol{\beta_2}}^{(t)} + \gamma_2^{(t+1)^2}trace\left(\boldsymbol{X}_{\mathbf{2}}^{\boldsymbol{T}}\boldsymbol{X}_{\mathbf{2}}\boldsymbol{\Sigma}_{\boldsymbol{\beta_{22}}}^{(t)}\right) \\
&\quad - 2\gamma_2^{(t+1)}\boldsymbol{\mu}_{\boldsymbol{\beta_2}}^{(t)^{\boldsymbol{T}}}\boldsymbol{X}_{\mathbf{2}}^{\boldsymbol{T}}\boldsymbol{y}_{\mathbf{2}}\Big],
\end{aligned}$$

$$\widetilde{\boldsymbol{V}} = \frac{1}{m}\begin{pmatrix} trace\left(\boldsymbol{\mu}_{\boldsymbol{\beta}_1}^{(t)^T}\boldsymbol{\mu}_{\boldsymbol{\beta}_1}^{(t)} + \boldsymbol{\Sigma}_{\boldsymbol{\beta}_{11}}^{(t)}\right) & trace\left(\boldsymbol{\mu}_{\boldsymbol{\beta}_1}^{(t)^T}\boldsymbol{\mu}_{\boldsymbol{\beta}_2}^{(t)} + \boldsymbol{\Sigma}_{\boldsymbol{\beta}_{12}}^{(t)}\right) \\ trace\left(\boldsymbol{\mu}_{\boldsymbol{\beta}_2}^{(t)^T}\boldsymbol{\mu}_{\boldsymbol{\beta}_1}^{(t)} + \boldsymbol{\Sigma}_{\boldsymbol{\beta}_{21}}^{(t)}\right) & trace\left(\boldsymbol{\mu}_{\boldsymbol{\beta}_2}^{(t)^T}\boldsymbol{\mu}_{\boldsymbol{\beta}_2}^{(t)} + \boldsymbol{\Sigma}_{\boldsymbol{\beta}_{22}}^{(t)}\right) \end{pmatrix},$$

where, $\boldsymbol{\mu}_{\boldsymbol{\beta}_1}^{(t)}$ and $\boldsymbol{\mu}_{\boldsymbol{\beta}_2}^{(t)}$ are partitioned vector of $\boldsymbol{\mu}_{\boldsymbol{\beta}}^{(t)}$ in the form of $\boldsymbol{\mu}_{\boldsymbol{\beta}}^{(t)} = \begin{pmatrix} \boldsymbol{\mu}_{\boldsymbol{\beta}_1}^{(t)} \\ \boldsymbol{\mu}_{\boldsymbol{\beta}_2}^{(t)} \end{pmatrix}$, representing the posterior mean of effect sizes for the two ancestries. Similarly $\boldsymbol{\Sigma}_{\boldsymbol{\beta}_{11}}^{(t)}, \boldsymbol{\Sigma}_{\boldsymbol{\beta}_{12}}^{(t)}, \boldsymbol{\Sigma}_{\boldsymbol{\beta}_{21}}^{(t)}$, and $\boldsymbol{\Sigma}_{\boldsymbol{\beta}_{22}}^{(t)}$ are partitioned sub-matrices of $\boldsymbol{\Sigma}_{\boldsymbol{\beta}}^{(t)}$ in the form of $\boldsymbol{\Sigma}_{\boldsymbol{\beta}}^{(t)} = \begin{pmatrix} \boldsymbol{\Sigma}_{\boldsymbol{\beta}_{11}}^{(t)} & \boldsymbol{\Sigma}_{\boldsymbol{\beta}_{12}}^{(t)} \\ \boldsymbol{\Sigma}_{\boldsymbol{\beta}_{21}}^{(t)} & \boldsymbol{\Sigma}_{\boldsymbol{\beta}_{22}}^{(t)} \end{pmatrix}$, each of dimension $m * m$.

After computing the intermediate estimate $\widetilde{\boldsymbol{V}}$ in the expanded space, we apply the reduction step and rescale $\boldsymbol{V} = \boldsymbol{\Gamma}\widetilde{\boldsymbol{V}}\boldsymbol{\Gamma}$, and then set $\gamma_1^{(t+1)}$ and $\gamma_2^{(t+1)}$ to 1.

Initial Values

Since the convergence rate of the PX-EM algorithm can be sensitive to the choice of initial values, we developed an efficient procedure for parameter initialization by separately estimating (h_1^2, σ_{e1}^2) and (h_2^2, σ_{e2}^2) via univariate log-likelihood maximization for each ancestry. For *jth* ancestry, we model:

$$\boldsymbol{y}_{\boldsymbol{j}} = \boldsymbol{X}_{\boldsymbol{j}}\boldsymbol{\beta}_{\boldsymbol{j}} + \boldsymbol{\epsilon}. \tag{6}$$

Multiplying both sides of the above equation by $\boldsymbol{X}_{\boldsymbol{j}}^{\boldsymbol{T}}$, we obtain

$$\boldsymbol{X}_{\boldsymbol{j}}^{\boldsymbol{T}}\boldsymbol{y}_{\boldsymbol{j}} = \boldsymbol{X}_{\boldsymbol{j}}^{\boldsymbol{T}}\boldsymbol{X}_{\boldsymbol{j}}\boldsymbol{\beta}_{\boldsymbol{j}} + \boldsymbol{X}_{\boldsymbol{j}}^{\boldsymbol{T}}\boldsymbol{\epsilon}. \tag{7}$$

Rewriting the above equation, we have:

$$\boldsymbol{Z}_{\boldsymbol{j}} = \sqrt{n_j}\boldsymbol{R}_{\boldsymbol{j}}\boldsymbol{\beta}_{\boldsymbol{j}} + \boldsymbol{X}_{\boldsymbol{j}}^{\boldsymbol{T}}\boldsymbol{\epsilon}/\sqrt{n_j}. \tag{8}$$

Here, $\boldsymbol{Z}_{\boldsymbol{j}}$ represents the *m*-vector of marginal Z-scores and $\boldsymbol{R}_{\boldsymbol{j}}$ is the *m* by *m* LD correlation matrix for the *jth* ancestry (details in *Logica with Summary Statistics*). Consequently, the distribution of $\boldsymbol{Z}_{\boldsymbol{j}}$ follows:

$$\boldsymbol{Z}_{\boldsymbol{j}} \sim MVN\left(0_m, \frac{n_j h_j^2}{m} R_j R_j + \sigma_j^2 \boldsymbol{R}_j\right).$$

The computation is non-trivial as it requires to compute the inverse of m-by-m matrix for each iteration. To mitigate the computational burden and instability associated with inverting $\boldsymbol{R}_j$, we performed eigen-decomposition of the LD matrix $R_j = U_j D_j U^T$, where U_j and D_j represents its eigenvectors and eigenvalues. Multiplying Z_j by $U_j^T D_j^{-\frac{1}{2}}$, we obtain the transformed variable $\tilde{Z}_j$, which follows

$$\tilde{Z}_j \sim MVN\left(0_m, \frac{n_j h_j^2}{m} D_j + \sigma_j^2 I\right).$$

We obtained the initial estimate of h_j^2 and σ_{ej}^2 by maximizing the log-likelihood of $\tilde{Z}_j$ using the *optim* function in R. Consequently, the computational complexity of the initial estimation of h_j^2 and σ_{ej}^2 becomes linear in m in each iteration after the initial eigen-decomposition.

Next, we obtained the initial estimate of ρ_g by maximizing the joint likelihood. Specifically, we define $V = \begin{pmatrix} \frac{h_1^2}{m} & \frac{\rho_g}{m} \\ \frac{\rho_g}{m} & \frac{h_2^2}{m} \end{pmatrix}$ and $\boldsymbol{\Sigma} = \begin{pmatrix} \sigma_{e1}^2 I_{n_1} & 0 \\ 0 & \sigma_{e1}^2 I_{n_2} \end{pmatrix}$ so that $\begin{pmatrix} \boldsymbol{\beta}_1 \\ \boldsymbol{\beta}_2 \end{pmatrix} \sim MVN(0, \boldsymbol{V} \otimes \boldsymbol{I_m})$ and $\begin{pmatrix} \boldsymbol{\epsilon}_1 \\ \boldsymbol{\epsilon}_2 \end{pmatrix} \sim MVN(0, \boldsymbol{\Sigma})$. The joint likelihood can be expressed as

$$l(\rho_g) = -\frac{n_1 + n_2}{2} log 2\pi - \frac{1}{2}\log|\boldsymbol{X}(\boldsymbol{V}\otimes\boldsymbol{I_m})\boldsymbol{X^T} + \boldsymbol{\Sigma}| - \frac{1}{2} y^T [\boldsymbol{X}(\boldsymbol{V}\otimes\boldsymbol{I_m})\boldsymbol{X^T} + \boldsymbol{\Sigma}]^{-1} y.$$

We estimated ρ_g maximizing the above log-likelihood using the Brent algorithm implemented in the *optim* function in R, plugging in the previously estimated values for $\hat{h}_1^2, \hat{h}_2^2, \hat{\sigma}_{e1}^2$, and $\hat{\sigma}_{e2}^2$.

Logica with Summary Statistics

While the PX-EM algorithm is described based on individual-level genotype and phenotype data, it can be easily extended to using only GWAS summary statistics. In particular, we note that the above algorithm can be fitted using $\boldsymbol{X_1^T X_1}, \boldsymbol{X_2^T X_2}, \boldsymbol{X_1^T y_1}, \boldsymbol{X_2^T y_2}, n_1, n_2$, which can be derived based on marginal *Z* scores, LD matrices, and sample size. Specifically, in each GWAS, we define the marginal *Z* scores vector for SNP as *z*, LD matrix as *R*, and the sample size as *n*.

$$\boldsymbol{X^T X} = n * \boldsymbol{R},$$
$$\boldsymbol{X^T y} = \sqrt{n} * z.$$

The assumption made on the standardized genotype matrix can be reduced if both marginal effect size estimate of SNPs and corresponding standard error are given. Specifically, in each GWAS, we define the marginal effect size estimate of *j*'th SNP and its standard error as,

$$\widehat{\beta_J} = \left(\boldsymbol{X_j X_j}\right)^{-1} \boldsymbol{X_j^T y},$$

$$se\left(\widehat{\beta_J}\right) = \sqrt{\widehat{\sigma_J^2}\left(\boldsymbol{X_j X_j}\right)^{-1}},$$

where the estimated residual error variance is in the form of

$$\widehat{\sigma_J}^2 = \frac{n}{\left(\frac{\widehat{\beta_J}}{se\left(\widehat{\beta_J}\right)}\right)^2 + n - 2}.$$

Consequently, we can express

$$\boldsymbol{X_j^T y} = \frac{\widehat{\sigma_J}^2 \widehat{\beta_J}}{se\left(\widehat{\beta_J}\right)^2},$$

$$\boldsymbol{X_j^T X_j} = \frac{\widehat{\sigma_J}^2}{se\left(\widehat{\beta_J}\right)^2}.$$

We further denote

$$\boldsymbol{D_x} = Diag\left(\boldsymbol{X_1^T X_1}, \ldots, \boldsymbol{X_j^T X_j}\right),$$

$$\boldsymbol{X^T X} = \boldsymbol{D_x^{\frac{1}{2}} R D_x^{\frac{1}{2}}}.$$

Therefore, we can obtain the sufficient statistics by using GWAS summary statistics and LD reference panel from each ancestry and then fit Logica using sufficient statistics with PX-EM algorithm described above.

Supplemental Note 2

Assessing the Robustness of the EM Algorithm to Initial Values

We assessed the robustness of our approach by examining alternative initializations through simulations. We first checked the consistency in the estimation and testing of local heritability. Logica MoM initial local heritability estimates were moderately correlated with those from Logica (correlation = 0.67), whereas Logica Random initials showed virtually no correlation with Logica initials (correlation = -0.037). Despite these differences in initialization, the resulting local heritability estimates were highly consistent. Specifically, Logica MoM and Logica Random estimates were strongly correlated with Logica estimates, with correlations of 1 and 0.99, respectively (Figure S23). Logica MoM and Logica Random local heritability estimates exhibited similar performance to Logica with univariate initial values in terms of mean squared error (MSE). Specifically, Logica MoM had a mean MSE of 1.75×10^{-8} (range: 7.95×10^{-86} to 9.61×10^{-7}), Logica Random had a mean MSE of 1.80×10^{-8} (range: 5.72×10^{-27} to 8.73×110^{-7}), which is comparable to Logica's MSE of 1.75×10^{-8} (range: 2.19×10^{-27} to 9.61×10^{-7}).

Next, we assessed the consistency in estimating and testing local genetic correlation. Logica MoM initial estimates of local genetic correlation were highly correlated with those from Logica (correlation = 0.92), whereas Logica Random initials showed essentially no correlation with Logica initials (correlation = –0.0055). The final estimates showed differences across initialization strategies. Logica MoM genetic correlation estimates remained strongly correlated with Logica estimates (correlation = 0.96), whereas Logica Random genetic correlation estimates exhibited a moderate correlation with Logica estimates (correlation = 0.43; Figure S24). This reduction in correlation for the random initialization setting suggests a potential influence of the initial values on the EM estimation results. In terms of accuracy, Logica MoM and the original Logica initialization performed similarly, with mean MSEs of 0.044 (range: 0.0036–0.068) and 0.070 (range: 0.0052–0.11), respectively. By contrast, Logica Random had lower accuracy with a higher mean MSE of 0.25 (range: 0.17–0.34).

Supplemental Note 3

Limitations in Extending Cross-Trait Methods to Cross-Ancestry

We selected LAVA as a representative example which demonstrates robust performance comparable to SUPERGNOVA, and fit LAVA with LD matrix from European reference panel in

our baseline simulation study and benchmarked its performance. In the baseline simulation setting, LAVA yields unbiased local-heritability estimates in European ancestry but exhibits an upward bias in East Asian ancestry (mean bias = 0.0026; range: 0.0010–0.0031; Figure S27). For local genetic correlation, LAVA consistently underestimates the true correlation whenever it departs from zero (Figure S28): the mean estimated correlations are 0.11, 0.17, 0.27, and 0.35 when the true values are 0.25, 0.50, 0.75, and 1.00, respectively.

Supplemental Note 4

Impact of Reference Panel Differences in Simulation and Real Data

We repeated the analyses using 503 European individuals from the 1000G Project, which ensured balanced panel sizes in simulation and the use of external panels for both ancestries in real data. We first checked the baseline simulation setting, Logica consistently yielded the most accurate heritability estimates across different reference panels, with performance remaining stable and robust. When using the 1000G reference panel, Logica achieved a mean MSE of 1.43×10^{-8} (range: 3.34×10^{-23} to 4.23×10^{-7}). Similarly, using the UKB European reference panel, Logica maintained comparable accuracy with a mean MSE of 1.76×10^{-8} (range: 2.19×10^{-27} to 9.61×10^{-7}). In both scenarios, Logica consistently outperformed other methods: XPASS yielded mean MSEs of 2.78×10^{-8} (1000G panel; range: 6.79×10^{-13} to 1.21×10^{-6}) and 2.60×10^{-8} (UKB panel; range: 9.57×10^{-12} to 7.53×10^{-7}), while Popcorn showed mean MSEs of 7.02×10^{-8} (1000G panel; range: 6.98×10^{-13} to 3.17×10^{-6}) and 8.36×10^{-8} (UKB panel; range: 1.58×10^{-13} to 2.50×10^{-6}; Figure S29).

For genetic correlation estimation, similar to using UKB reference panel, Popcorn and XPASS encountered substantial estimation difficulties, failing to produce valid genetic correlation estimates in 31.2% (427 of 1,368) and 30.8% (421 of 1,368) of the genomic regions, respectively (Figure S30). In the succeed regions, all methods provided unbiased genetic correlation estimates across different heritability and genetic correlation settings in regions with non-zero heritability in both ancestries. Logica consistently produced the most accurate estimates, achieving mean MSEs of 0.047 (1000G panel; range: 0.0069 to 0.075) and 0.044 (UKB panel; range: 0.0036 to 0.068). XPASS followed with mean MSEs of 0.11 (1000G panel; range: 0.032 to 3.38) and 0.11 (UKB panel; range: 0.0149 to 0.15), while Popcorn had the highest mean MSEs of 0.78 (1000G panel; range: 0.28 to 1.35) and 1.19 (UKB panel; range: 0.17 to 3.21; Figure S31).

Logica maintained well-controlled false discovery rates (FDR) across both reference panels, achieving FDRs of 0.009 (1000G panel) and 0.008 (UKB panel) at a Benjamini-Hochberg (BH)-adjusted P-value threshold of 0.05. Conversely, Popcorn and XPASS exhibited inflated FDRs of 0.13 and 0.47 (1000G panel), and 0.15 and 0.46 (UKB panel), respectively. With a BH-adjusted P-value threshold of 0.05, Logica achieved powers of 0.41 (1000G panel) and 0.413 (UKB panel), higher than Popcorn (0.36 for both panels) but lower than XPASS (0.784 for 1000G panel; 0.764 for UKB panel; Figure S32).

We also apply Logica and compared methods in real data analysis using the 1000G reference panel. Logica is robust using the 1000G reference panel, demonstrating consistent performance across different reference panels.

Consistent with observation using UKBB reference panel, Popcorn and XPASS failed to produce valid genetic correlation estimates in a large fraction of genomic regions while Logica produced estimates for all regions. When using the 1000G reference panel, Popcorn failed to provide estimates for an average of 525 out of 1,357 genomic regions (39.0%; range: 393–599 across traits). Similarly, using the UKBB reference panel, Popcorn failed for an average of 529 regions (39.0%; range: 391–611 across traits). In contrast, XPASS failed for fewer regions, averaging 316 regions (23.4%; range: 85–473 across traits) with 1000G, and averaging 317 regions (23.4%; range: 85–478 across traits) with UKBB (Figure S33).

The mean genetic correlation across regions identified by Logica was consistently high, averaging 0.93 (range: 0.88–0.96) with the 1000G reference panel and 0.96 (range: 0.91–0.97) with the UKBB panel (Fig. 4a). In contrast, Popcorn exhibited substantial variability, with mean genetic correlations ranging widely from -0.93 to 4.01 (mean 1.69) with the 1000G panel and from -0.70 to 2.11 (mean 0.89) with the UKBB panel. XPASS provided moderate estimates, averaging between 0.62 and 0.86 across traits with the 1000G panel and between 0.65 and 0.86 with the UKBB panel (Figure S34).

Logica identified an average of 143 genetically correlated regions (range: 34–522) with the 1000G panel and 175 regions (range: 47–589) with the UKBB panel. Among these genetically correlated regions, 96.5% (138/143; range: 31–522) exhibited non-zero heritability in both ancestries using the 1000G panel, while 93% (162/175; range: 47–589) showed similar results with the UKBB panel, highlighting Logica's robustness across different reference panels. Popcorn identified fewer genetically correlated regions, averaging 128 regions (range: 52–281) with 1000G and 123 regions

(range: 40–276) with UKBB, with lower percentages of regions displaying non-zero heritability in both ancestries: 78% (100/128; range: 29–270) and 79% (98/123; range: 40–276), respectively. In contrast, XPASS identified substantially higher numbers of genetically correlated regions—averaging 895 (range: 673–1185) with the 1000G panel and 894 (range: 677–1184) with the UKBB panel—but with significantly lower percentages of these regions showing non-zero heritability in both ancestries (38% for both panels; 345/895 with 1000G and 344/894 with UKBB; Figure S35). The illustrative example from UKBB can also be replicated with the 1000G reference panel. Specifically, the genetically correlated region for LDL at 43.1–44.6 MB on chromosome 19 was consistently identified by Logica as significant. With the UKBB reference panel, Logica identified significant genetic correlation (γ_g = 0.48, P-value = 1.56×10^{-18}), whereas XPASS (γ_g = 0.22, P-value = 0.007) and Popcorn (γ_g = -0.005, P-value = 0.99) failed to detect such a signal. Similarly, using the 1000G reference panel, Logica again confirmed significant genetic correlation (γ_g = 0.42, P-value = 1.2×10^{-46}), whereas XPASS (γ_g = 0.07, P-value = 0.33) and Popcorn (γ_g = -0.01, P-value = 0.97) failed to detect such a signal (Figure S36).

Supplemental Note 5

Upward Bias in the Presence of Population-Specific Variants

We conducted additional simulations to quantify potential biases arising from population-specific variants. In our simulation study, we randomly sampled 100 regions and selected 10% of SNPs with the lowest minor allele frequencies from each ancestry to serve as ancestry-specific variants. The remaining 80% of SNPs were considered common variants and used in the genetic correlation analyses. Thus, SNPs were categorized into three groups: European-specific (X_{EUR}), East Asian-specific (X_{EAS}), and common SNPs (X_{Common}). We simulated the effect sizes for common SNPs from a bivariate normal distribution following our previously described simulation pipeline, setting genetic correlation parameters γ_g at 0, 0.25, 0.5, 0.75, and 1. For the ancestry-specific SNPs, we simulated effects from a univariate normal distribution. The total heritability explained by the region was set at 3×10^{-4} and 5×10^{-4}.

The simulated effects for each ancestry were structured such that, for European ancestry (β_{EUR}), the effect sizes consisted of $\beta_{EUR_{Common}}$ for common SNPs, $\beta_{EUR_{Specific}}$ for European-specific SNPs, and zeroes (β_{EUR_0}) for East Asian-specific SNPs. Similarly, for East Asian ancestry (β_{EAS}),

the effect sizes consisted of $\beta_{EAS_{Common}}$ for common SNPs, zeroes (β_{EAS_0}) for European-specific SNPs, and $\beta_{EAS_{Specific}}$ for East Asian-specific SNPs. The true genetic correlation for the region was quantified using the correlation between β_{EUR} and β_{EAS}. Consequently, due to the inclusion of ancestry-specific variants, the effective correlation between ancestries was inherently smaller than the specified γ_g values. The true genetic correlations, quantified by the correlation between β_{EUR} and β_{EAS}, were 0, 0.22, 0.44, 0.66, and 0.89 for γ_g values of 0, 0.25, 0.5, 0.75, and 1, respectively. All simulations were conducted under a polygenic architecture.

Our simulation analysis indicated that the genetic correlation estimates were generally slightly overestimated for all methods (Figure S26). Specifically, the mean estimated genetic correlations were 0.03, 0.27, 0.50, 0.71, and 0.94 for Logica; 0, 0.24, 0.62, 0.87, and 1.18 for Popcorn; and 0, 0.22, 0.44, 0.67, and 0.93 for XPASS.

Supplemental Note 6

Logica Extension to Admixed Population

We developed an extension of Logica to accommodate admixed populations by explicitly modeling genetic correlation through local ancestry dosage. Specifically, we considered the following association model in the admixed population:

$$\boldsymbol{y} = \boldsymbol{X_1 b_1} + \boldsymbol{X_2 b_2} + \boldsymbol{\epsilon},$$

where $\boldsymbol{y}$ is the standardized phenotype vector of length N, and $\boldsymbol{X}_1, \boldsymbol{X}_2$ are the N by m haplo-genotype matrix for two ancestral populations. Entries x_{ij1} and x_{ij2} represent the (i,j)*-th* elements of $\boldsymbol{X}_1$ and $\boldsymbol{X}_2$, which denote the minor allele counts for the *ith* individual and *jth* SNP, specific to each local ancestry, respectively. Due to admixture, x_{ij1} and x_{ij2} are defined as follows:

$$x_{ij1} := x_{i,j,M}\mathrm{I}\big(h_{i,j,M} = 1\big) + x_{i,j,P}\mathrm{I}(h_{i,j,P} = 1)$$

$$x_{ij2} := x_{i,j,M}\mathrm{I}\big(h_{i,j,M} = 2\big) + x_{i,j,P}\mathrm{I}(h_{i,j,P} = 2)$$

where $x_{i,j,M}$ and $x_{i,j,P}$ as number of minor alleles at maternal and paternal haplotypes, respectively. We denote corresponding local ancestries as $h_{i,j,M}, h_{i,j,P} \in \{1,2\}$. $\mathrm{I}(\cdot)$ denotes the indicator function. In the special case of a single ancestral population without admixture, the above model simplifies to one of the two core equations in Logica.

Following the main effect size assumptions in Logica, we also assume that SNP effects for the two ancestries follow a bivariate normal distribution:

$$\begin{pmatrix}\beta_{1j}\\ \beta_{2j}\end{pmatrix} \sim BN\left(\begin{pmatrix}0\\0\end{pmatrix}, \begin{pmatrix}\frac{h_1^2}{m} & \frac{\rho_g}{m}\\ \frac{\rho_g}{m} & \frac{h_2^2}{m}\end{pmatrix}\right)$$

where h_1^2 and h_2^2 represent the local heritability of trait in the two ancestries; and ρ_g represents the local genetic covariance, which characterizes the phenotypic covariance explained by genetic effects. The genetic correlation is defined as $\gamma_g = \frac{\rho_g}{\sqrt{h_1^2 h_2^2}}$. We propose an expectation-maximization algorithm for above model. We denote parameters $\theta = \{h_1^2, h_2^2, \rho_g, \sigma_e^2\}$.

To derive the EM updates, we first write the complete-data log-likelihood,

$$l(\theta; y, \beta|X_{LA}) = -\frac{N+m}{2}\log 2\pi - \frac{N}{2}\log\sigma_e^2 - \frac{1}{2\sigma_e^2}(y - X_{LA}\beta)^T(y - X_{LA}\beta) - \frac{m}{2}\log|V| - \frac{1}{2}\beta^T(V^{-1}\otimes I_m)\beta,$$

where $X_{LA} = [X_1 \quad X_2]$ denotes the concatenated local dosage matrix formed by the two ancestry-specific genotype matrices.

E-step

At iteration *t*, the conditional distribution of β given the data and the current value of $\sigma_e^{2^{(t)}}$ and $V^{(t)}$ with

$$\beta|y, X_{LA}, \theta^{(t)} \sim N\left(\hat{\mu}_\beta^{(t)}, \hat{\Sigma}_\beta^{(t)}\right),$$

where,

$$\hat{\mu}_\beta^{(t)} = \frac{1}{\sigma_e^{2^{(t)}}}\hat{\Sigma}_\beta^{(t)}[X_{LA}^T y],$$

$$\hat{\Sigma}_\beta^{(t)} = \left[\frac{X_{LA}^T X_{LA}}{\sigma_e^{2^{(t)}}} + \left(V^{(t)}\right)^{-1}\otimes I_m\right]^{-1}.$$

Partitioning $\hat{\mu}_\beta^{(t)} = \left[\hat{\mu}_{\beta 1}^{(t)^T} \quad \hat{\mu}_{\beta 2}^{(t)^T}\right]^T$ and $\hat{\Sigma}_\beta^{(t)} = \begin{bmatrix}\hat{\Sigma}_{\beta 11}^{(t)} & \hat{\Sigma}_{\beta 12}^{(t)}\\ \hat{\Sigma}_{\beta 21}^{(t)} & \hat{\Sigma}_{\beta 22}^{(t)}\end{bmatrix}$ into blocks, then for jth SNP,

$$C_j^{(t)} = E\left[\beta_j\beta_j^T|y, \sigma_e^{2^{(t)}}, V^{(t)}\right] = \hat{\mu}_{\beta j}^{(t)}\hat{\mu}_{\beta j}^{(t)^T} + \hat{\Sigma}_{\beta j}^{(t)},$$

Summing over SNPs yields,

$$S^{(t)} = \sum_{j=1}^{m} C_j^{(t)}.$$

M-step

Taking expectation of the complete-data log-likelihood with respect to the conditional distribution of β given y and the current value of $\sigma_e^{2^{(t)}}$ and $V^{(t)}$ is:

$$Q\left(\theta \middle| y, \sigma_e^{2^{(t)}}, V^{(t)}\right) \coloneqq E_{\beta|y,X_{LA},\sigma_e^{2^{(t)}},V^{(t)}}[l(\theta; y, \beta|X_{LA})]$$

$$= \frac{N+m}{2}\log 2\pi - \frac{N}{2}\log \sigma_e^{2^{(t)}} - \frac{m}{2}\log|V| - \frac{1}{2\sigma_e^{2^{(t)}}} y^T y$$

$$-\frac{1}{2}\hat{\mu}_\beta^{(t)^T}\left(\frac{X_{LA}^T X_{LA}}{\sigma_e^{2^{(t)}}} + \left(\left(V^{(t)}\right)^{-1} \otimes I_m\right)\right)\hat{\mu}_\beta^{(t)}$$

$$-\frac{1}{2}tr\left\{\left(\frac{X_{LA}^T X_{LA}}{\sigma_e^{2^{(t)}}} + \left(V^{(t)}\right)^{-1} \otimes I_m\right) * \hat{\Sigma}_\beta^{(t)}\right\} + \frac{\hat{\mu}_\beta^{(t)^T} X_{LA}^T y}{\sigma_e^{2^{(t)}}}.$$

Maximizing with respect to V gives

$$\frac{\partial}{\partial V^{-1}} Q\left(\theta \middle| y, \sigma_e^{2^{(t)}}, V^{(t)}\right) = -\frac{m}{2} trace(V) + \frac{1}{2} trace\left(S^{(t)}\right)$$

$$V^{(t+1)} = \frac{1}{m} S^{(t)} = \frac{1}{m}\sum_{j=1}^{m} C^{(t)}$$

Maximizing with respect to σ_e^2 gives

$$\frac{\partial}{\partial \sigma_e^2} Q\left(\theta \middle| y, \sigma_e^{2^{(t)}}, V^{(t)}\right) = -\frac{N}{2\sigma_e^2} + \frac{1}{2}\left[\left(y - X_{LA}\hat{\mu}_\beta^{(t)}\right)^T\left(y - X_{LA}\hat{\mu}_\beta^{(t)}\right) + tr\left(X_{LA}\hat{\Sigma}_\beta^{(t)} X_{LA}^T\right)\right]$$

$$\sigma_e^{2^{(t+1)}} = \frac{1}{N}\left[\left(y - X_{LA}\hat{\mu}_\beta^{(t)}\right)^T\left(y - X_{LA}\hat{\mu}_\beta^{(t)}\right) + tr\left(X_{LA}\hat{\Sigma}_\beta^{(t)} X_{LA}^T\right)\right]$$

To evaluate this extension, we conducted simulation studies using genotype data from Black-British individuals in the UK Biobank. First, we inferred genome-wide ancestry proportions using SCOPE[1] with 1000 Genomes Phase 3 data as reference. We retained 3,321 individuals exhibiting admixed ancestry primarily from AFR and EUR populations. Next, we randomly selected 100 genes across the genome, extending each region by 50 kb upstream and downstream, and inferred local ancestry dosage using RFMix2[2]. Phenotypic effects were simulated with local heritability parameter with the Nh^2 set to either 50 or 100 corresponds to local heritability of 0.015 and 0.30, and genetic correlation parameters γ_g set at 0, 0.25, 0.5, 0.75, and 1.

Simulation results demonstrated that our extended Logica approach accurately and robustly estimated both local heritability and local genetic correlations across various simulation scenarios (Figure S25). We first evaluated the accuracy of local heritability estimates. When the true heritability was 0.015 for both ancestries, Logica provided mean estimates of 0.0172 (MSE = 9.38×10^{-5}) and 0.0172 (MSE = 7.38×10^{-5}) across regions. Similarly, when the true heritability increased to 0.030, Logica yielded mean estimates of 0.0350 (MSE = 2.85×10^{-4}) and 0.0362 (MSE = 2.88×10^{-4}). We next assessed the accuracy of local genetic correlation estimates. For true genetic correlations of 0, 0.25, 0.5, 0.75, and 1, Logica produced mean estimates of 0.00655 (MSE = 4.29×10^{-5}), 0.251 (MSE = 1.44×10^{-6}), 0.494 (MSE = 3.53×10^{-5}), 0.722 (MSE = 7.89×10^{-4}), and 0.900 (MSE = 9.51×10^{-3}), respectively.

Supplemental References

1. Chiu, A. M., Molloy, E. K., Tan, Z., Talwalkar, A. & Sankararaman, S. Inferring population structure in biobank-scale genomic data. *Am J Hum Genet* **109**, (2022).

2. Berisa, T. & Pickrell, J. K. Approximately independent linkage disequilibrium blocks in human populations. *Bioinformatics* **32**, (2016).
